# LINE-1 mRNA 3′ end dynamics shape its biology and retrotransposition potential

**DOI:** 10.1093/nar/gkad1251

**Published:** 2024-01-09

**Authors:** Damian M Janecki, Raneet Sen, Natalia Szóstak, Arkadiusz Kajdasz, Martyna Kordyś, Kinga Plawgo, Dmytro Pandakov, Anna Philips, Zbigniew Warkocki

**Affiliations:** Department of RNA Metabolism, Institute of Bioorganic Chemistry, Polish Academy of Sciences, Poznan, Poland; Department of RNA Metabolism, Institute of Bioorganic Chemistry, Polish Academy of Sciences, Poznan, Poland; Laboratory of Bioinformatics, Institute of Bioorganic Chemistry, Polish Academy of Sciences, Poznan, Poland; Department of RNA Metabolism, Institute of Bioorganic Chemistry, Polish Academy of Sciences, Poznan, Poland; Department of RNA Metabolism, Institute of Bioorganic Chemistry, Polish Academy of Sciences, Poznan, Poland; Department of RNA Metabolism, Institute of Bioorganic Chemistry, Polish Academy of Sciences, Poznan, Poland; Department of RNA Metabolism, Institute of Bioorganic Chemistry, Polish Academy of Sciences, Poznan, Poland; Laboratory of Bioinformatics, Institute of Bioorganic Chemistry, Polish Academy of Sciences, Poznan, Poland; Department of RNA Metabolism, Institute of Bioorganic Chemistry, Polish Academy of Sciences, Poznan, Poland

## Abstract

LINE-1 (L1) retrotransposons are mobile genetic elements that create new genomic insertions by a copy-paste mechanism involving *L1* RNA/RNP intermediates. *L1* encodes two ORFs, of which L1-ORF2p nicks genomic DNA and reverse transcribes *L1* mRNA using the nicked DNA as a primer which base-pairs with poly(A) tail of *L1* mRNA. To better understand the importance of non-templated *L1* 3′ ends’ dynamics and the interplay between *L1* 3′ and 5′ ends, we investigated the effects of genomic knock-outs and temporal knock-downs of *XRN1*, *DCP2*, and other factors. We hypothesized that in the absence of XRN1, the major 5′→3′ exoribonuclease, there would be more *L1* mRNA and retrotransposition. Conversely, we observed that loss of XRN1 decreased L1 retrotransposition. This occurred despite slight stabilization of *L1* mRNA, but with decreased *L1* RNP formation. Similarly, loss of DCP2, the catalytic subunit of the decapping complex, lowered retrotransposition despite increased steady-state levels of L1 proteins. In both XRN1 and DCP2 depletions we observed shortening of *L1* 3′ poly(A) tails and their increased uridylation by TUT4/7. We explain the observed reduction of L1 retrotransposition by the changed qualities of non-templated *L1* mRNA 3′ ends demonstrating the important role of *L1* 3′ end dynamics in L1 biology.

## Graphical Abstract

**Graphical Abstract:**
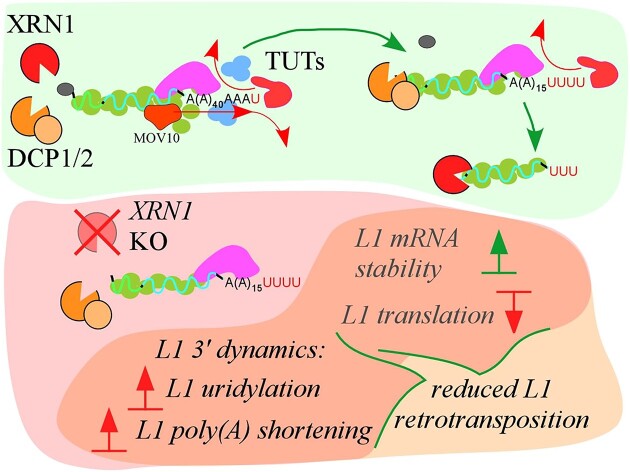

## Introduction

Retrotransposons are mobile genetic elements that copy their sequences and insert them in new genomic locations via a mechanism called retrotransposition. Sequencing of the human genome has led to a discovery that nearly half of it derives from repetitive sequences, mostly of retrotransposonal origin (1,2). Retrotransposons in the human genome comprise long-terminal repeat retrotransposons (LTRs), also known as endogenous retroviruses (ERVs), and non-long terminal repeat retrotransposons including long-interspersed elements (*L1*), short-interspersed elements (*Alu*), and a class of *SVA* retrotransposons (3,4). Around 516 000 copies of *L1* occupy nearly 17% of the entire human genome (1,5,6). Most of these copies are mutated and thus incapable of mobilization (7). A small fraction of up to 100 copies can still mobilize in modern human genomes and provides molecular machinery for mobilization of *Alu* and *SVA* (8–10). A full-length *L1* element is ∼6 kb, possesses its own promoter in the 5′ UTR and encodes two ORFs that translate into L1-ORF1p and L1-ORF2p proteins (11–13). L1-ORF1p is a chaperone protein that forms trimers through its coiled-coil motif (14–16). In a kinetically driven manner L1-ORF1p packs onto *L1* mRNA from which it had translated (17,18), leading to formation of cytoplasmic L1 RNP aggregates which we call L1 bodies throughout the paper (19–22). L1-ORF2p is a ∼150 kDa protein with two enzymatic activities: endonuclease and reverse transcriptase (23–25). Furthermore it creates multiple protein-protein and protein-RNA interaction sites (26). A new *L1* insertion occurs through target-primed reverse transcription (TPRT) which commences by nicking of the genomic DNA by the L1-ORF2p endonuclease activity (27–30). The short single stranded genomic DNA fragment released by the nicking is mostly an oligo(dT) sequence which can base-pair with the *L1* 3′ poly(A) tail constituting a primer for reverse transcription (31,32). Thus mismatches in the genomic sequence (31) or uridylation of *L1* 3′ end (33) interfere with or completely abolish TPRT initiation.

L1s are known to create new genomic pathological insertions, although the actual number of the disease-related insertions is low (34). Instead, *L1* expression and the presence of *L1* extrachromosomal cDNA, RNA, and RNPs contribute to innate immunity responses (35). *L1* expression is involved in cancers either as result of DNA demethylation and carcinogenesis or as drivers of the process (36–39). Since *L1* expression and retrotransposition pose serious danger to genomic stability and cellular homeostasis there are multiple regulatory mechanisms that restrict *L1*. Transcriptional silencing is the first line of defence in most somatic cells (40,41), while in gametogenesis and early embryo development piRNA-involving mechanisms silence *L1* both on transcriptional and post-transcriptional levels (4,42,43). Despite these, *L1* transcripts are detectable in most global transcriptomic analyses and new somatic insertions continue to be identified especially in long-lived cell types as neurons (44–46). Thus other post-transcriptional mechanisms exist that prevent unfettered *L1* activity (47,48).

Canonical mRNAs, including *L1* mRNA, possess special elements at their ends: the 5′ cap and the 3′ poly(A) tail, that both protect mRNA from degradation and ensure efficient translation (49–51). Most mRNA decay by a conserved step-wise mechanism involving deadenylation (shortening) of their poly(A) tails, followed by uridylation by TUT4/7 enzymes, decapping, and ultimately, degradation by the XRN1 exoribonuclease (52,53). Uridylation was shown to stimulate decapping (54,55), and knock-down of *XRN1* leads to accumulation of uridylated mRNA (52). Translation of L1 proteins was demonstrated to depend on the 5′ methyl-G cap (11,13), and its 3′ poly(A) tail is even more important than in other mRNAs, as it serves in the initial stages of TPRT (31–33). Thus changes of its integrity, lengths, and addition of 3′ terminal non-A nucleotides (mostly uridines), which we collectively refer to as (non-templated) 3′ end dynamics, will likely significantly affect L1. Here, we analysed L1 in the context of knock-down and knock-out (KO) of *XRN1* and/or *DCP2*. The first encodes the cytoplasmic 5′→3′ exoribonuclease that degrades mRNA following its decapping by the DCP1/2 complex, in which DCP2 is the catalytic subunit (56–59). Despite the stabilisation of *L1* mRNA in the *XRN1* knock-out cells and the clear difference in the amounts of L1 proteins produced in the *XRN1* KO compared to *DCP2* KO, L1 retrotransposition is diminished in both of them as compared to the wild-type cells. Curiously, in multiple clonal *XRN1*, *DCP2* and the double *DCP2* plus *XRN1* KO cells we observe substantial shortening of the *L1* 3′ poly(A) tails and their increased uridylation. By further analysing the effect of concurrent depletion of *XRN1* or *DCP2*, with *TUT4* and *TUT7* we observed that uridylation by TUTases is important for reduction of L1 retrotransposition in the XRN1 and DCP2 depletion conditions. Thus underlining step-wise *L1* degradation. The effective poly(A) shortening and uridylation, on both endogenous *L1* and synthetic *L1* reporter mRNAs, implies that a significant fraction of *L1* mRNA 3′ ends are in the process of intensive decay. This agrees with the accumulation of oligoadenylated, uridylated *L1* 3′ ends in the *XRN1* and *DCP2* knock-out cells which in turn reduces retrotransposition, thus establishing *L1* mRNAs with such 3′ ends as poor substrates for this process in cellular environment. In sum, with a comprehensive set of experiments, we demonstrate the *L1* 3′ end dynamics and underline its important role in L1 biology.

## Materials and methods

### Cell culture

293T, HeLa HA and PA-1 cells were derived from females (kind gifts of Dr J.L. Garcia-Perez, Edinburgh). The cells were cultured in monolayers at 37°C in a humidified 5% CO2 incubator. 293T were cultured in Dulbecco's modified Eagle's medium (DMEM, Gibco; 41966052) with high glucose (4.5 g/l) supplemented with 9% (v/v) fetal bovine serum (FBS, Gibco; 10270106), 100 U/ml penicillin and 100 μg/ml streptomycin (Merck; P4458). HeLa HA were cultured in RPMI 1640 supplemented with FBS and antibiotics as above and non-essential amino acids (NEAA, Gibco; 11140050). PA-1 were cultured in MEM (Gibco; 42360024), supplemented with heat-treated FBS (Gibco; A3840402), 2 mM l-glutamine (Gibco; 25030081), NEAA and antibiotics. The cell lines were authenticated by STR profiling (Eurofins Genomics) and by using the DSMZ CellDive online interface (https://celldive.dsmz.de/) (60). The chromatograms of the STR profiling are included at the end of the supplementary materials. Independently raised 293T *XRN1*, *DCP2* and *XRN1* plus *DCP2* knock-out cell lines (61) were kind gifts of Dr Sarah Slavoff.

### Generation of *XRN1* knock-out cells

**Figure 1.**
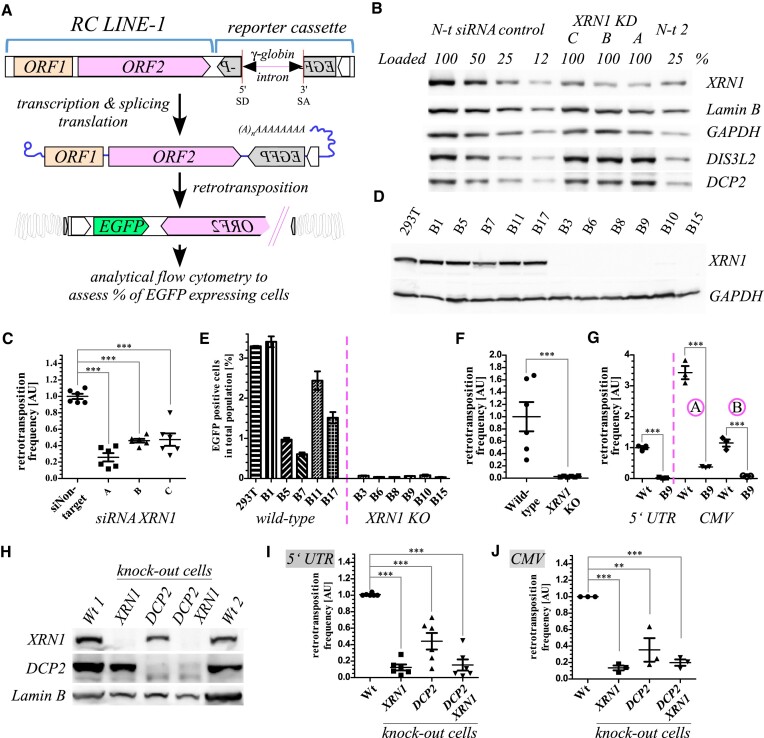
The effects of XRN1 and DCP2 deficiency on L1 retrotransposition. (**A**) Graphical representation of the rationale of the L1 retrotransposition assay in cultured cells. A full-length RC *L1* appended with a retrotransposition reporter cassette comprising a reporter gene interrupted with an intron is delivered to cell on a plasmid. Following transcription and removal of an intron from the reporter gene, the reporter can undergo retrotransposition. Only cells in which retrotransposition had occurred produce the reporter e.g. EGFP. (**B**) Western blot analysis of the RNAi-mediated depletion of XRN1 in 293T cells co-transfected with the siRNAs and the L1 reporter plasmid. A titration of the non-targeting controls is provided to help assess the depletion levels and the loading controls are indicated. Panels with probing for XRN1, lamin B, GAPDH (both loading controls), and related RNA processing factors including DCP2 and DIS3L2 are shown on pieces of the same blot. (**C**) L1 retrotransposition assay result following depletion of XRN1. Data are shown as mean ± SEM. (**D**) Western blot validation of *XRN1* KO in 293T clonal cell lines. Probing for XRN1 and GAPDH (loading control) are pieces of the same blot. Respective clones are individually named as indicated (throughout the paper). (**E**) Results of L1 retrotransposition assay using the respective clonal cell lines. Data are shown as means of 2 technical replicates ± SEM. (**F**) Results presented in panel E following normalization to the mean of the wild-type condition. Data are shown as mean (of the mean values in panel E) ± SEM. Unpaired t-test was used to calculate statistical significance. (**G**) Results of L1 retrotransposition assay by using reporters comprising either wild-type L1 5′ UTR promoter and SV40 polyadenylation signal (pAS), or a CMV promoter and HSV pAS instead. A – a reporter comprising CMV promoter and full-length RC*L1*, B – a reporter with *L1-ORF1* tagged with *mCherry*. A dashed pink line separates results with the regular L1 5′ UTR-driven reporter from the results with the CMV-driven reporters. Results were normalized to mean of the wild-type control in the assay with the regular L1 5′ UTR-driven reporter. Data are shown as mean ± SEM. Statistical significance was calculated by unpaired *t*-tests. (**H**) Western blot validation of the independently generated *XRN1*, *DCP2* and the double *DCP2* plus *XRN1* KO cell lines (61) as indicated. (**I**) Results of the L1 retrotransposition reporter assay in the KO cell lines of panel H. Regular L1 5′ UTR-driven reporter was used. Data in panels I and J are shown as mean ± SEM and statistical significance was calculated by repeated-measures ANOVA and Tukey's multiple comparison post-test. (**J**) As in panel I but a CMV-driven L1 reporter was used.

Synthetic DNA oligonucleotides were annealed and cloned into BbsI-digested pSpCas9(BB)-2A-puro plasmid according to (62). The guide sequences were: GTATAATTCCATTCATATCC (designed by an online Synthego tool; https://design.synthego.com/#/), and AGAGAAGAAGTTCGATTTGG (59), to derive pZW282, and pZW283 plasmids respectively. Cells at 90 000 per well of a 12-well plate were transfected with 0.5 μg of each plasmid (1 μg total) for 293T cells or 1 μg of the pZW283 for the HeLa HA cells. Next day selection with 1 μg/ml (293T) or 2 μg/ml (HeLa HA) puromycin started. After 3 days, half of the cells were harvested, gDNA isolated and used for PCR with primers (Supplementary Table 1) to assess editing efficiency. The rest of the cells were diluted to ∼1.25 cell per 200 μl and seeded onto 96-well plates for clonal selection. Medium was changed every 2–3 days. After 5 days, wells were inspected visually and wells with cells were marked. At 7 days post-seeding number and approximate location of the colonies were marked. After 15–20 days post-seeding cells from wells marked as ‘single colonies’ were further expanded sequentially into bigger wells (96-well into 24-well, then 12-well and 6-well plates) followed by a western blot testing the presence of XRN1 (at the 24-well plate stage). For selected clones gDNA was isolated and genotyped, cells were used for L1 retrotransposition assays (Figure 1E). Aliquots of cells were suspended in fresh medium supplemented with 10% DMSO, flash frozen and long-term stored in liquid nitrogen. Following restoration cells were used in other experiments. Genotyping was done by PCR, sequencing, and by using an online tool ICE (Synthego; https://ice.synthego.com/#/). Also see Supplementary Note 1.

### Flow cytometry

Analytical flow cytometry (FC) was done using Attune NxT (A24861; ThermoFisher Scientific) equipped with blue, λ = 488 nm, and yellow, λ = 561 nm, lasers and the producers’ band pass filter configurations for detection: λ = 530/30 nm (EGFP), λ = 620/15 nm (mCherry, PI, Alexa568). Control non-transfected (*NT*) cells were analysed to set background fluorescence (≤0.05% of fluorescent cells in the *NT*). Unless specified differently, the cells were detached from the dish by trypsinisation, suspended in full medium, centrifuged at 400 rcf for 3 min, suspended in PBS and analysed. A minimum of 10 000 (or 20 000 retrotransposition assays) live single cells were analysed. A live cell population was set by gating FSC-A and SSC-A (‘live cells’ gate), followed by subgating the ‘live cells’ by FSC-A (area) versus FSC-H (peak height) to obtain ‘singlets’ gate (single cells) (Supplementary Figure 1A). Gating EGFP fluorescence was done on the ‘singlets’ by using a histogram and setting gate as to exclude non-fluorescent cells (see Supplementary Figure 1A).

### Retrotransposition assays and control experiments

293T cells were seeded at 150 000 per well into 12-well plates in medium without antibiotics. Next day, the cells were transfected with 1 μg 99_PUR_RPS_EGFP (63) or pZW_L1RP_megfpI_HSVpAS (pZW126) or pZW_L1RP-O1mCh-megfpI_HSVpAS (pZW128) (33). Next day medium was changed for selective with 1μg/ml puromycin (Invivogen) (and penicillin/streptomycin) but only in the case of the 99_PUR_RPS_EGFP -transfected cells. After 3–4 days of selection, with a single medium change, the cells were analysed by flow cytometry (FC). For Supplementary Figure 2G, H, 90% of the cells were used for analysis by FC, and the remaining 10% of the cells were left in the wells and cultivated for 6–7 more days in medium without puromycin, followed by FC. Control non-transfected (*NT*) cells were analysed to set background fluorescence and the EGFP-positive gate (Supplementary Figure 1A). We generally observed ∼0.5–5% of EGFP-positive cells in the control conditions (293T; used as controls throughout the paper). Where biological replicates were assayed in one experiment absolute numbers (% EGFP + singlets) are given. Where biological replicates were assayed in two or more independent experiments, normalization was done. Normalization was done by setting means of control conditions (if technical replicates were done within a biological replicate) or setting the control condition to 1.00, and normalizing all other test conditions to the control. For the RNAi experiments cells were co-transfected with 1 μg 99_PUR_RPS_EGFP and a total of 20 pmol siRNA. Following siRNA were used in this study, all stealth siRNA (Invitrogen): controls (non-targeting ref. no.: 12935400, 462000; TUT1 – HSS127841 (33)), XRN1 (HSS122909, HSS182510, HSS182511), DCP2 (HSS136534, HSS136535), TUT4 (HSS146317, HSS177328), TUT7 (HSS149224, HSS149225). Control experiments to test transfection efficiencies were set by transfecting the cells with 0.25 μg of pKK-TEV-mCherry and/or pKK-TEV-EGFP (64). HeLa HA cells were seeded at 25 000 (WT) or 50 000 (*XRN1* KO) per well of a 6-well plate in an antibiotic-free medium. Next day, the cells were transfected with either 1 μg JM101/L1.3 mneoI (L1 retrotransposition assay), pT2 neoI (encoding resistance gene towards G418, positive control of G418 selection, kind gift of Dr J.L. Garcia-Perez), or pKK-TEV-EGFP (64) (negative control of G418 selection) using 8 μl Fugene6 (Promega) per well. Next day and day 3 post-transfection (p-t), the medium was changed for one containing penicillin/streptomycin. On day 4 p-t, the medium was changed for one containing 450 μg/ml G418 (Invivogen). The selection continued for 14 days with medium change every second day. After that medium was removed, cells washed with PBS, fixed with cold methanol, stained with crystal violet, and photographed.

### Analysing cell cycle and apoptosis

For cell cycle analysis, cells were washed with PBS, fixed and permeabilized in a cold 100% methanol on ice for 15 min and stained with 50 μg/ml propidium iodide (Sigma Aldrich) solution in PBS containing 100 μg/ml RNase A (Sigma Aldrich) at 37°C for 15 min. Cells were additionally incubated in the staining solution on ice for 1 h. The DNA content was measured using Attune NxT. Data were analysed on ModFit LT™ 6.0 software (Verity Software House). For detection of apoptosis, cells were stained using the Annexin V-AlexaFluor568 conjugate (Thermo Fisher Scientific) according to the manufacturer's protocol and signal measured by FC.

### Cloning

Cloning was done using sequence and ligation independent cloning (SLIC) into a pKK_no_tag plasmid (64), a derivative of pcDNA5/FRT/TO (ThermoFisher Scientific). *XRN1* wild-type and mutant genes were amplified by PCR (Supplementary Table 1). The mutant genes were generated by including mutations in primers and were amplified using a splice PCR approach (64) to join the fragments. The plasmids purified by minipreps (A&A Biotechnology) were transfected into 293T cells, followed by western blot to assess expression of full-length XRN1. Chosen clones were confirmed by sequencing.

### XRN1 rescue experiments

**Figure 2.**
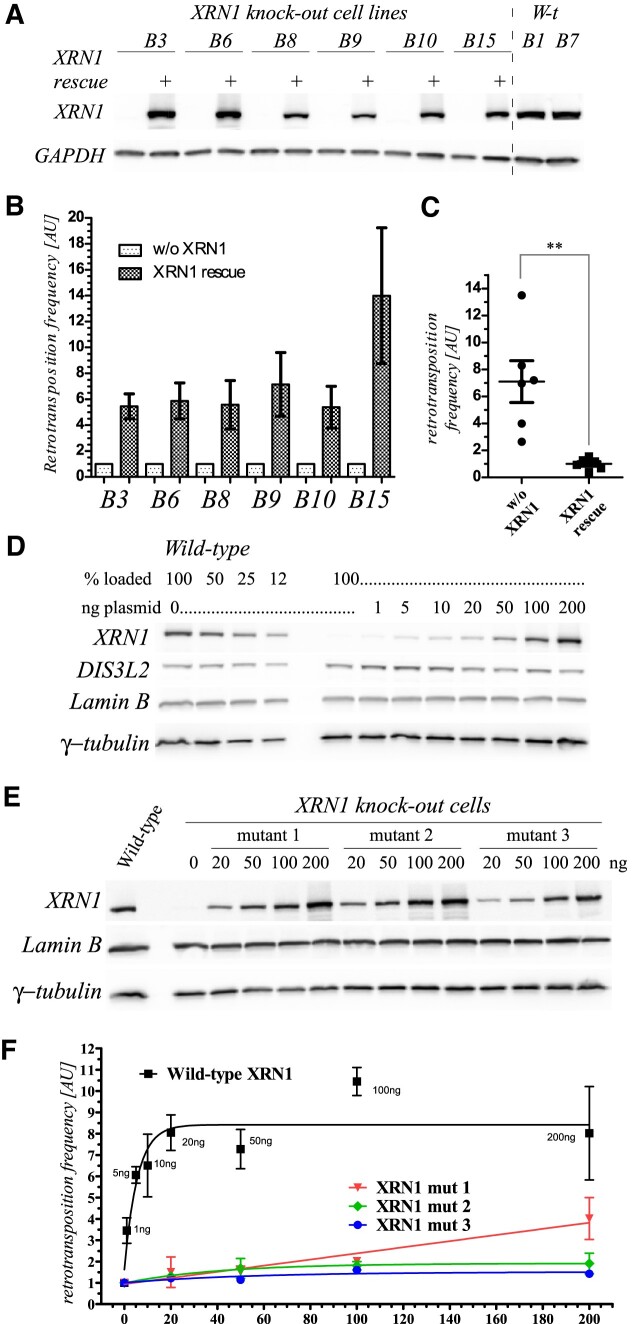
*Rescue of L1 retrotransposition in the XRN1 knock-out 293T cells*. (**A**) Western blot validation of XRN1 expression in the 293T *XRN1* KO cell lines following transfection with a wild-type untagged XRN1 expressing plasmid as indicated. Two wild-type cell lines added for comparison. GAPDH is the loading control. (**B**) Result of 4 independent L1 retrotransposition assays in the *XRN1* KO cells in the absence or presence of ectopically expressed wild-type XRN1. Levels of L1 retrotransposition without XRN1 rescue were normalized to 1.00 for each individual cell line. Shown are mean L1 retrotransposition levels in the independent experiments ± SEM. (**C**) Data of panel B. Levels of L1 retrotransposition without XRN1 rescue for all cell lines (not individually as in panel B) was normalized to 1.00. Data are shown as mean ± SEM. Statistical significance confirmed using unpaired *t*-test. (**D**) Western blot validation of the expression of the wild-type XRN1 protein in the experiment shown in panel D. Expression of endogenous Lamin B and γTubulin proteins was used as loading controls. All probing were done with parts of the same blot. (**E**) Western blot validation of the expression of the mutant XRN1 proteins in the experiment shown in panel F. (**F**) L1 retrotransposition assay with a titration of plasmid encoding untagged either wild-type or mutant XRN1 protein as indicated. The amounts of XRN1-encoding plasmids in each data point are indicated. Fitting was done using Prism software. Data are shown as mean ± SEM.

The XRN1 rescue experiments were carried out as described in the section on retrotransposition assays, with modification: 750 ng 99_PUR_RPS_EGFP and 200 ng *XRN1* encoding plasmid (wild-type or the mutants) were used to transfect the cells. The total of 200 ng XRN1 plasmid were also used in the titration experiments. In these experiments respective amounts of a plasmid encoding a non-functional *XRN1* gene (a frameshift precluding expression of XRN1) was used as a ‘balance’, to ensure a total of 200ng of plasmid DNA (plus 750 ng of the reporter) were delivered to cells in all conditions. 293T and B9 cell lines were used to prepare Figure 2F.

### RT-qPCR and actinomycin D time course experiment

Cells were transfected with the 99_PUR_RPS_EGFP plasmid as described in the section on the L1 retrotransposition assay. Total RNA was isolated using a home-made Trizol reagent according to (65) and chloroform extraction, DNA was removed by treatment with TURBO Dnase (Ambion) according to the manufacturer's protocol and purified again by phenol–chloroform extraction. Reverse transcription was performed on 1μg total RNA with SuperScript III (ThermoFisher Scientific) according to manufacturer's protocol. The qPCR were performed as described (33) using the TaqMan assay (Supplementary Table 1; predesigned GAPDH assay with VIC probe – AppliedBiosystems; 4448490). The time course experiment to assess L1 stability was performed as described (33) following transfection with the 99_PUR_RPS_EGFP plasmid. Actinomycin D (Carl Roth) was added to 5μg/ml for the indicated amounts of time.

### Northern blots

Cells were seeded at ∼500 000 onto wells of a 6-well plate in medium without antibiotics. Next day cells were transfected with 2 μg JM101/L1.3 nomarker (18) (kind gift of Dr J.L. Garcia-Perez) using 7 μl Lipofectamine 2000 per well. Two days post-transfection, RNA was isolated, DNase-treated and ∼10 μg separated in a single well of a 1% denaturing agarose gel according to (66). RNA were capillary transferred to a Hybond N+ nylon membrane (Amersham) and fixed by UV-crosslinking. Membranes were stained with methylene blue and photographed. Hybridization was done in PerfectHyb hybridization buffer (Sigma) with probes as described (33).

### Assessing translation of tagged ORF1

Ca. 100 000–200 000 cells per well of a 12-well plate were transfected with 1 μg either pZW-L1RP-O1mCh (pZW127), pVAN583 (pZW130), pZW-L1RP-O1F (pZW125), or JM101/L1.3 nomarker using 2.5 μl Lipofectamine 2000. After 40–48 h, the cells were analysed by FC, and/or sampled for western blot. Median intensities, per cents of FP-positive cells and other parameters were recorded and analysed as indicated.

### Western blots

Cell lysates were prepared as described (33), proteins separated by PAGE on either home-made SDS-gels or Novex 4–20% gradient Tris-glycine gels (ThermoFisherScientific), transferred onto Protran nitrocellulose membrane (Amersham, 0.45 μm) by wet transfer in a buffer supplemented with methanol to 20%. Membranes were stained with ponceau S solution, blocked in 5% low-fat milk in TBST_20_ and probed with primary antibodies in 1:1000–1:4000 dilutions in 5% skimmed milk in TBST_20_ for 16–48 h at 6°C. Following washing 3 times with TBST_20_, goat secondary antibodies (Jackson Immunoscience; 0.8 mg/ml) HRP-coupled against rabbit (111-035-144) or mouse (115-035-146) were used in 1:10 000 (γH2AX, L1-ORF2p, XRN1, TUT4/7) or 1:30 000 (other Abs) dilutions for at least 2 h at RT or overnight at 6°C. Clarity western ELC substrate (Bio-Rad) was used for chemiluminescence and recorded on Uvitec Q9 Alliance CCD camera. Primary antibodies against: XRN1 (Bethyl; A300-443A & Proteintech; 23108-1-AP), lamin B (Proteintech; 12987-1-AP), GAPDH (Novus Biologicals; NB300-327), DIS3L2 (Proteintech; 67623-1-Ig), DCP2 (Proteintech; A302-597A-T & Invitrogen; PA5-115102), γ-tubulin (Sigma; T6557), L1Hs-ORF1p ((67), kind gift of Dr J.L. Garcia-Perez), L1-ORF2p (MT49 (68), kind gift of drs Kathleen Burns & Martin Taylor), γH2AX (Bethyl; A700-053-T), GFP (SantaCruz; sc-9996), PABPC1 (Proteintech; 10970-1-AP), TUT7 (Sigma; HPA020620), TUT4 (Proteintech; 18980-1-AP).

### Assessment of L1 3′ ends lengths by RNAse H

Reactions were carried out in 50 μl. Total RNA, 8–10 μg, from the wild-type and the *XRN1* KO cells following treatment with TURBO Dnase (Ambion) were mixed with ORF2_RH1 DNA oligonucleotide, with or without oligo(dT)_15_ DNA to final concentrations of 0.1 μM and 0.2/0 μM, respectively. Nucleic acids were denatured for 1 min at 95°C and placed in ice. 10× RNase H reaction buffer (NEB) and 0.5 μl RNase H (NEB; M0297) were added. Reactions were carried out for 30 min at 37°C, then stopped by phenol-chloroform extraction, and precipitated with ethanol in the presence of glycoblue coprecipitant. RNA were separated in a denaturing 1% agarose gel with formaldehyde (0.45 M), blotted by capillary transfer onto a Hybond N+ nylon membrane (Amersham) and probed with 5′ ^32^P-labelled 3UTR_RH1 oligonucleotide as described in the northern blot procedure.

### Confocal microscopy

Cells were grown on poly-l-lysine covered glass slides for 48 h, followed by washing with PBS (3 times), fixation with 3.7% formaldehyde solution and 5% sucrose in PBS for 15 min, washing with PBS, staining of the nuclei with 1 μg/ml Hoechst 33342 (Biotium) in PBS for 10 min at RT, and washing twice with PBS. Slides were mounted using ProLong Gold Antifade on supports. Z-stacks were recorded by using Leica TCS SP5 II confocal microscope and LAS AF SP5 and LAS X SP8 software. Quantitation of L1 bodies was done by visual inspection.

### Imaging flow cytometry

A high-throughput multispectral fluorometric technique was used to analyze L1 foci in 293T cells. Two days post-transfection with pVAN583 (pZW130; pZW-L1RP-O1EGFP) or pZW127 (pZW-L1RP-O1mCh), 293T cells were harvested in PBS and stained with 2.5 μg/ml Hoechst33342 (Biotium). Over 1500 live cells were analysed for each cell line. Digital images were recorded on a multispectral imaging flow cytometer (ImageStreamX MkII instrument, Luminex) equipped with blue, λ = 488 nm, and yellow, λ = 561 nm, lasers and the producers’ band pass filter configurations for detection: Ch2 λ = 528/65 nm (EGFP) and Ch4 λ = 610/30 nm (mCherry). Fluorometric compensation was digitally calculated based on single-stain controls. Focused cells (gated in R1) were selected based on a histogram plot of the gradient root mean square (RMS) of bright-field images. The single cells (gated in R2) were chosen based on a dot plot of aspect ratio with area. Then EGFP or mCherry positive cells were gated in R3 based on intensity. Following data acquisition, images were analysed using the manufacturer's software (IDEAS® 6.3, Luminex). A fluorescent spot was defined as L1 body if diameter = 0.33 ≥ 3 μm and the fluorescent signal ≥8-fold above background. These threshold parameters were applied to the default mask of the representative cells according to the manufacturer's instructions to determine the spot count and area.

### LEAP assay

LEAP assays were performed as described (69) with modifications. Indicated 293T cells were seeded at 5 × 10^6^ cells onto 145 mm plates in medium without antibiotics. Next day, the cells were transfected with 20 μg JM101/L1.3 nomarker plasmid (18) using 52 μl Lipofectamine 2000. After 3 days, the cells from one dish were pelleted and lysed in 500 μl 0.35× PBS, 5 mM DTT, 2 mM MgCl_2_, 0.2% Igepal CA-630, supplemented with cOmplete Ultra protease inhibitors (Roche) and RNase inhibitor. Following incubation on ice for 20 min, the lysates were centrifuged twice in a table-top centrifuge at 5200 rcf at 4°C. The cleared lysates were loaded onto 13 ml SW41Ti tubes filled with sucrose cushions (7.5% and 15% sucrose in 80 mM NaCl, 2 mM MgCl_2_, 20 mM Tris 7.5, 2 mM DTT, with protease inhibitors) and spun in a SW41Ti rotor for 2 h 45 min at 273 620 rcf. Pellets were suspended in 0.1× PBS, 37% glycerol, 5 mM DTT supplemented with protease inhibitors, flash frozen and stored at –80°C. The amounts of L1 proteins in the pelleted materials were estimated by western blot and used to adjust the amount of pelleted material for reverse transcription. PCR was performed using Q5 polymerase (NEB) (for RT and PCR primers see Supplementary Table 1).

### 3′ RACE-seq library preparation and analyses

Same RNA as for the Northern blots were used. For the 12 home-made 293T cell lines (this paper) including the wild-type and the *XRN1* KO each biological replicate was a single technical replicate (one index per cell line). For the four acquired cell lines (61) three technical replicates (starting at the RT step) of each biological replicate were performed and marked with a different index (Supplementary Table 2). Libraries were prepared as described in (33) with modifications. Total RNA after DNase treatment (2 μg) were ligated with 100 pmol RA3_15N 5′ preadenylated adapter comprising a 15-nucleotide unique molecular identifier (UMI) in 20 μl of 1x enzyme-supplied buffer, 15% PEG 8000 by T4 RNA ligase 2 truncated (NEB, M0242) for 18 h at 18°C. Nucleic acids of each reaction were purified using 20 μl Ampure XP magnetic beads and used for reverse transcription in 20 μl with 100 pmol RPI primer (containing an 6-nucleotide index) using SuperScript III according to the manufacturer's protocol (2 min at 50°C, 30 min at 45°C, 15 min at 85°C), followed by purification on 1.25 volume of Ampure XP magnetic beads. Nested PCR was performed with PrimeSTAR GLX polymerase (TaKaRa) since it proved best at elongating long poly(A) tracts (compared to Phusion (ThermoFisher Scientific) and Q5 (NEB); not shown). First PCR was performed in 25 μl with an equivalent of 500 ng input total RNA (input to RT step) per reaction. Primers at 0.35 μM were either of: 3UNP1f, GAPDH_3R0, PABPC4_3R0 for L1, GAPDH and PABPC4 respectively, universal reverse primer, RP_uni, was added at 0.4 μM. Reactions were carried out: 98°C 2 min [98°C 12 s, 52°C 15 s, 70°C 40 s] × 21, 68°C 1 min, 1/5 of each reaction was used without purification as template for second PCR with 0.36 μM transcript-specific forward primer: L1_NGS4, or GAPDH_NGS, or PABPC4_NGS, and 0.4μM universal reverse primer RP_uni. PCRs were carried out: 98°C 2 min [98°C 12 s, 55°C 15 s, 70°C 30 s] × 21, 70°C 1 min. The reactions were purified on 1.3 volume Ampure XP magnetic beads, eluted, measured and diluted to ∼5 ng/μl, analysed on TapeStation (Agilent). Before running on Illumina Novaseq libraries were quantitated by qPCR to adjust the number of reads to ∼3–5 MRds for each transcript for each index. Analysing and visualising 3′ RACE-seq data was done as described in the Supplementary methods by scripts https://gitfront.io/r/pbioinf/eUKTgpxukvCY/RACE-Seq/.

### Selection of capped RNA

The production of GST-4EK119A protein and selection of capped RNA was performed as described (70), but scaled for smaller input total RNA amounts of 10–20μg. Following the washing of the beads, the capped RNA was retrieved directly from the beads by phenol–chloroform extraction.

### Assessing endogenous*L1* mRNA and proteins

Used were: (i) 293T (wild-type and *XRN1* KO cells), and (ii) PA-1 cells following transfections with siRNA, all from Invitrogen (stealth and silencer select): controls (non-targeting ref. no.: 12935200, 12935400, 4390843), XRN1 (HSS122909, HSS182511, s29016), DCP2 (HSS136534, HSS136535, S46660). For RNAi 200 000 PA-1 cells were seeded onto a well of a 6-well plate. Next day, medium was changed for one without antibiotics and cells were transfected with 20 pmol siRNA using 6 μl Lipofectamine RNAiMAX (ThermoFisher Scientific) in 200 μl OPTI-MEM (Gibco) per well. After 48 h, cells were harvested for RNA extraction by lysis in home-made Trizol reagent, cells from duplicate transfections were sampled for western blot. RT-qPCR was performed as described (33). Northern blots were performed as described (33) with 5–7 μg total RNA following treatment with Turbo DNase (Ambion), and separation in a 0.35 M formaldehyde 1% agarose gels in 1× TT buffer (33,66). 3′ RACE-seq libraries and sequencing were performed as described above but L-1repNGS1 primer was used in PCR2 and more cycles were used in PCRs to amplify the libraries (PA-1 ‘48h’ – 30/30, PA-1 ‘126h’ & 293T – 33/33). L-1repNGS1 primer could also base-pair to *L1-PA3* and -*PA4* but with reduced melting temperature (*T*_m_), and *-PA5* with reduced *T*_m_ and 3′ unpaired nucleotide.

## Results

### Loss of XRN1 reduces the retrotransposition efficiency of human L1s

Recent evidence supports the crucial role of *L1* mRNA 3′ end. Specifically, *L1* requires poly(A) tail for retrotransposition (32), and we demonstrated that uridylation of *L1* mRNA 3′ ends by TUT4 and TUT7 terminaluridyltransferases reduces L1 retrotransposition by 20 to over 90% depending on the length of the uridine tail (33). We hypothesized that other general RNA metabolism factors involved with RNA 5′ and 3′ ends will significantly affect L1 retrotransposition and thus its biology. We first concentrated on the 5′→3′ exoribonuclease XRN1 and hypothesized that knocking it down could increase *L1* mRNA levels and retrotransposition.

To test the effects of XRN1 we used a well-established L1 retrotransposition reporter assay in 293T cells (47,71,72). The assay relies on a plasmid encoding a full-length retrotransposition competent (*RC*) *L1* appended with a reporter cassette and a strong polyadenylation signal. The functional reporter can only be produced following a successful retrotransposition event (Figure 1A). We co-transfected 293T cells with the *L1 egfpI* reporter plasmids producing EGFP following successful retrotransposition and either of 3 different siRNA duplexes targeting *XRN1* or control non-targeting siRNAs (*N-t*). The efficient depletion of XRN1 protein to ca. 12–25% of the *N-t* control levels was confirmed by western blot (Figure 1B). At day 5 post-transfection we analysed the cells by flow cytometry. Curiously, we observed a significant reduction of L1 retrotransposition by roughly 3-fold upon *XRN1* depletion by RNAi with all of the 3 different siRNAs tested (Figure 1C). To substantiate our results, we created *XRN1* knock-out (KO) cell lines in 293T background using CRISPR-Cas9 genome engineering. We successfully generated 6 independent clonal *XRN1* knock-out cell lines and selected 6 control cell lines expressing XRN1 which included the parental 293T and 5 independent clones that retained wild-type *XRN1* alleles producing full-length protein as validated by western blot and sequencing of genomic DNA (Figure 1D, Supplementary Figure 1B,C). We observed severe reduction in L1 retrotransposition in all the *XRN1* KOs (Figure 1E, F, Supplementary Figure 1D,E, Supplementary File). The levels of retrotransposition in the different KO clonal cell lines were reduced from 2-fold to 70-fold with a median of 12-fold for all possible combinations (Supplementary Figure 1F). Importantly, the effect was bigger than following the *XRN1* knock-down by RNAi, confirming that XRN1 dose affects L1 retrotransposition. Furthermore, all the cell lines transfected at very similar levels with plasmids encoding either *EGFP* or *mCherry* as evidenced by a similar percentages of transfected cells and levels of expression of these proteins in the cells (Supplementary Figure 2A–D). To further test whether the observed reduction of L1 retrotransposition might result from a more general effect of XRN1 depletion onto cellular metabolism we measured cell cycle and levels of apoptotic cells in the total cell populations of the *XRN1* KO and control cells. We observed subtle changes to the cell cycle with higher percentage of the *XRN1* KO cells in the G_0_/G_1_ phases and simultaneously a lower percentage of these cells in the S phase (Supplementary Figure 2E). Also there were more apoptotic cells in the *XRN1* knock-outs (Supplementary Figure 2F). L1 retrotransposition-positive cells accumulated in both wild-types and *XRN1* KOs when cells were cultivated for longer periods of time, with the fastest rates for cell lines supporting the highest levels of retrotransposition (Supplementary Figure 2G, H). To test whether the observed effect on L1 retrotransposition might be a peculiarity of 293T cells we attempted generating *XRN1* KO in other backgrounds and succeeded with HeLa HA cells (Supplementary Figure 2I–L). We performed the L1 retrotransposition assay in the HeLa HA wild-type and *XRN1* KO cells using a reporter, L1.3 *mneoI*, that upon retrotransposition confers resistance to G418 antibiotic. We observed a reduced number of G418-resistant colonies in the *XRN1* KO (compare wells of the L1.3 *mneoI* plasmid transfected and pT2 *neo* plasmid transfected cells in panels for the wild-type and for the KO cells; Supplementary Figure 2M). Also the KO cells grew worse than the wild-types (Supplementary Figure 2L). Since the expression of the L1 reporter in the above assays relied on the endogenous *L1* promoter, we decided to test whether the *L1* promoter is required for the observed effect. To this end we used different *L1 egfpI* reporters in which the 5′ UTR *L1* promoter was replaced with human cytomegalovirus (CMV) promoter (33). Furthermore, the two reporter plasmids possessed 3′ UTR and polyadenylation signal (pAS) of HSV (as opposed to SV40 pAS; Figure 1G, Supplementary Figure 2N). L1 retrotransposition was reduced in the *XRN1* KO cells (B9) irrespective of the reporter used (Figure 1G). However, the L1 retrotransposition levels were different between the reporters likely reflecting the higher expression of L1 reporter from the CMV promoter (reporter A) and the efficiency-reducing effect of the mCherry tag on L1-ORF1p (reporter B; Figure 1G). Finally, to confirm our observations we obtained *XRN1* KO 293T cells generated independently in another lab and using different guides for genome editing (61). We also obtained *DCP2*, and a double *DCP2* and *XRN1* KO (61). We confirmed the lack of expression of XRN1 and DCP2 proteins in the respective cell lines by western blotting (Figure 1H), and analysed their cell cycle, and the levels of apoptosis (Supplementary Figure 2O,P). The *DCP2* and the double *DCP2* and *XRN1* KOs were enriched in G2/M phase as compared to the wild-type and *XRN1* KO cells (Supplementary Figure 2O). The L1 retrotransposition assay in these cells revealed reduction of L1 mobility in all the KO cell lines (Figure 1I) irrespective of the presence of 5′ UTR promoter, 3′ UTR, and pAS (Figure 1J). The reduction of L1 mobility was the smallest in the *DCP2* KO (Figure 1I,J).

Taken together, we demonstrated that reduction of XRN1 dose reduced L1 retrotransposition. Milder reduction of L1 retrotransposition was also observed with the *DCP2* knock-out cells.

### XRN1 rescue restores L1 retrotransposition

To test whether the observed reduction in L1 retrotransposition was due to the lack of XRN1, we cloned wild-type *XRN1* gene into pKK_no_tag plasmid (64) to express the protein without any tag. First, we performed a L1 retrotransposition experiment without or with *XRN1* rescue in all the six *XRN1* 293T KO cell lines. The cells were transfected with the L1 *egfpI* retrotransposition reporter and either control plasmid or the *XRN1* encoding plasmid. The expression of full-length XRN1 was confirmed (Figure 2A), leading to a significant increase of L1 retrotransposition in all of the tested cell lines by a median of ∼5.7 (Figure 2B, C, Supplementary File). Thus ectopic expression of wild-type *XRN1* in the *XRN1* KO cells restored L1 retrotransposition nearly to the levels observed with the wild-type cells. We then addressed the question whether the XRN1 catalytic 5′→3′ exoribonuclease activity is required for the restoration of L1 retrotransposition. To this end we mutated the *XRN1* gene and encoded the mutated copies on plasmids. As with the wild-type *XRN1* gene no tags were appended. The mutants within the *XRN1* catalytic exoribonuclease domain were either double, with two residues mutated: (i) R100A and A101G (mutant 1), or (ii) D206N and D208N (mutant 2), or (iii) a quadruple mutant comprising all these mutations (mutant 3). All mutated proteins are expected to have compromised exoribonuclease activity, with the quadruple mutant most affected (73). We delivered the L1 *egfpI* retrotransposition reporter plasmid together with increasing amounts of plasmids comprising either wild-type or the mutated *XRN1* genes into the *XRN1* knock-out cells (cell line B9) and performed the L1 retrotransposition assay. The appearance of the wild-type or mutant XRN1 proteins and their dependence on the amounts of transfected plasmids were confirmed by western blots (Figure 2D,E). As expected, the wild-type XRN1 effectively restored L1 retrotransposition in a dose-dependent manner (Figure 2F). Neither of the XRN1 variants mutated within the exoribonuclease domain could effectively restore L1 retrotransposition (Figure 2F). We observed some rescue with the mutant 1. This likely retains some exoribonuclease activity, but its ability to continuously remove nucleotides without dissociation, the so called processivity, and its discrimination against capped RNAs is compromised (73).

In sum, we confirmed that XRN1 exoribonuclease activity is important to restore L1 retrotransposition in the *XRN1* deficient cells.

### *L1* mRNA is stabilised but its translation is poorer in the *XRN1* knock-out cells

**Figure 3.**
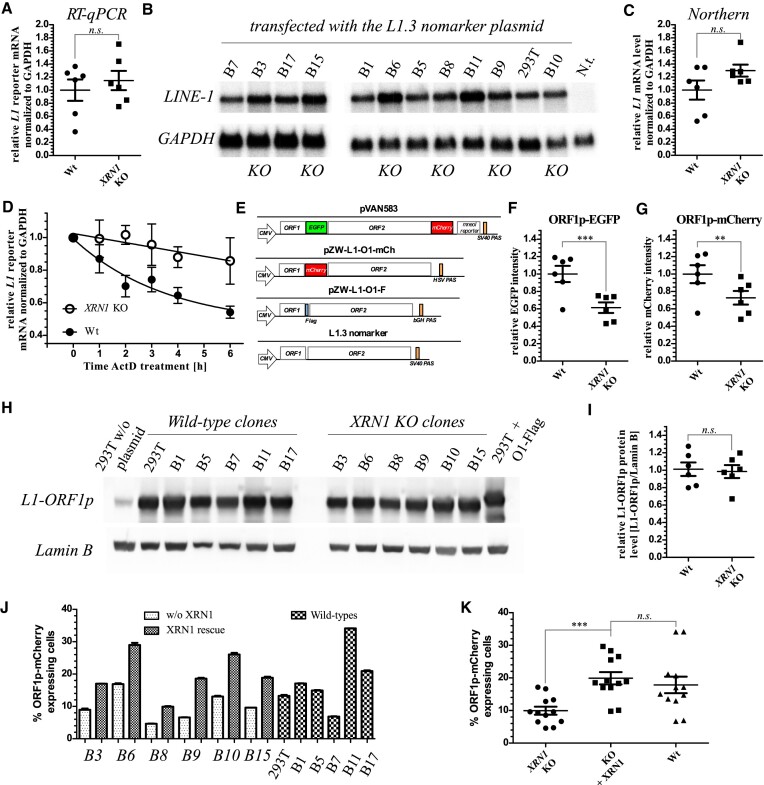
The effects of *XRN1* knock-out on*L1* RNA and proteins. (**A**) Estimation of the levels of *L1* reporter mRNA in six wild-type and six *XRN1* KO clonal 293T cell lines by RT-qPCR. Data are shown as mean ± SEM. Lack of statistically significant difference between the two groups was calculated by unpaired *t*-test. (**B**) Northern blot of *L1* mRNA ectopically expressed from a plasmid and lacking the reporter cassette. Twelve different cell lines were analysed side-by-side as indicated. A non-transfected control (N.t.) is shown. *GAPDH* is the loading control. (**C**) A quantitation of the northern blots of panel B. Data are shown as mean ± SEM. Lack of statistically significant difference between the two groups was calculated by unpaired *t*-test. (**D**) A time course assay following treatment of the cells with actinomycin D (ActD) to stop transcription by RNA polymerase II. Levels of *L1* reporter mRNA were measured relative to *GAPDH* in the wild-type and *XRN1* KO cells (three independent cell lines for each group) in the indicated time points before (0) and after ActD addition. Data are shown as mean ± SEM. (**E**) A graphical representation of the constructs expressing (tagged) L1 proteins used in the following studies. (**F**) Relative median ORF1p-EGFP intensity (mean EGFP intensity in the EGFP expressing wild-type cells normalized to 1.0 in the six wild-type and six *XRN1* KO clonal cell lines. Each point represents a mean of three replicates ± SEM. Statistical significance between the two groups was calculated by unpaired *t*-test. (**G**) As in panel F but using a L1-ORF1-mCherry reporter. (**H**) Western blot of untagged L1-ORF1p expressed from L1 reporter plasmid and Lamin B (loading control) in the different cell lines as indicated. Lane 1 (left) – 293T without transfection of the reporter, lane 14 (right) – 293T following transfection with L1-ORF1p-Flag expressing reporter. (**I**) Quantitation of the blots in panel H. (**J**) An effect of ectopic expression of the wild-type non-tagged XRN1 in the *XRN1* KO cells on ORF1p-mCherry expression from the reporter plasmid. (**K**) Data from panel J presented as mean ± SEM to demonstrate statistically significant change in the per cents of ORF1p-mCherry expressing cells between the KO and KO plus XRN1 conditions as calculated by unpaired t-test.

To elucidate what could be the molecular reasons for reduced L1 retrotransposition in the *XRN1* KO cells we systematically tested the steady-state levels of *L1* RNA and proteins in the *XRN1* KO and the wild-type cells. To investigate steady-state levels of *L1* reporter mRNA we used two independent approaches: reverse transcription and quantitative PCR (RT-qPCR), and Northern blot. We first measured the amounts of *L1 egfpI* reporter used in the retrotransposition assays. The *L1 egfpI* reporter comprises two parts of *EGFP* gene interrupted with an efficiently spliced γ-globin intron. The primers used in the qPCR hybridize within exonic sequences and flank the intron, while the TaqMan probe binds to the spliced sequence (Supplementary Figure 3A, B, Supplementary Table 1). This arrangement ensures binding of the probe only to the spliced *L1* reporter and not to the L1 reporter plasmid (co-purifying with total RNA), or endogenous *L1* sequences that lack the reporter cassette. *GAPDH* mRNA levels were measured in the same multiplexed reactions, and used to normalize *L1* reporter mRNA. Despite the reduction of L1 retrotransposition in the *XRN1* KO cells, the steady-state levels of *L1 egfpI* reporter mRNA in these cells were slightly higher than in the wild-type cells with an estimated mean level of 1.15-fold of the wild-type level (1.00), but without reaching statistical significance (Figure 3A). The amounts of transfected plasmid isolated from the cells were similar (Supplementary Figure 3C, D). To further test the amounts and qualities of *L1* mRNA in the cells we transfected them with a different *L1* plasmid, JM101/L1.3 nomarker (18), that encodes full-length retrotransposition-competent L1 but without any reporter cassette and produces enough *L1* reporter mRNA to visualize it in a Northern blot without enrichment for poly(A) or capped mRNAs. This was important as we speculated that the *L1* 3′ polyadenylation or 5′ capping could be affected by XRN1 deficiency. We observed full-length *L1* mRNA following the transfection but not with total RNA from non-transfected cells, confirming efficient expression of *L1* from the plasmid above (>200-fold) the endogenous expression levels (Figure 3B, Supplementary Figure 3E). Importantly, we consistently observed an upregulation of *L1* mRNA when normalized to *GAPDH* mRNA by an average of 1.3-fold in the *XRN1* KO cells as compared to the wild-type cells (Figure 3C). We hypothesized that the reason for the slightly higher *L1* mRNA levels in the KO cells could be its increased stability due to the lack of XRN1. To explore this possibility we performed a time-course experiment following actinomycin D-induced transcriptional shut-down. We observed stabilisation of *L1 egfpI* reporter mRNA in the *XRN1* KO (Figure 3D), that explains the slight difference in the steady-state levels and agrees with the hypothesis that *L1* reporter mRNA is a target of XRN1 exoribonucleolytic activity.

To assess the levels of translation of L1 proteins we transfected the six wild-type and six *XRN1* KO cell lines with plasmids encoding full-length RC *L1* with *L1-ORF1* tagged with either *EGFP*, *mCherry*, *FLAG* or without any tags (JM101/L1.3 nomarker) (Figure 3E), and after 48 h analysed the median EGFP or mCherry intensities using flow cytometry or performed western blots. We observed a decrease of roughly 40% in the median intensities of L1-ORF1p tagged with either of the fluorescent protein (FP) tags, or Flag (Figure 3F, G, Supplementary Figure 3F-J), but not with the untagged L1-ORF1p (Figure 3H, I). We confirmed the expression of the fusion proteins comprising L1-ORF1p and either of the FP-tags (Supplementary Figure 3F, G, I). It is currently unknown why tagged L1-ORF1p was expressed worse than the untagged protein but it might explain lower retrotransposition with the tagged reporters e.g. Figure 1G. To test whether XRN1 expression could restore L1 protein production we ectopically expressed it from a plasmid in the *XRN1* KO cells. Indeed, expression of XRN1 in these cells restored expression of the tagged L1-ORF1p protein to the levels observed in the wild-type cells (Figure 3J, K). To test whether the lowered expression of the L1 proteins could result from changes in *L1* reporter mRNA 3′ ends we performed an RNase H experiment with a set of DNA oligonucleotides hybridizing to L1 transcripts with and without oligo(dT)_15_ DNA. Most non-templated 3′ tails on *L1* reporter mRNA fell within a length of 0–76 nucleotides with some tails extending up to <160 nucleotides (Supplementary Figure 3K–M). When *L1* reporter mRNAs from the wild-type and *XRN1* KO cells were compared, the latter were shorter by ∼15 nucleotides (in the range from 0 to 76 nucleotides; Supplementary Figure 3L,M). Because the resolution of the method was poor we used a high-throughput method in later experiments.

Taken together we demonstrated that XRN1 deficiency stabilised *L1* mRNA slightly increasing its steady-state levels. In spite of this steady-state levels of L1-ORF1 protein was reduced, but could be restored by ectopic XRN1 expression.

### XRN1 loss reduces L1 body formation but does not affect L1-ORF2p activities

**Figure 4.**
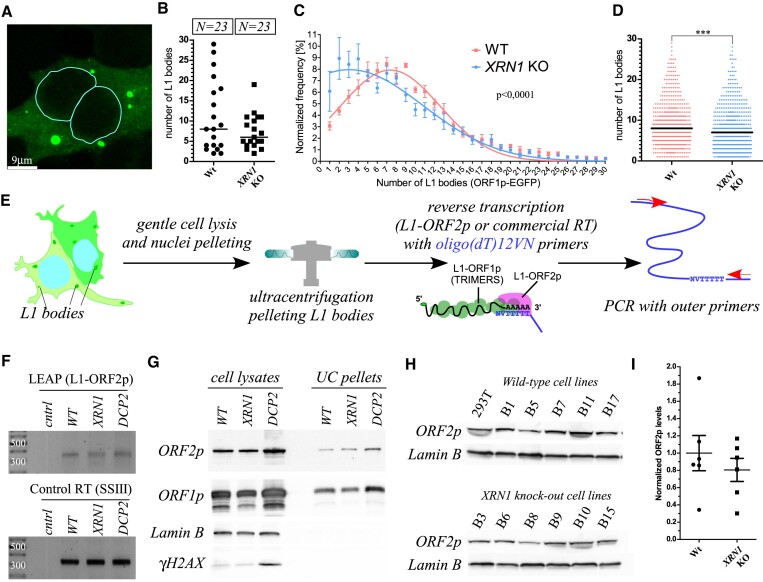
The effects of *XRN1* knock-out on L1 cytoplasmic bodies and on L1-ORF2p. (**A**) Confocal microscopy z-stacks of 293T wild-type cells transfected with the L1-ORF1-EGFP expressing construct. L1-ORF1p-EGFP is coloured green. Visible are EGFP-enriched L1 bodies. Nuclei were stained with Hoechst and are marked by a cyan contour. White bar represents 9 μm. (**B**) Confocal microscopy z-stacks were analysed visually and the number of L1 bodies were quantified in 23 wild-type and 23 *XRN1* KO cells. Median number of L1 bodies is shown as a line. (**C**) Result of an automated imaging flow cytometry analysis. All six wild-type and six *XRN1* KO cell lines were transfected with the L1-ORF1-EGFP construct and the numbers of L1 bodies were calculated automatically. Normalized frequency adds up to 100%. Data are shown as mean ± SEM. The two fits were made and their statistically significant difference was estimated by *F*-test using Prism software. (**D**) Numbers of L1 bodies detected in the analysis in panel C. Median numbers are shown as black lines. Statistical significance between the two sets was calculated by a non-parametric Mann-Whitney test. (**E**) A graphical representation of the workflow of the L1 amplification protocol (LEAP). Following transfection of the cells with L1 reporter the L1 bodies are pelleted by ultracentrifugation and combined with anchoring oligodT (dT_12_VN) primers to initiate reverse transcription by L1-ORF2p. PCR with outer primers is used to amplify L1 fragments. (**F**) Results of the LEAP assay using wild-type (WT), *XRN1* KO and *DCP2* KO 293T cells as indicated. Control RT was performed by addition of a commercial reverse transcriptase. Control condition refers to blank ultracentrifugation (without cell lysate). Numbers on ladder bands indicate their DNA lengths. (**G**) Western blot to detect L1-ORF2p, L1-ORF1p, Lamin B and γH2AX in the cleared lysates and ultracentrifuged material (UC pellets) from the LEAP experiment presented in panel F. (**H**) Western blot analysis of cell lysates from six wild-type and six *XRN1* KO cell lines following transfection with the JM101/L1.3 nomarker. Lamin B is loading control. (**I**) A quantitation of the ORF2p signal in panel G normalized to lamin B signal and with mean of the wild-types set to 1.00. Data are shown as mean ± SEM.

L1 is known to form RNPs that accumulate in cytoplasmic L1 bodies. To test whether XRN1 deficiency affects formation of L1 bodies we obtained confocal microscopy pictures of either the wild-type or *XRN1* KO cells expressing L1-ORF1p tagged with EGFP. In both types of cells L1 bodies could be observed (Figure 4A and Supplementary Figure 4A,B). A slightly lower number of L1 bodies was observed in the *XRN1* KO cells (Figure 4B). To validate these observations in an unbiased way we performed analytical flow cytometry with imaging of individual cells and automated analysis for all the six wild-type and six *XRN1* KO cell lines used in this study transfected with the L1-ORF1p-EGFP expressing *L1* reporter plasmid. Thousands of cells for each cell line were photographed to reveal median numbers of eight and seven L1 bodies per cell in the wild-type and *XRN1* KO cells respectively (Figure 4C, D, Supplementary Figure 4C). Accordingly, the median total area of L1 bodies was smaller in the *XRN1* KO cells (Supplementary Figure 4D). The automated analysis was also performed on cells transfected with the L1-ORF1-mCherry reporter plasmid validating these observations (Supplementary Figure 4E, F).

L1-ORF2p activity is crucial for retrotransposition. To test the L1-ORF2p reverse transcriptase activity we performed the L1 element amplification protocol (LEAP) (69). The assay relies on pelleting L1 bodies by ultracentrifugation and performing reverse transcription using the pelleted L1-ORF2p in the presence of an added primer, followed by PCR (Figure 4E). We performed LEAP on materials from the wild-type and *XRN1* KO cells transfected with the JM101/L1.3 nomarker reporter. Additionally, we performed LEAP on materials from the *DCP2*, and the double DCP2 and XRN1 knock-out cell lines (61). L1-ORF2p produced *L1* cDNA in all wild-type, *XRN1* and *DCP2* KO samples in three independent experiments (Figure 4F and Supplementary Figure 4G). Positive controls performed with a commercial reverse transcriptase added to the pelleted L1 bodies produced expected PCR products validating the presence of *L1* mRNA in these pellets (Figure 4F). To estimate the amounts of L1-ORF2p in the pelleted materials we performed western blot and probing for L1-ORF2p using a rabbit monoclonal antibody. We additionally probed for L1-ORF1p, and Lamin B, which was a loading control for the lysates. We observed L1-ORF2p and L1-ORF1p, but not lamin B in the pelleted materials suggesting that the pellets contain L1 bodies free of nuclear contamination (Figure 4G, Supplementary Figure 4H,I). Surprisingly, we observed that the L1-ORF2p antibody could detect L1-ORF2p expressed from the JM101/L1.3 nomarker plasmid also in the whole cell lysates (Figure 4G). This was unexpected as L1-ORF2p usually escapes detection due to its very low concentrations in cells and expression in only a subset of cells in a population (26,68). When compared to the wild-type cells L1-ORF2p was less abundant in the *XRN1* KO, but more abundant in the *DCP2* KO cells (Figure 4G, Supplementary Figure 4H,J,K). This observation was further validated for the 6 independent wild-type and *XRN1* KO cell lines (Figure 4H,I), in which L1-ORF2p was reduced to ∼80% of the wild-type levels. In the *DCP2* KO cells and the double *DCP2* and *XRN1* KO cells the amounts of L1-ORF2p were ∼2,1-fold higher than in the *XRN1* KO cells (Figure 4G and Supplementary Figure 4K).

Finally, we probed the blots for γH2AX, a phosphorylated form of the H2AX histone, that is formed in response to double strand breaks (DSB) in genomic DNA. Since DSB can be induced by overexpression of L1-ORF2p and its endonuclease activity, we assumed we could quantitate the γH2AX as an approximation of L1-ORF2p endonuclease. Indeed, there were higher γH2AX levels in the *DCP2* KO cells expressing the highest L1-ORF2p levels (Figure 4G). We observed lower amounts of γH2AX staining in the six clonal *XRN1* KO cell lines as compared to their wild-type controls following transfection with JM101/L1.3 nomarker plasmid (Supplementary Figure 4L, M). Despite the increased levels of L1-ORF2p by using a high-throughput flow cytometry with single cell imaging we showed a reduced number of L1 bodies in the *XRN1*, *DCP2*, and the double *XRN1* and *DCP2* KO cells with a median number of 7 L1 bodies in these KO cells as compared to 8 in the wild-type cells (Supplementary Figure 4N–P).

Summarizing we observed formation of the L1 bodies in all tested cells. When compared to the wild-type cells in all the KO cell lines smaller numbers and area of L1 bodies were observed. Despite this, we observed higher steady-state levels of L1-ORF1p and L1-ORF2p proteins in the *DCP2* and the double *DCP2* and *XRN1* KO cells. We further either directly or indirectly confirmed the presence of the L1-ORF2p reverse transcriptase and endonuclease activities in all cell lines tested.

### Uridylated *L1* mRNAs accumulate in the *XRN1* and *DCP2* knock-out cells

**Figure 5.**
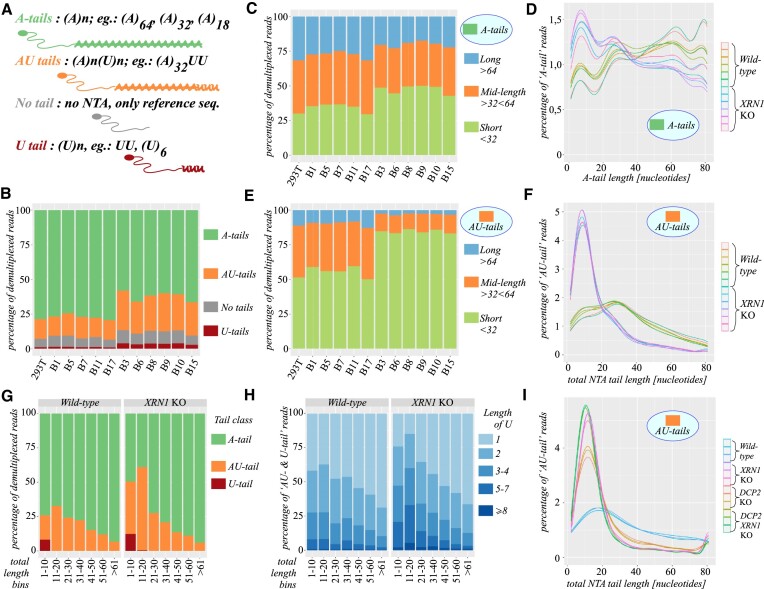
High-throughput 3′ RACE-seq of *L1* reporter mRNA 3′ ends. (**A**) A graphical representation of the four different non-templated 3′ ends. The color-coding is preserved in panels B and G. Examples of different 3′ tail classes are provided in text and graphics. (**B**) Distribution of A-tails, AU-tails, no tails, and U-tails in the 3′ RACE-seq data on *L1* reporter mRNA in the different clonal cell lines. (**C**) Distribution of lengths of A-tails in the indicated cell lines. Three length classes are color-coded: long tails > 64 A nucleotides in blue, mid-length (32–64 As) in orange, and short tails (<32 As) in green. (**D**) Length distribution of the A-tails on *L1* reporter mRNA in the range from 1 to ≥ 81 As in all analysed cell lines as indicated. (**E**) As in panel C but lengths of AU-tails are shown. (**F**) As in panel D but lengths of AU-tails are shown. (**G**) Distribution of A-tails, AU-tails, and U-tails related to the total lengths of the tails divided into 10 nucleotide bins. Accumulated data for all wild-type and all *XRN1* KO cell lines is shown. 100 equals all A-tails, AU-tails, and U-tails. (**H**) Distribution of lengths of U tails in AU-tails and U-tails classes. Lengths of U-tails are related to the total lengths of the tails and divided in 10-nucleotide bins. (**I**) Distribution of AU-tails’ lengths in the independently derived control and *XRN1* KO cells, and in *DCP2*, and the double *DCP2* plus *XRN1* KO cell lines as indicated.

We hypothesized that the observed reduction in L1 retrotransposition in the *XRN1* KO cells could result from increased uridylation of *L1* mRNA 3′ ends since uridylated *L1* mRNAs could not be cleared by XRN1 (33,74). To study the dynamics of *L1* mRNA 3′ ends we prepared libraries of JM101/L1.3 nomarker reporter mRNA and 2 control endogenous mRNAs’: *GAPDH* and *PABPC4*, from the 293T *XRN1* KO and wild-type cells, for high-throughput sequencing by Illumina Novaseq. *PABPC4* was found heavily uridylated, and *GAPDH* was found scarcely uridylated in earlier studies (33,74,75). Importantly, we used preadenylated 3′ adapters to include in the analysis all 3′ non-templated additions (NTA). The adapters comprised a degenerate 15-nucleotide sequence, unique molecular identifiers (UMI), that allowed correcting for PCR artefacts during analysis (Supplementary Figure 5A). The NTAs are nucleotides that reside in the 3′ parts of the sequenced base-paired reads and do not map to the reference genomic or plasmid sequences. We classified the NTAs into four groups: A-tails—comprising only a variable number of adenines, AU-tails—comprising adenine tails appended with a single or multiple U residues, U-tails—comprising only non-templated uridines, and no tails—comprising 3′ ends lacking any NTA (Figure 5A). The analysis revealed uridylation of 17% of the *L1 reporter* mRNA in the wild-type cells and this fraction increased to 32% in the *XRN1* KO cells (Figure 5B). Most uridylation was observed on poly(A) and oligo(A)-tailed *L1* reporter mRNAs (AU-tails) that constituted 15% in the wild-type cells and 27% in the *XRN1* KO cells (Figure 5B). We also observed that 6–7% of *L1* reporters were deprived of any tails, likely representing transcripts in the process of degradation (Figure 5B). All of the observed differences were highly statistically significant (Supplementary Figure 5B). By using paired-end reads we estimated the lengths of NTA tails within a size window of 0–80 nucleotides. This was because lengths of poly(A) tails and non-templated tails in the short-read Illumina sequencing cannot be longer than the read lengths themselves (excluding part of the adapter used for demultiplexing, UMI, and the delimiter used to separate the UMI from the rest of the read; Supplementary Figure 5A, Supplementary Methods and Supplementary Note 2). Nevertheless, despite this limitation this modified 3′ RACE-seq protocol is currently the best solution for sequencing of 3′ NTAs in terms of throughput and quality of NTA identification. In this approach R2 reads are used to tell 3′ terminal modification and it is the first sequenced base following the delimiter sequence (Supplementary Figure 5A). Both R1 and R2 reads are then used to estimate the 3′ NTA tails’ lengths. We divided the A-tails into 3 length classes: 1–32 nucleotides, 33–64 nucleotides, and longer than 64 nucleotides and observed a statistically significant shortening of the A-tails in the *XRN1* KO cells (Figure 5C, Supplementary Figure 5D). Within the 0–80 tail length window the median length of the A-tails dropped from 47 As in the wild-type to 35 As in the *XRN1* KO cells (Figure 5D, Supplementary Figure 5C). The AU-tails encompassed mostly short (1–32) and mid-length tails (33–64) (Figure 5E). We also detected uridylation of even long poly(A) tails (Figure 5E–G). The most striking observation was a significant shortening of the AU-tails from the median length (including both A and U) of 30 nucleotides in the wild-type to the median of 12 in the *XRN1* KO cells (Figure 5F, Supplementary Figure 5E, F). The number of uridines in the AU-tails and in the U-tails raised significantly in the *XRN1* KO (Figure 5H). Analyses of the control *GAPDH* and *PABPC4* revealed much smaller changes (Supplementary Figure 5G–L). To further substantiate our observations we performed the 3′ RACE-seq analyses on RNA retrieved from the *XRN1*, *DCP2*, *DCP2* plus *XRN1* KO cell lines (61). Three technical replicates for each cell line were prepared. The results obtained for the independently raised KO cell lines and their control matched those obtained with the *XRN1* KO cell lines created in house. Especially the striking shortening of the AU-tails of the *L1* reporter mRNA was conserved (Figure 5I).

Taken together we demonstrated that in the *XRN1, DCP2*, and *DCP2* plus *XRN1* KO cells *L1* reporter mRNAs 3′ ends are deadenylated and heavily uridylated.

### Step-wise activities of 5′ and 3′ factors on *L1* shape its retrotransposition potential

**Figure 6.**
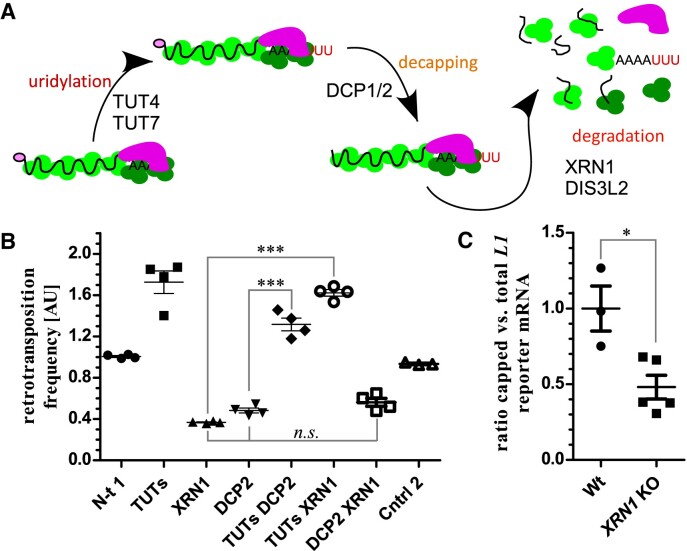
Order of events on 3′ and 5′ mRNA ends and its impact on L1 retrotransposition. (**A**) A graphical representation of the assumed order of events occurring on (*L1*) mRNA 3′ and 5′ ends. Processes and proteins involved in each of the stages are indicated. (**B**) L1 retrotransposition assay in 293T cells following temporal depletion of the indicated factors by RNAi. Control 2 (Cntrl 2) is siRNA targeting TUT1 that does not affect retrotransposition (33). Data are shown as mean ± SEM. Statistical significance was calculated by ANOVA followed by Tukey's test. (**C**) RT-qPCR before and following selection of capped mRNAs by GST-eIF4E mutant protein. Each point is a calculation made for a single wild-type or *XRN1* KO cell line. Data are shown as mean ± SEM. Statistical significance was calculated by unpaired t-test.

The effects observed on L1 retrotransposition in XRN1 deficiency likely depend on other factors. These include TUT4 and TUT7 enzymes that uridylate *L1* mRNAs (33), and DCP2 that in complex with DCP1 removes 5′ cap off mRNAs presenting them as targets for XRN1 exoribonucleolysis (Figure 6A). Furthermore, uridylation was shown to precede and stimulate decapping (54,55). To test whether the expected order of events is conserved in the phenotypes on L1 retrotransposition we performed L1 retrotransposition assays in 293T cells depleted of the factors in different combinations by using siRNA (Supplementary Figure 6A,B). As expected knock-down of both TUTases led to a noticeable increase of L1 retrotransposition (Figure 6B). On the other hand the individual knock-downs of XRN1 or DCP2 reduced L1 retrotransposition (Figure 6B, see also Figure 1H–J). Importantly, simultaneous knock-down of TUTases and either XRN1 or DCP2 resulted in retrotransposition levels very similar to just TUTases’ knock-down and definitely higher than in individual XRN1 or DCP2 knock-downs (Figure 6B). This supports the point that the activity of TUTases is needed for the effects observed in *XRN1* and *DCP2* KO conditions. Simultaneous knock-down of DCP2 and XRN1 resulted in an expected reduction of L1 retrotransposition (Figure 6B). We expected that *L1* mRNA accumulating in the *XRN1* KO cells might lack the protective 5′ cap structure removed by the DCP1/2 complex prior to the XRN1-mediated 5′→3′ exoribonucleolysis. To test this possibility we affinity purified capped RNAs from pools of total RNA from wild-type and *XRN1* KO clonal cell lines. This was achieved by using a recombinant eIF4E K119A mutant protein fused with GST tag and selection on glutathione agarose (76) (Supplementary Figure 6C–E). To estimate the amounts of *L1 egfpI* reporter mRNA, and endogenous *GAPDH* mRNA we used RT-qPCR and normalized the data to *MALAT* lncRNA levels, as the latter is a nuclear RNA and thus likely not an XRN1 target. We observed a reduction by a factor of ∼2 in the amounts of capped *L1* reporter mRNA (Figure 6C). Finally, we tested the effects of DIS3L2 3′→5′ exoribonuclease that preferentially degrades uridylated substrates (77), and works independently of the 3′→5′ RNA exosome complex (78). Expression of a dominant negative mutant of DIS3L2 (D391N) was shown to increase the fraction of uridylated RNAs (79). We performed L1 retrotransposition assay in 293T cells co-transfected with the L1 reporter and plasmids to overexpress either wild-type or the dominant-negative D391N DIS3L2 mutant proteins (Supplementary Figure 6F). A control with a similarly sized plasmid encoding maltose binding protein (MBP) was used. Expression of the DIS3L2 dominant mutant significantly reduced L1 retrotransposition as compared to the control and cells overexpressing wild-type DIS3L2 (Supplementary Figure 6F,G). In fact, in the cells overexpressing the wild-type DIS3L2 L1 retrotransposition was slightly increased as compared to the control, possibly due to trimming of oligo(U) (Supplementary Figure 6G).

To sum up we confirmed step-wise activities of different post-transcriptional factors on *L1* mRNA and retrotransposition.

### *XRN1* and *DCP2* depletions stabilize oligoadenylated, uridylated endogenous *L1*s

**Figure 7.**
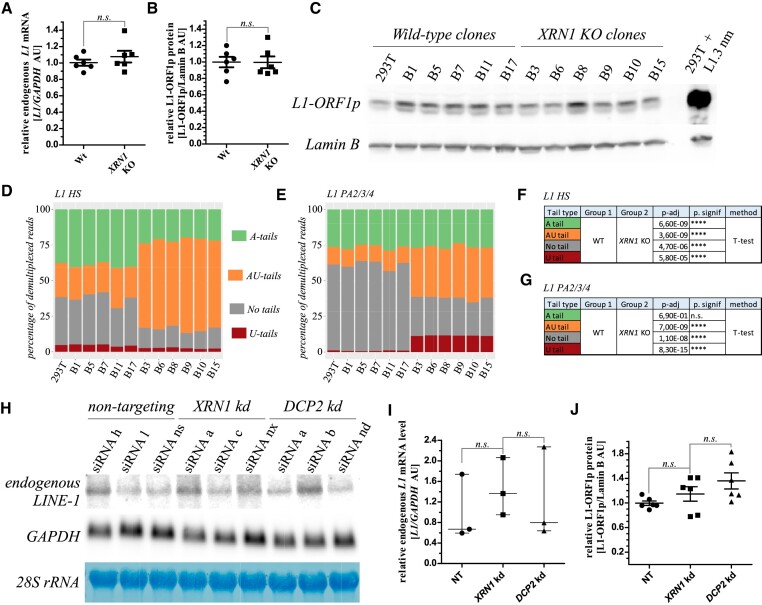
The effects of *XRN1*and *DCP2*depletion on endogenous *L1*. (**A**) Relative estimation of endogenous *L1* mRNA in the 293T wild-type and *XRN1* KO cells by RT-qPCR. The lack of statistical significance was calculated by an unpaired t-test. (**B**) As in panel A but relative estimation of the endogenously expressed L1-ORF1p protein on the basis of a western blot quantitation is shown. (**C**) Western blot of the endogenous L1-ORF1p and Lamin B (loading control) used to make calculations for panel B. Right lane shows overexpression of L1 from the reporter plasmid for comparison. (**D**) Results of 3′ RACE-seq analysis of endogenous *L1*-*HS* in 293T clonal cell lines as indicated. Percentages of different tails’ types are shown and color-coded as indicated. (**E**) Same as in panel D but results for endogenous *L1-PA2/3/4* are shown. (**F**) Statistical analysis of the changes observed for *L1*-*HS*. Related to panel D. (**G**) Statistical analysis of the changes observed for *L1-PA2/3/4*. Related to panel E. (**H**) Northern blot of endogenous *L1*, *GAPDH*, and 28S rRNA (methylene blue stained) in PA-1 cells following temporal (48 h) depletion of *XRN1*, and *DCP2* by RNAi as indicated. (**I**) Quantitation of the blot in panel I. Lack of statistical significance was calculated by unpaired t-test. (**J**) Quantitation of endogenously expressed L1-ORF1p levels in PA-1 following temporal (48h) depletion of *XRN1*, or *DCP2* by RNAi as indicated. Statistical analysis performed by unpaired t-test.

The 3′ UTRs of endogenous *L1* are different from the reporters. We set out to explore the effects of XRN1 on endogenous *L1*s, and observed minor changes in the steady state levels of *L1* mRNA and L1-ORF1p protein in the clonal wild-type and *XRN1* KO 293T cells (Figure 7A–C). To explore the dynamics of endogenous *L1* mRNA 3′ ends we performed 3′ RACE-seq and analysed the data as to differentiate between retrotransposition competent *L1-HS* mRNA and older *L1*classes*(L1*-*PA2-5)* (80) (Supplementary Figure 7A). An increase in endogenous *L1* uridylation and decrease in the fraction of (poly)adenylated *L1*-*HS* was observed in *XRN1* KO. In the case of older *L1s*, there was an increase in uridylation but without a significant change in the fractions of polyadenylated 3′ ends (Figure 7D-G). We noticed higher uridylation levels with the evolutionarily young *L1*-HS, than with the older *L1* classes (Figure 7D–G). This was accompanied by differentiating effects on *L1* non-templated ends’ lengths (Supplementary Figure 7B, C). The high levels of ‘no tails’ seen in Figures 7D, E could either represent true degradation intermediates or be an result of preferential amplification of such reads during PCR in library preparation. We failed in generating stable genomic knock-outs of *XRN1* in PA-1 cells that express high levels of endogenous *L1*. In these cells temporal knock-down of *XRN1*, *DCP2*, and *DIS3L2* was obtained by RNAi (cells were harvested 48h after siRNA transfection). We observed stabilization of endogenously expressed *L1* mRNA and an increase of L1-ORF1p upon *XRN1* and *DCP2* knock-down conditions (Figure 7H–J). However, unlike in 293T uridylation of *L1* 3′ ends did not increase (Supplementary Figure 7D–F). The effect could reflect differences between endogenous *L1* in 293T and PA-1. However, we hypothesized that no increase in uridylation could be due to short time of RNAi. We thus depleted *XRN1* and *DCP2* by two consecutive siRNA transfections over 126 h. Longer depletion resulted in a clear increase of uridylated endogenous *L1* and a drop in the fractions of adenylated *L1* (Supplementary Figure 7G).

We confirmed increased uridylation of endogenous *L1* in *XRN1* and *DCP2* depletion conditions in 293T and PA-1 cells.

## Discussion

In this work we produced genomic gene knock-out cell lines and performed temporal RNAi-mediated depletion to eliminate or reduce expression of *XRN1* and *DCP2*, and used these as tools to demonstrate the importance of *L1* 3′ non-templated ends’ dynamics to L1 biology and retrotransposition. We demonstrated reduced L1 retrotransposition in *XRN1* knock-out and knock-down conditions. In the *XRN1* KO cells *L1* reporter mRNA is stabilized, making more of it available for deadenylation and uridylation. Thus *L1* 3′ poly(A) tails are shortened and heavily uridylated. This is accompanied by reduction of the fraction of 5′ methyl-G capped *L1* mRNA and, in result, reduced *L1* translation and RNP formation. Our observations agree with earlier reports on stabilization of uridylated mRNA in *XRN1* knock-down conditions (52), MOV10 RNPase-stimulated uridylation (33), hence uridylation-stimulated decapping by DCP2 (54,55,81), and dependency of L1 translation on 5′ cap (11). Since poly(A) tail on *L1* mRNA is necessary for L1 retrotransposition (32), and uridylation of 3′ end interferes with it (33), we attribute our observations of reduced L1 retrotransposition to the changed dynamics of the *L1* 3′ ends. Specifically, the AU-tail class of 3′ tails encompassing adenine tails appended with one or more uridine residues in the *XRN1*, but also in *DCP2* and the double *DCP2/XRN1* KO cells, becomes distinctively different from the AU-tails in control cells. In comparison to the *XRN1* KO, in *DCP2* KO and the double *DCP2/XRN1* KO cells L1 proteins are at least twice more abundant. L1 retrotransposition is nevertheless reduced in them, though to lesser extend than in the *XRN1* KOs implying that more L1 proteins can rescue some of the defect. We interpret our 3′ RACE-seq data so that nearly half of *L1* reporter mRNA 3′ ends are suboptimal or incompetent for retrotransposition. This fraction seems even higher for endogenous *L1*. While currently speculative it is possible that also long-poly(A)-tailed *L1* mRNA are poor substrates for retrotransposition as they are not yet effectively forming L1 RNPs. Indeed, it was demonstrated that contrary to the initial expectations the effectively translated mRNAs mostly possess relatively short poly(A) tails of 40–50 adenines (82–84). Since endogenous L1 often use downstream polyadenylation signals leading to variability of their 3′ UTRs, it is important to note that *XRN1* knock-down led to mild stabilization of endogenous *L1* mRNAs. This was accompanied by reduction of their poly(A) tails and their increased uridylation.

We observed that formation of L1 bodies is similarly affected in all the KO cells (*XRN1*, *DCP2* and the double KO). Essentially, there are slightly lower numbers of L1 bodies (median of 7) in all of them as compared to the wild-type cells (median of 8), and irrespective of the total amounts of L1 proteins in the cells. Whether and how could the change of *L1* reporter 3′ ends affect L1 body formation? An explanation could be related to kinetic effects of L1-ORF1p production. Indeed, slower kinetics of L1-ORF1p trimerization abolish L1 RNP formation (17). Lower translation kinetics on suboptimal *L1* mRNAs with uridylated oligo(A) tails would preclude effective formation of L1 bodies that we indeed observed in all the KO cells. Although it is currently not clear whether L1 bodies are true L1 retrotransposition intermediates or dead ends for L1 retrotransposition, it has been well established that the potential of different L1-ORF1p mutants to bind RNA and form L1 bodies is correlated with L1 retrotransposition (14,17,85,86).

XRN1 and other post-transcriptional RNA decay factors enriched in P-bodies were reported in the regulation of retrotransposition of the yeast LTR retrotransposons: Ty1 and Ty3 (87–89). Genomic knock-outs of *XRN1* and *DCP2* in yeast resulted respectively in a ∼100–200-fold and ∼10-fold reduction of Ty1 retrotransposition (87,88). This is similar to our observations, although the effects on L1 were smaller. Unlike L1, Ty1 and Ty3 LTRs reverse transcribe within so called virus-like particles (VLPs) that form in the cytoplasm (31,90,91). Ty1 and Ty3 VLPs observed in the *xrn1Δ* yeast cells are bigger in size than in controls (87,92). Furthermore, the steady-state levels of Ty1 RNA in *xrn1Δ* cells were below 50% of the wild-type levels, which matched the reduction in Ty1 Gag protein levels. This is in contrast to the situation observed by us with L1 in the *XRN1* KO cells, where L1 bodies were fewer but similarly sized to those in the wild-type cells. It also seemed that in *xrn1Δ* yeast the processing of p199 protein to integrase and reverse transcriptase was nearly stopped (87,88). Such processing step does not occur with neither L1-ORF1p nor L1-ORF2p. Additionally, Ty1 expresses a *cis-*encoded antisense RNA known to regulate its retrotransposition and accumulating in the *xrn1Δ* yeast. In the case of L1 no such antisense RNA was identified. Interestingly, in human cells XRN1 and DCP1/2 also localize to P-bodies, and MOV10 and TUT4 shown to restrict retrotransposition (33,63), are among the top 3 P-body enriched proteins in 293T cells (93). Moreover, MOV10, TUT4, TUT7, and DIS3L2 involve in nonsense-mediated decay (NMD) (94–96). Since *L1* mRNA is a dicistronic mRNA it is possible that it becomes an NMD substrate. In fact, UPF1 a constitutive NMD-related helicase was found to interact with L1-ORF2p (26). Curiously, in cells depleted of UPF1, despite upregulation of L1 proteins, L1 retrotransposition was significantly lowered (26). In the light of our findings we speculate that in *UPF1* knock-down the *L1* 3′ ends were deadenylated and uridylated, explaining the unexpected downregulation of L1 retrotransposition. Besides parallels between Ty1/3 LTR and L1 retrotransposons’ regulation by XRN1, there are also parallels with the viral pathogens as XRN1 was shown as a virus restriction factor (97). Similarly, uridylation by TUT4/7 was shown to restrict pathogenic viruses (98), and occur prevalently on viral RNA (99).

XRN1 was identified as a possible L1 retrotransposition suppressor in a high-throughput screen aimed at identification of factors involved in L1 retrotransposition (100). This is in contrast to our observations. Since the authors did not follow in details the possible role of XRN1 in the biology of L1 we think that the conditions of the screen (i.e. multiple sequential selection steps with different antibiotics over a prolonged time of 20 days) likely had affected its outcome for some of the targeted genes. The interference would most likely be expected with genes that are generally important for RNA metabolism e.g. *XRN1*, and not *bona fide* L1 regulators, with little or no involvement in other processes.

L1 retrotransposition was shown to preferentially occur during S and G2/M phases of the cell cycle (101–103). We did not observe consistent effects on cell cycle in the different KO cell lines despite consistent changes in L1 retrotransposition. Thus, the observed differences in the rates of L1 retrotransposition in these cells are not related to changes in the cell cycle. XRN1 has also been shown as a negative regulator of autophagy (104), and autophagy was implicated in clearing *L1* RNA and RNPs (105). Thus, *XRN1* KO could potentially lead to a decrease in the amounts of L1 RNPs available for retrotransposition. In contrast to this scenario, our observations clearly support stabilization of *L1* mRNA in the *XRN1* KO cells arguing against the autophagy-driven L1 regulation in this case.

Finally, we observed somewhat elevated levels of apoptosis in the *XRN1* KO cells. Previously, a synthetic lethal interaction between *XRN1* KO and the overexpression of *L1* was reported in p53-deficient cells (106). In our retrotransposition assays there is no p53 depletion. We also observe consistent increases in numbers of L1-retrotransposition-positive *XRN1* KO and wild-type cells in extended retrotransposition assays (11/12 days). We thus think that increased apoptosis in the *XRN1* KO cells is not the determinant of reduced L1 retrotransposition, but we cannot exclude some contribution. *L1* mRNA relies on its poly(A) tail not only in stabilization against nucleases and ensuring efficient translation and RNP formation, but also in the sole TPRT-mediated retrotransposition. Thus the dynamics of *L1* mRNA 3′ ends is most likely generally important for L1. It will certainly be exciting to assess in how far the mechanisms proposed in this paper recapitulate in developmental and health-relevant conditions.

## Supplementary Material

gkad1251_Supplemental_Files

## Data Availability

The high-throughput sequencing data have been deposited on GEO under the accession number GSE248109. The scripts used for data analysis and visualisation are available at https://gitfront.io/r/pbioinf/eUKTgpxukvCY/RACE-Seq/. The high content imaging flow cytometry data and other raw data are available with DOIs: 10.5281/zenodo.7908577, 10.5281/zenodo.10050366, and 10.5281/zenodo.10050244.
