# Supplementary material for: LINE-1 mRNA 3′ end dynamics shape its biology and retrotransposition potential
Source: Nucleic Acids Res. 2024 Jan 9;52(6):3327–45. doi: 10.1093/nar/gkad1251 (PMC11014359; doi:10.1093/nar/gkad1251)
Supplement: gkad1251_Supplemental_Files [file gkad1251_supplemental_files.zip › Supplementary materials_ZW_complete.pdf]

Supplementary Data

LINE-1 mRNA 3' end dynamics shape its biology and retrotransposition potential

AUTHORS

Damian M. Janecki[1,†], Raneet Sen[1,†], Natalia Szóstak[2,†], Arkadiusz Kajdasz[1], Martyna Kordyś[1], Kinga Plawgo[1], Dmytro Pandakov[1],Anna Philips[2], Zbigniew Warkocki[1,*]

[1] Department of RNA Metabolism, Institute of Bioorganic Chemistry, Polish Academy of Sciences, Poznan, 61-704, Poland

[2] Laboratory of Bioinformatics, Institute of Bioorganic Chemistry, Polish Academy of Sciences, Poznan, 61-704, Poland

† Joint Authors

* To whom correspondence should be addressed. Tel: +48618528503 ; Fax: + 48618520532; Email: zwarkoc@gmail.com; zwarkocki@ibch.poznan.pl

Supplementary Methods

3' RACE-seq analysis

From 3'RACE-Seq raw reads, adapters were removed with cutadapt (v4.0) (1) and sequences shorter than 20 nt were discarded. Low-quality nucleotides from 3' end of reads were removed using ConDeTri (v2.3) (2) with the following settings hq=37, ml=1, mh=5 and minlen=40. Specific amplicons were selected based on a sequence of forward primers with bbduk from BBTools (3). For further analyses only reads containing a unique molecular identifier (UMI) in length 15 nt and delimiter (GTCAG) on 5' end of read R2 were kept. UMI and delimiter sequences were removed from read sequences and moved to the read name with the extract mode of UMI-tools (v1.1.2) (4) which would allow deduplicate PCR artifacts after mapping. As references for control transcripts (*GAPDH*, *ACTB*, *PABPC4*) were used gene sequences

provided by the Ensembl database (GRCh38). For *LINE-1* transcripts sequence of plasmid used for *LINE-1* overexpression was used as a reference. Reads were mapped to reference using STAR (v2.7.9a) (5) with soft-clipping allowed (with the following settings outFilterMatchNmin 20, outFilterScoreMinOverLread 0.1 and outFilterMatchNminOverLread 0.1). Mapped reads were deduplicated with UMIcollapse (6).

For pair of reads in which both reads mapped non-templated tails and their nucleotide compositions were retrieved from the sam file from the soft-clipped fragments of read R2 using the previously published script (get_softclipped_reads_from_sam.pl) (7). For pair of reads in which only R1 mapped due to presence of long homopolymeric sequence in the read R2 length and sequence of tails were extracted directly from the read R2. Statistical analyses were performed in *R*. In these analyses were included reads with no tails and those with the following composition: tails containing only A (A tails), only U (U tails), A with U on their 3' (AU tails). In AU tails were allowed tails with U between A but not at the beginning of the tail. All operations on bam and sam files were performed with samtools (v1.13) (8).

Viability assay

For evaluation of cell viability, 20,000 cells were seeded in each well of 96-well plate in three replicates and then substrate and enzyme of the RealTime-Glo™ Cell Viability Assay (Promega) was added, according to the manufacturer's protocol. The luminescent signal, which correlates with the number of viable cells, was measured every 12 h up to 48 h using GloMax® Discover Microplate Reader / Explorer Multimode Microplate Reader (Promega).

Supplementary Note 1

We reproducibly failed in generating *XRN1* KO cells with the human embryonic carcinoma PA-1 cells (9) by using same Cas9 and guide expressing plasmids as with 293T cells. At day 4 post-transfection and following selection with puromycin we observed editing of gDNA within the expected region in the *XRN1* gene in the pool of the cells, but following clonal selection out of 28 clones tested for XRN1 protein by western blot all expressed XRN1 and none was a knock-out clone (not shown). Also PA-1 cells did not survive transfections with siRNA against *XRN1* and L1 retrotransposition reporter plasmid followed by selection with puromycin and TSA as described (L1 retrotransposition assay) (10) unlike the control cells transfected with non-targeting siRNA.

Supplementary Note 2

Reading long homopolymeric tracts like a poly(A) tail by Illumina meets two major obstacles. First, the length of the detected homopolymeric tract is limited to the length of the sequencing read itself. In other words in a 100 plus 100 paired-end sequencing the length of the homopolymeric tract cannot be longer than 100, or shorter if other sequences are included in the read as is the case in this study where 20 nucleotides in the R2 read represent the UMI and the delimiter. Second obstacle is the overestimation of the homopolymeric tracts lengths by the Illumina basecaller as demonstrated in earlier studies (11, 12). However, given that L1 poly(A) tails fall mostly within 76 nucleotide length window as showed in our RNAse H assay (Supplementary Fig. 3K, L, M) and that uridylation of 3’ end is crucial to L1 retrotransposition repression we decided to use Illumina sequencing. By using artificial spike-in sequences we observed that the Illumina basecalling generates a sudden decrease in nucleotide reading quality for nucleotides just after a homopolymeric A tract (T tract in R2), which could be used to refine the protocol for poly(A) reading and length estimations. Thus we think Illumina-based high-throughput sequencing is currently the best to read 3’ terminal non-A nucleotides in high-throughput (13). Unlike ONT Nanopore and Pac-Bio platforms that in the current state-of-the-art protocols use oligo(dT) priming which strongly discriminates against uridylated reads (Z. Warkocki, A. Kajdasz unpublished

data). Furthermore, the sole quality of ONT Nanopore sequencing reads does not allow reliable identification of 3’ non-A nucleotides.

Supplementary Figures

Supplementary Figure 1

A

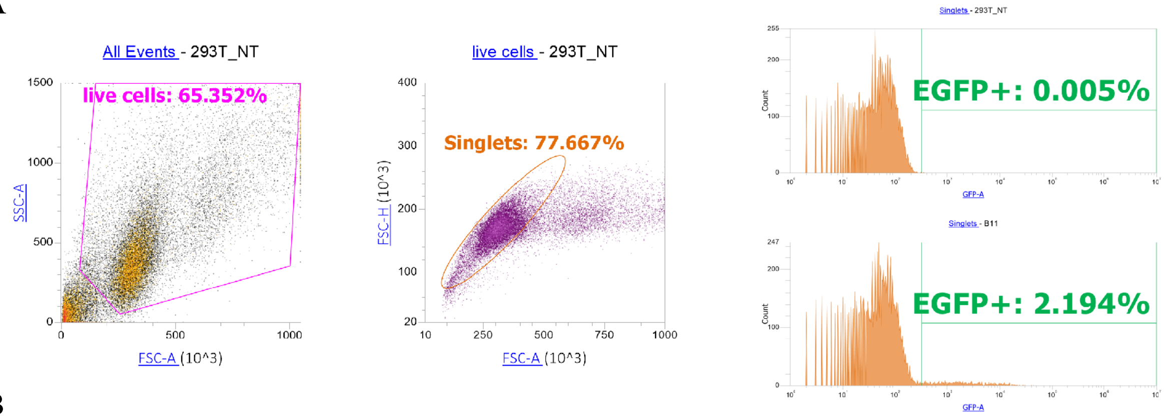

B

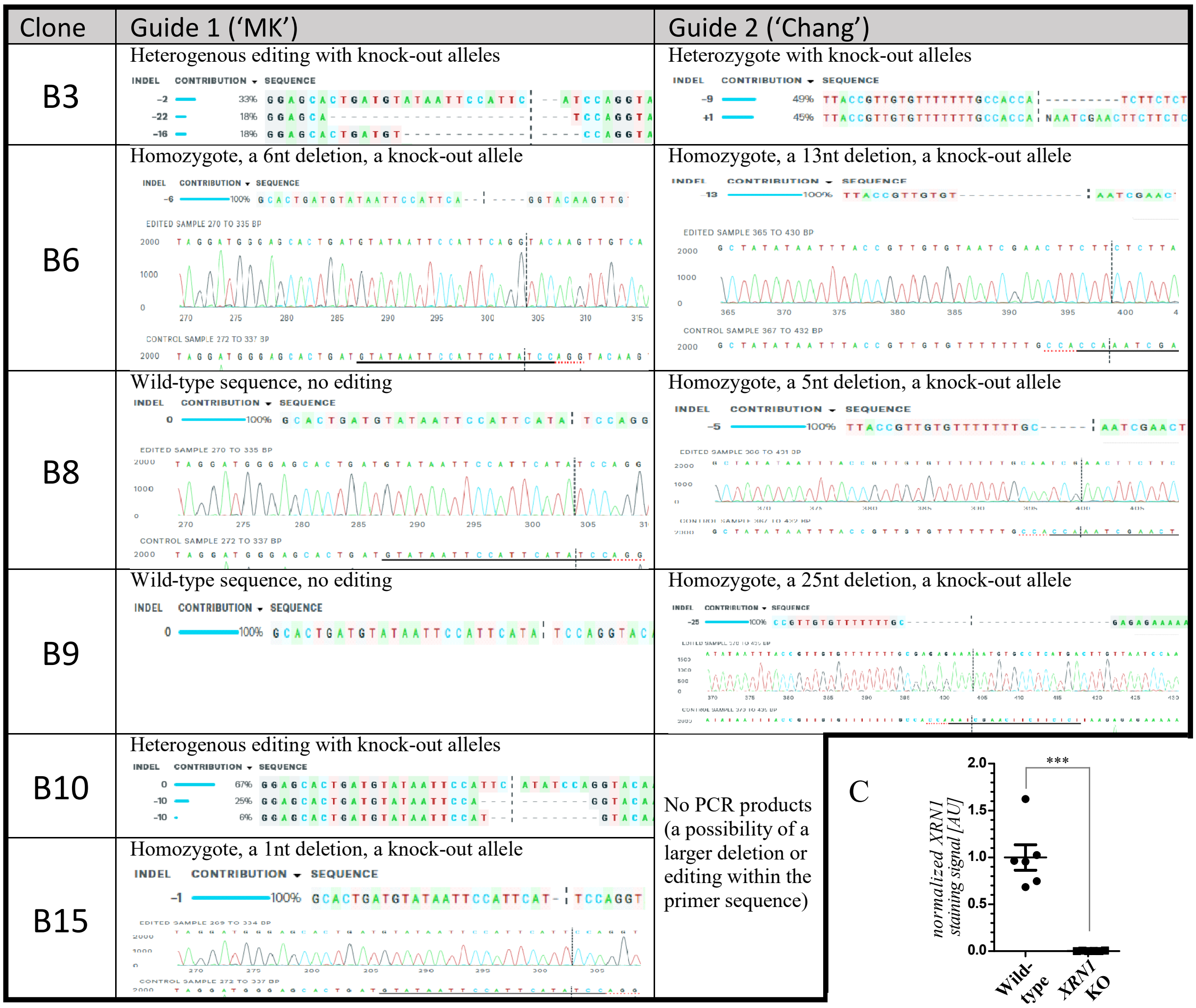

D

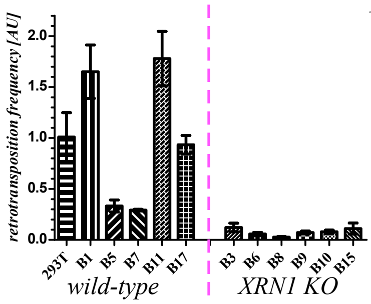

E

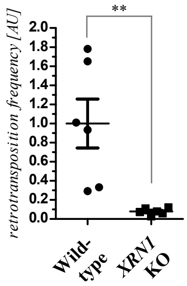

F

	L1 retrotransposition fold change (medians of 4 exps)					
vs KOs	B3	B6	B8	B9	B10	B15
293T	7	15	32	11	10	9
B1	12	24	52	17	17	15
B5	3	6	13	4	4	4
B7	2	5	11	4	3	3
B11	16	32	70	23	22	20
B17	8	16	36	12	11	10
MEDIAN	8	16	34	11	11	10
MEDIAN OF ALL COMBINATIONS =				12		

Supplementary Figure 1. Validation of the 293T XRN1 knock-out clonal cell lines.

(A) An example of the gating strategy used in the analytical flow cytometry in the retrotransposition reporter assays. All cells (events) were first gated for 'live cells' based on FSC-A and SSC-A. This was followed by subgating for 'singlets' using FSC-A and FSC-H parameters. Finally, histogram plots with BL1-A (EGFP; laser λ=488nm, detection filter λ= 530/30nm) were used to set gate for EGFP-positive 'singlets'. (B) Sequencing of the genomic DNA from the respective clonal cell lines (named B3, B6, B8, B9, B10, and B15) in the regions spanning the sequence targeted by the single guide RNA and Cas9 activity. Chromatograms and comparisons to the wild-type sequence were generated using an online tool from Synthego (https://ice.synthego.com/#/). (C) Quantitation of the western blotting results shown in Fig. 1D using Multigauge V3.0 software. Signal intensities were first normalized to GAPDH signals and then to the mean of the wild-type cell lines which was set to 1. Statistical analysis was done by unpaired t-test. (D) Related to Figure 1E. Result of 4 independent L1 retrotransposition assays (including the one presented in Fig. 1E) using the 6 wild-type and 6 *XRN1* KO cell lines. The means of all 6 wild-type conditions were set as 1,00 and used to normalize L1 retrotransposition in the individual wild-type and *XRN1* KO in the individual experiments. Means ±SEM of the normalized data are shown. (E) Another presentation of the data in panel D. Statistical significance was calculated by unpaired t-test. (F) A matrix showing fold change in L1 retrotransposition assay results obtained with the respective wild-type and *XRN1* knock-out clonal cell lines in the 4 independent replications of the experiment. Color-coding generated by the excel software.

Supplementary Figure 2

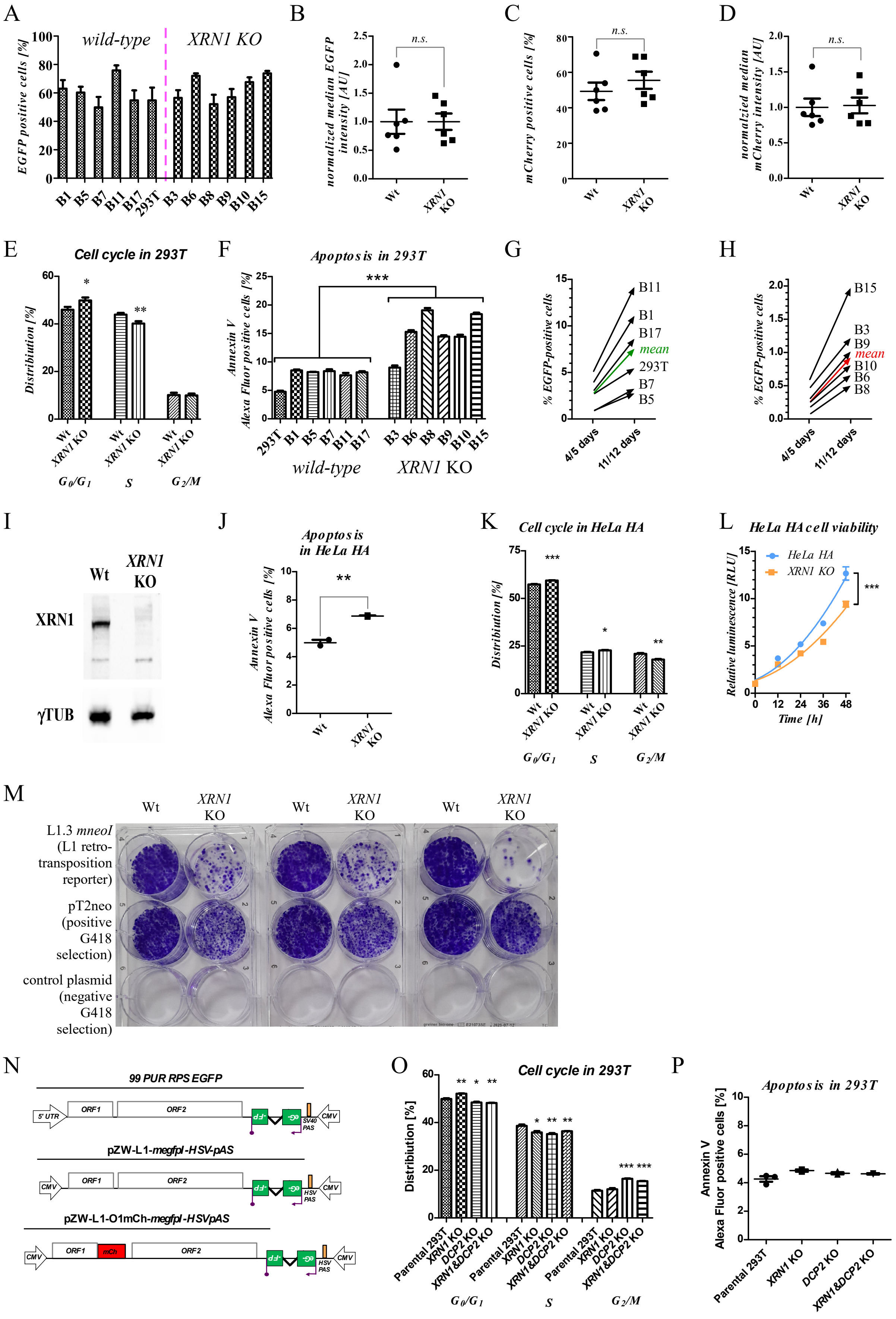

Supplementary Figure 2. Control experiments for the L1 retrotransposition assays in the knock-out 293T cells.

(A) Analytic flow cytometry of the 293T clonal cell lines following transfection with a EGFP expressing plasmid to assess the % of EGFP expressing cells after 48h post-transfection. (B) normalized (mean of wild-type = 1,0) median intensity of EGFP in EGFP-expressing cells from panel A. Lack of statistical significance in this panel and the other panels was calculated by unpaired t-test in reference to the control(s). (C) As in panel A but following transfection with a mCherry expressing plasmid. (D) As in panel B. Normalized median mCherry intensity in the mCherry expressing cells. (E) Distribution of cells at defined cell cycle stages as assessed by propidium iodide staining, flow cytometry and analysis using ModFit LT™ 6.0 software (Verity Software House). (F) Levels of apoptosis were assessed by Annexin V staining using the Annexin V, Alexa Fluor 568 conjugate (Thermo Fisher Scientific) and flow cytometry. (G) Percentages (means of 3 independent experiments) of EGFP-positive wild-type cells (cells which underwent retrotransposition of the L1 reporter) at day 4/5 and day 11/12 post-transfection with the reporter. Pools of cells from the same wells were analysed at both days. Green line indicates a mean of all. (H) Same as in panel G but for the *XRN1* KO cell lines. Red line indicates a mean of all. (I) Western blot validation of the HeLa HA *XRN1* knock-out clonal cell line. Probing for XRN1 and γTubulin were performed on pieces of the same blot. (J) The percentages of apoptotic cells in the wild-type and in the *XRN1* KO HeLa HA cells were estimated as in panel F. (K) Cell cycle in the wild-type and *XRN1* KO HeLa HA cells were estimated as in panel E. (L) Viability of the HeLa HA wild-type and *XRN1* KO cells was assessed over 48hrs using RealTime-Glo™ Cell Viability Assay (Promega). Statistical significance was calculated by F-test. (M) Results of an alternative L1 retrotransposition assay in HeLa HA cells. The wild-type and *XRN1* KO HeLa HA cells were transfected with either of 3 plasmids: L1.3 mneoI (encoding L1 reporter conferring resistance to G418), pT2neo (a

positive control plasmid encoding a G418-resistance gene), pKK-TEV-EGFP (14) (a pcDNA5 FRTTO-based plasmid encoding EGFP, control for transfection and a negative control for G418 selection). After 4 days post-transfection selection with 450µg/ml G418 was started and continued with media change every second day for the next 2 weeks. G418-resistant colonies were stained with crystal violet and photographed. Three independent experiments are shown. (N) A cartoon representation of the constructs used in Figure 1G and J (O1mCh). Only parts of the reporter plasmids encoding L1 and the reporter cassettes are shown. (O) Cell cycle in the 293T knock-out cells analysed as in panel E. (P) Apoptosis in the 293T knock-out cells analysed as in panel F.

Supplementary Figure 3

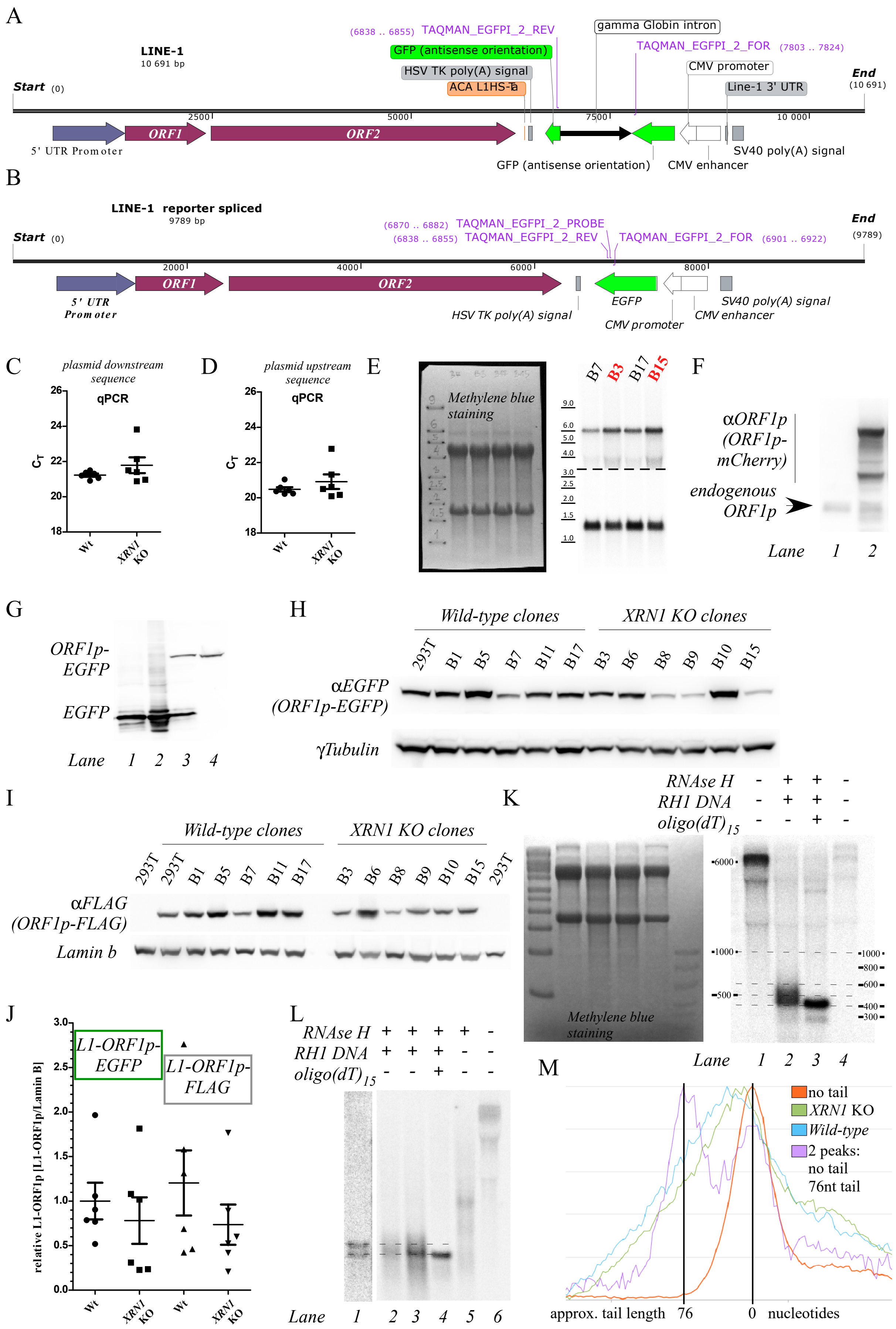

Supplementary Figure 3. Effect of XRN1 KO on L1 reporter RNA and proteins levels.

(A) Graphical representation of the part of the L1 *egfpI* reporter plasmid comprising the full-length RC L1 and the reporter cassette. (B) The part of the L1 *egfpI* reporter after splicing. Note that the numerical coordinates were kept to match the plasmid DNA sequence, parts of which do not exist in the spliced L1 reporter mRNA. In (A) and (B) locations of elements of L1 and the reporter cassette and the binding sites of the Taqman probe and the primers used in the RT-qPCR are indicated. The drawings were generated by SnapGene Viewer and refined using Canvas software. (C and D) Quantitative PCR on DNA isolated from the cells used for generating Fig. 3A. Primers binding to the plasmid sequence upstream or downstream of the RC L1 and the reporter cassette were used. Ct values of qPCR are shown. (E) Methylene blue staining of the nylon membrane (left) and the respective radioactive signals (right) after probing for L1 (top) or GAPDH (bottom). RNA size ladder is indicated on the stained membrane and translated onto the blots. Dashed line (right) indicates the cut of the blot for hybridization with L1 (top) and GAPDH (bottom) probes. Same blots are shown in Fig. 3B (left part). The reason of presenting them again in the Supplementary Figure is to show the bands in the context of the methylene blue stained membrane and of the RNA length ladders. (F) Western blot demonstrating a change in migration of the ORF1p-mCherry as compared to wild-type non-tagged ORF1p. Probing was done using αORF1p antibody. Lane 1 – non-transfected 293T cells, lane 2 – 293T cells transfected with the pZW-O1mCh reporter. Proteins were separated on a 10% SDS-PAGE. (G) Western blot showing a change in migration of ORF1p-EGFP as compared to EGFP. Probing was done with a monoclonal antibody against EGFP. Lanes 1, 2 – 293T cells transfected with a plasmid encoding EGFP, lanes 3, 4 – 293T cells transfected with the pVAN583 plasmid (ORF1p-EGFP). There is a leak from lane 3 to 4. (H) Western blot showing expression of full-length ORF1p-EGFP fusion protein in the analysed cell lines. γTubulin is a loading control on part of the same blot.

(I) Western blot of endogenous ORF1p in the analysed cell lines as indicated. Lamin B was used as a loading control. Shown are parts of the same blot. Proteins were separated on a gradient 4-20% Tris-glycins SDS PAGE. Note the flanking lanes: without transfection with any L1 reporter plasmid (left), and with transfection with the L1.3 nomarker L1 reporter plasmid (right, no FLAG tag in either). (J) Quantitations of the blots presented in panels H and I done by MultiGauge 3.0 software. Normalization were done to γtubulin or lamin B respectively. (K) RNase H experiment to show the lengths of non-templated 3' ends on L1 reporter (L1.3 nomarker) mRNAs. RH1 DNA oligo binds within the 3' part of the *L1 ORF2* and the oligo(dT)$_{15}$ binds to poly(A) tails. In the presence of both oligos RNase H generates a ~440 nucleotide band (no tail), omitting oligo(dT)$_{15}$ preserves the non-templated 3' tails which length can be approximately estimated by comparing to the migration markers. Lane 1 – no RNase H, no DNA oligonucleotides, full length L1, lane 2 – following addition of RNase H and RH1 oligonucleotide to show non-templated tails, lane 3 – as in lane 2 plus oligo(dT)$_{15}$ to set the "no tail" reference, lane 4 – no transfection, endogenous *L1*. Migration RNA ladders' lengths are indicated and extrapolated by dashed lines. The "no tail" band is 440 nucleotides on average (RNase H might cut in multiple places within the RNA hybridized to DNA oligonucleotides) thus "500" marks tails of ~60 nucleotides and "600" marks tails of ~160 nucleotides (hardly detectable, likely effect of smearing). Bands were visualized by Northern blot. (L) RNase H assay as in panel K but for samples from the wild-type and the *XRN1* KO cells. Migration ladders define "no tail" and a 76-nucleotide tail (lane 1). Lane 2 – wild-type, lane 3 – *XRN1* KO, lane 4 – "no tail", lane 5 –incubated with RNase H but without addition of any DNA oligonucleotide, lane 6 – no treatment. (M) Analysis of the blot in panel K following normalization of signals to a common maximum. Respective conditions are color-coded as indicated. Lines indicate "no tail" and 76-nucleotide tail. Note significant smearing of the signal that affects resolution.

Supplementary Figure 4

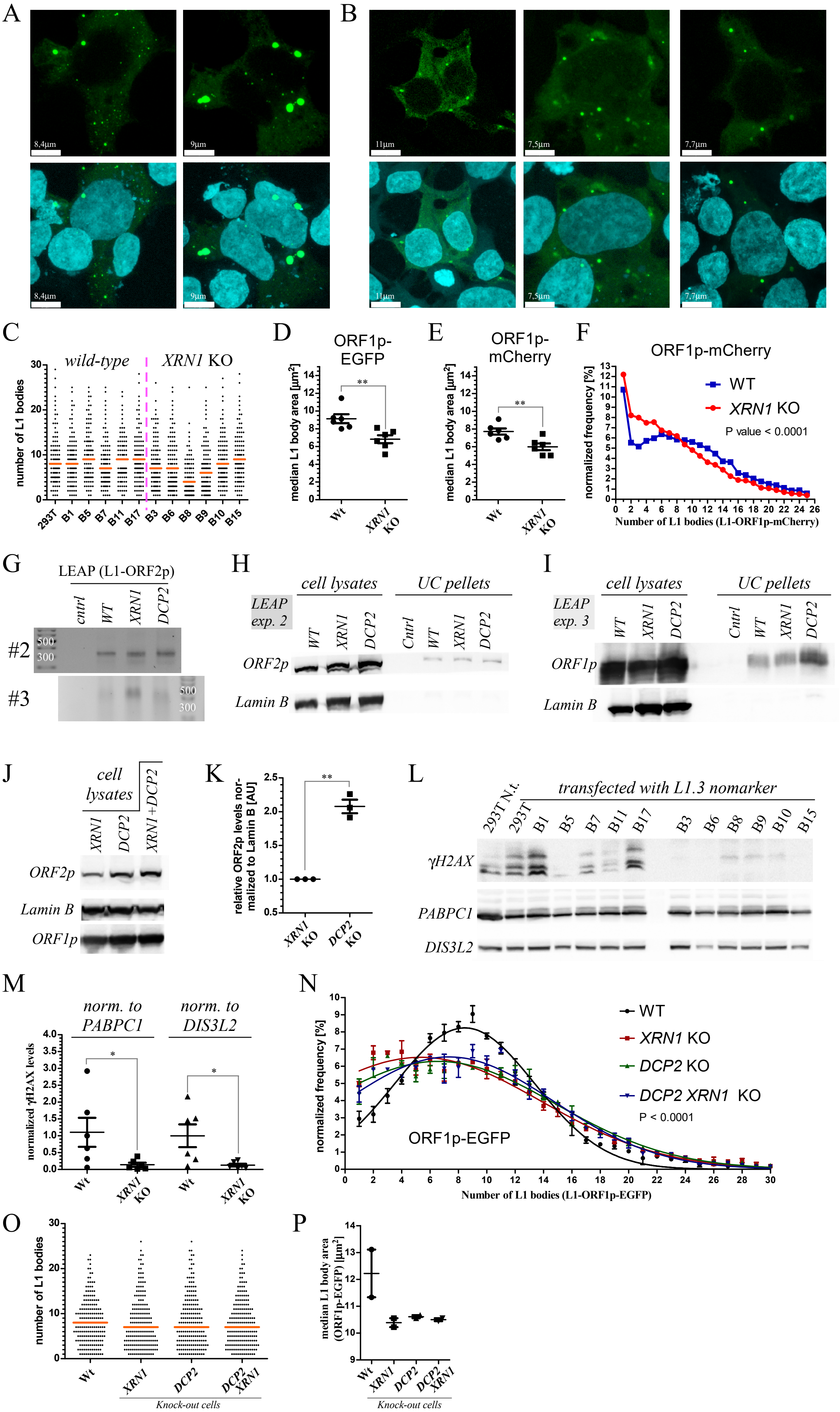

Supplementary Figure 4. L1 bodies and ORF2p in the wild-type and XRN1 KO cells.

(A) Example of confocal microscopy z-stacks of wild-type 293T cells transfected with a ORF1p-EGFP expressing plasmid and formaldehyde-fixed before microscopic analysis. L1-ORF1p-EGFP is in green. L1 bodies are seen as intense green spots in the cytoplasm of the cells. Nuclei were stained with Hoechst and are visualized in cyan in the merged pictures. White bar is shown to scale (8,4 or 9μm). (B) Same as in panel A but *XRN1* KO cells were visualized and the scales are 11, 7,5, and 7,7μm. (C) Results of the high-throughput imaging flow cytometry. Dots (scaled to a total ~100 for each data set) represent cells with the indicated number of L1 bodies. Orange lines indicate medians of L1 bodies in the respective cell lines. (D) Quantitation of the L1-ORF1p-EGFP containing L1 bodies' sizes (in μm^2) in the 6 wild-type and 6 *XRN1* KO cell lines performed by high-content imaging flow cytometry for thousands of cells following nuclei staining with Hoechst. Each point represents a median area for a cell line. Statistical significance was calculated by unpaired t-test. (E) Same as in panel D but for cells transfected with L1-ORF1p-mCherry expressing plasmid. (F) Number of L1 bodies in the wild-type and *XRN1* KO cells after transfection with the L1-ORF1p-mCherry expressing plasmid. Related to data shown in panel E. Statistically significant difference between the fits was calculated using F-test. (G) Two independent replicates of the LEAP assay in the wild-type, *XRN1*, and *DCP2* KO cells. White numbers over ladder bands indicate the length of the DNA band. (H)(I) Western blots on cell lysates and ultracentrifuged materials (UC pellets) from the 2nd and 3rd repetition of the LEAP assay. Shown are probing for L1-ORF2p, L1-ORF1p, and Lamin B(loading control and control of purity of the UC pellets). (J) Western blot on cell lysates from *XRN1*, *DCP2*, and the double *DCP2* and *XRN1* KO cells to show L1-ORF2p, L1-ORF1p and Lamin B (loading control) following transfection with the JM101/L1.3 nomarker plasmid. (K) Quantitation of L1-ORF2p levels in the *XRN1* and *DCP2* KO cells following transfection with JM101/L1.3 nomarker plasmid. Data points

represent three independent biological replicates. Values in the *XRN1* KO cells were set to 1,00, and the corresponding values in the *DCP2* KO were calculated in regards to their matching *XRN1* KO signals. Statistical significance was calculated by paired t-test. (L) Western blot on cell lysates from the 6 wild-type and 6 *XRN1* KO cell lines. Probing was done for γH2AX, a phosphorylated H2AX form indicative of dsDNA breaks, PABPC1 and DIS3L2 (loading controls). (M) Quantitation of signals of γH2AX in panel L, normalized to either PABPC1 or DIS3L2 signal and with mean values of the wild-type conditions set to 1,00. Statistical significance was calculated by unpaired t-test. (N) Results of high content imaging flow cytometry on live wild-type, *XRN1*, *DCP2*, or the double *DCP2* and *XRN1* KO cells transfected with L1-ORF1p-EGFP expressing plasmid and nuclei stained with Hoechst. Two independent transfections and analyses were performed for each cell line and mean values are shown. Statistically significant difference between each pair of the fits was calculated by F-test. (O) Results from panel N. Dots (scaled to ~100 for each data set) represent cells with the indicated number of L1 bodies. Orange lines indicate median number of L1 bodies in the respective cell lines. (P) Median L1 body areas (in μm^2) as assessed in the indicated cell lines.

Supplementary Figure 5

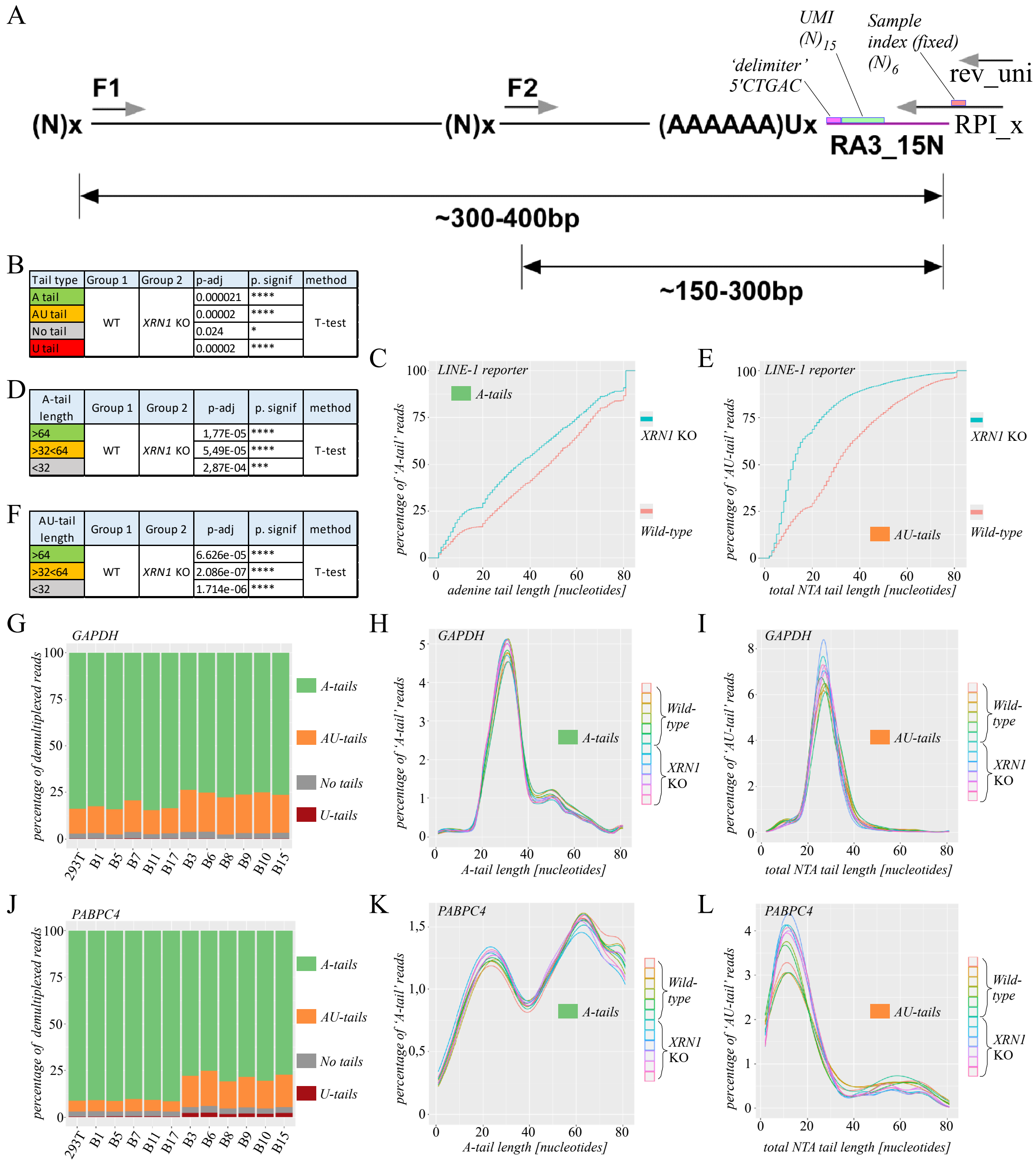

B

Tail type	Group 1	Group 2	p-adj	p. signif	method
A tail	WT	*XRN1* KO	0.000021	****	T-test
AU tail			0.00002	****	
No tail			0.024	*	
U tail			0.00002	****	

D

A-tail length	Group 1	Group 2	p-adj	p. signif	method
>64	WT	*XRN1* KO	1,77E-05	****	T-test
>32<64			5,49E-05	****	
<32			2,87E-04	***	

F

AU-tail length	Group 1	Group 2	p-adj	p. signif	method
>64	WT	*XRN1* KO	6.626e-05	****	T-test
>32<64			2.086e-07	****	
<32			1.714e-06	****	

Supplementary Figure 5. High-throughput 3' RACE-seq of L1 and control mRNAs.

(A) Graphical representation of the 3' RACE-seq library preparation procedure. A preadenylated 3' adapter comprises unique molecular identifier $(N)_{15}$ for demuliplexing. Following ligation and reverse transcription with a RPI_x primer comprising a 6-nucletide index sequence (for each biological/technical replicate) semi-nested PCR is carried out with the indicated forward primers (F1, F2) and a reverse primer (rev_uni) that is universal to all libraries. (B) A report on statistical analysis to compare the wild-type and *XRN1* KO cells. The differences between the contributions of each of the 4 tail classes to the total non-templated tails on L1 were analysed. (C) A cumulative plot showing lengths of A-tails on L1 reporter mRNA in the wild-type and *XRN1* KO cells as indicated. (D) A report on statistical analysis on the A-tails lengths on L1 reporter mRNA in the wild-type and *XRN1* KO cells related to Fig. 5C. (E) A cumulative plot showing total lengths of AU-tails (A plus U) on L1 reporter mRNA in the wild-type and *XRN1* KO cells as indicated. (F) A report on statistical analysis on the AU-tails lengths on L1 reporter mRNA in the wild-type and *XRN1* KO cells related to Fig. 5E. (G) Distribution of A-tails, AU-tails, no tails, and U-tails in the 3' RACE-seq data on endogenous *GAPDH* transcripts in the different clonal cell lines. (H) A graph showing length distribution of the A-tails on *GAPDH* mRNA in the range from 1 to ≥81 A in all analysed cell lines as indicated. (I) A graph showing length distribution of the AU-tails on *GAPDH* mRNA in the range from 1 to ≥81 nucleotides. (J) Distribution of A-tails, AU-tails, no tails, and U-tails in the 3' RACE-seq data on endogenous *PABPC4* transcripts in the different clonal cell lines. (K) A graph showing length distribution of the A-tails on *PABPC4* mRNA in the range from 1 to ≥81 A. (L) A graph showing length distribution of the AU-tails on GAPDH mRNA in the range from 1 to ≥81 nucleotides.

Supplementary Figure 6

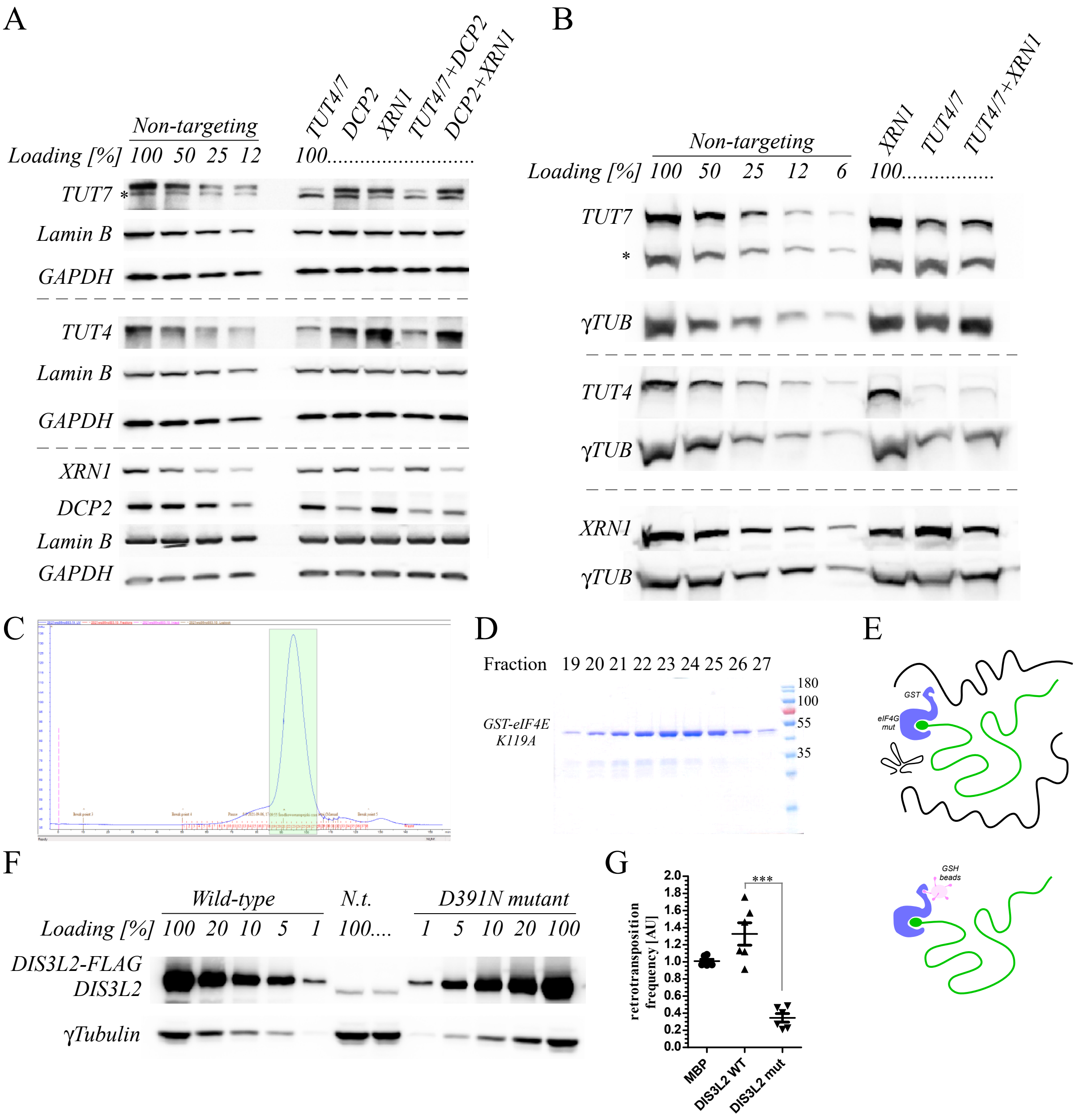

Supplementary Figure 6. Control and preparatory experiments related to Fig. 6.

(A)(B) Western blot analyses of the effectiveness of knock-down of the indicated proteins by RNAi. Samples were collected at 48h post-transfection with siRNA and the L1 reporter plasmid. Probed proteins are indicated. Dashed lines divide individual blots. Lamin B and GAPDH were used as loading controls. Panel A – samples were run on a gradient pre-cast Novex 4-20% Tris-glycine SDS PAA gel (ThermoFisher Scientific). Panel B – samples were run on a 8% hand-cast home-made SDS-PAGE. Asterisk indicates an unspecific band routinely seen in TUT7 blots. The difference in migration is a result of the two different gel types used. (C) A detail of the size-exclusion chromatography of GST-eIF4E preparation. Green shading over selected fractions. (D) SDS-PAGE analysis and Coomasie staining of the GST-eIF4E mutant protein in the respective fractions (green in panel C). (E) A graphical demonstration of the capped RNA selection protocol with the GST-eIF4E mutant protein. (F) Western blot showing overexpression of wild-type or D391N mutated DIS3L2 protein and a titration to compare to the wild-type endogenous DIS3L2 levels. Note difference in migration is the effect of the 6-His linker and Flag tags on the ectopically expressed proteins. γTubulin is a loading control on part of the same blot. (G) L1 retrotransposition assay following overexpression of either wild-type or D391N mutated DIS3L2, or maltose binding protein (control). Statistically significant difference between the conditions was calculated by ANOVA followed by Tukey's test and is shown for the two DIS3L2 conditions.

Supplementary Figure 7

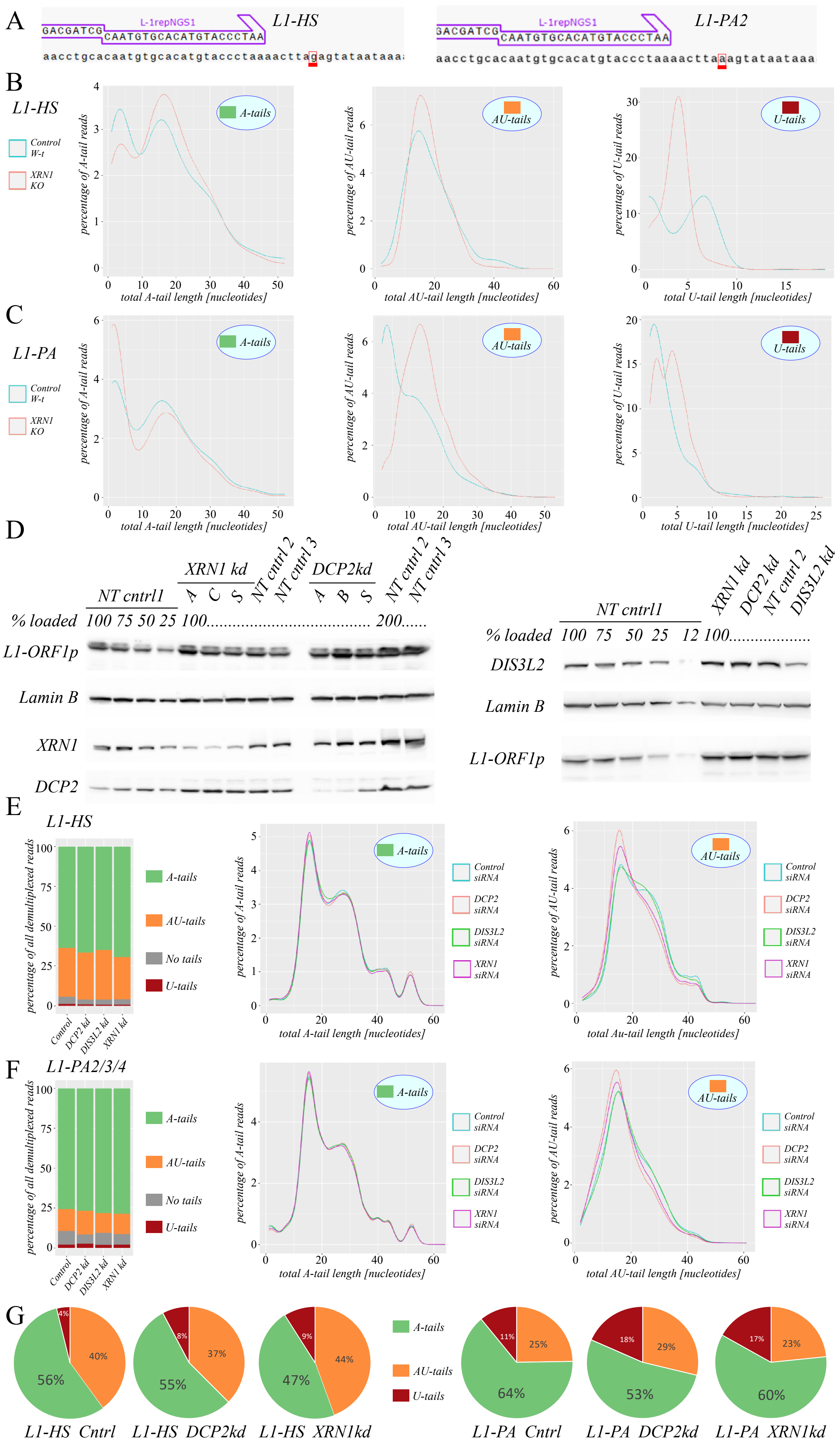

Supplementary Figure 7. The effects of XRN1 and DCP2 knock-downs by RNAi on endogenous L1 in 293T and PA-1 cells. Related to Figure 7.

(A) Graphical presentation of the binding sites of primer used in 3' RACE-seq library preparation (L-1repNGS1) to endogenous *L1* sequences. Red frame shows a nucleotide polymorphism used to differentiate *L1*HS from *L1* –PA2 (and older *L1*-PA3/4). Sequences after https://www.girinst.org/repbase (B) 3' RACE-seq results for endogenously expressed *L1*-HS in the 293T wild-type and *XRN1* KO cells as indicated. Both the control and *XRN1* KO sum up results for 6 individual clonal cell lines. The lengths and percentages of total demultiplexed reads are shown for the A-tails, AU-tails, and U-tails as indicated. (C) As in panel B but results for endogenously expressed *L1*-PA2/3/4. (D) Western blot analyses of the effectiveness of knock-down of the indicated proteins in PA-1 by RNAi. Cells were collected at 48h post-transfection with siRNA, lysed, and lysates loaded onto SDS-PAGE as indicated. '% loaded' means how much sample was loaded in regards to an adjusted '100%'. The titration is set to help direct visual comparison of the effectiveness of the knock-down and changes in L1-ORF1p expression. Probed proteins are indicated. Lamin B was used as the loading control. (E) A composition of panels summarizing 3' RACE-seq of endogenous evolutionarily young *L1-HS* from PA-1 cells following temporal (48h) knock-down of *XRN1*, *DCP2*, and *DIS3L2* by RNAi and the resulting depletion of the proteins. The left panel shows the percentages of *L1*-HS 3' ends falling into one of the 4 categories. The middle panel shows the A-tails lengths distributions, and the right panel shows the AU-tails length distributions. The conditions are color-coded as indicated. (F) A set of panels as in E but for the evolutionarily older L1 classes (*L1*-PA2/3/4). (G) 3' RACE-seq results on relative presence of tailed *L1*-HS and *L1*-PA2/3/4 in PA-1 cells following long (126h, two consecutive transfections with siRNA at time-points 0h and 72h) RNAi-mediated depletion of *XRN1*, *DCP2*, or control conditions.

Supplementary Tables

Generation of *XRN1* knock-out cells	
XRN1MK_top	[Phos]CACCgGTATAATTCCATTCATATCC
XRN1MK_bot	[Phos]AAACGGATATGAATGGAATTATAC
XRN1 Chang_top	[Phos]CACCgAGAGAAGAAGTTCGATTTGG
XRN1 Chang_bot	[Phos]AAACCCAAATCGAACTTCTTCTCT
hU6_F	GAGGGCCTATTTCCCATGATTCC
Validation of editing of the *XRN1* gene and validation of the knock-out cells by PCR	
XRN1_MK-F1	TGAGATATTTCCATTTCTGGGGGA
XRN1_MK-R1	AGTTTTTGTATAATAGAATACACAAATCC
XRN1_Chang-F1	GTGGAAGGATTCTCAGAATTAAGAA
XRN1_Chang-R1	GTTTCTTATTCTTCCTTGTATAGAATTG
Cloning *XRN1* gene and its mutant variants	
XRN1_for	TCCGAAAACCTGTACTTCCAAGGAACCGGTATGGGAGTCCCCAAGTTTTAC
XRN1_rev	ATCACCCTGAAAATACAAATTCTCGCTAGCTTACTCAGAAGGTTTAGAAACA C
X1_R100A_R101A_for	GCAGCGTGGGGCGGGTTTTAGGTCAGCAAAGGAGGC
X1_R100A_R101A_re v	CTAAAACCCGCCCCACGCTGCTGGT
X1_D206N_D208N_for	TCTTTATGGTTTAAATGCTAACTTGATTATGCTTGGATTAACAAGTCATGAGG
X1_D206N_D208N_rev	ATAATCAAGTTAGCATTTAAACCATAAAGACAGTGTCTGGTGTTTGG
qPCR to assess *L1* reporter cDNA quantities	
TAQMAN_EGFPI_2_FOR	GGTGAACTTCAAGATCCGCCAC
TAQMAN_EGFPI_2_REV	GTCGCCGATGGGGGTGTT
TAQMAN_EGFPI_2_PROBE	CGGCCAGCTGCAC
RNase H assay	
ORF2_RH1	TTTACTGAGAATGATGGTTTCCAAT
3UTR_RH1	CCACAGTCCCCAGAGTGTGATATTCC
LEAP assay	
LEAP_12T	GCGAGCACAGAATTAATACGACTCACTATAGGTTTTTTTTTTTTVN
LEAP_rev	GCGAGCACAGAATTAATACGACT
3UNP1f2	AGATATACCTAATGCTAGATGAC

Supplementary Table 1. DNA oligonucleotides used to generate cell lines, perform cloning, RT-qPCR, RNase H, and LEAP assays.

Names of the procedures are followed by name of the DNA oligonucleotide and its sequence. Note [Phos] indicates a 5′ phosphate, incorporated into DNA to facilitate cloning.

Adapter ligated to RNA 3′ ends (with the unique molecular identifier sequence)	
RA3_15N	/5rApp/CTGACNNNNNNNNNNNNNNNTGGAATTCTCGGGTGCCAAGG/3ddC/
Primers used for reverse transcription (barcoded)	
RPI_10	CAAGCAGAAGACGGCATACGAGATAAGCTAGTGACTGGAGTTCCTTGGCACCCGAGAATTCCA
RPI_12	CAAGCAGAAGACGGCATACGAGATTACAAGGTGACTGGAGTTCCTTGGCACCCGAGAATTCCA
RPI_13	CAAGCAGAAGACGGCATACGAGATTTGACTGTGACTGGAGTTCCTTGGCACCCGAGAATTCCA
RPI_14	CAAGCAGAAGACGGCATACGAGATGGAACTGTGACTGGAGTTCCTTGGCACCCGAGAATTCCA
RPI_16	CAAGCAGAAGACGGCATACGAGATGGACGGGTGACTGGAGTTCCTTGGCACCCGAGAATTCCA
RPI_17	CAAGCAGAAGACGGCATACGAGATCTCTACGTGACTGGAGTTCCTTGGCACCCGAGAATTCCA
RPI_18	CAAGCAGAAGACGGCATACGAGATGCGGACGTGACTGGAGTTCCTTGGCACCCGAGAATTCCA
RPI_19	CAAGCAGAAGACGGCATACGAGATTTTCACGTGACTGGAGTTCCTTGGCACCCGAGAATTCCA
RPI_20	CAAGCAGAAGACGGCATACGAGATGGCCACGTGACTGGAGTTCCTTGGCACCCGAGAATTCCA
RPI_21	CAAGCAGAAGACGGCATACGAGATCGAAACGTGACTGGAGTTCCTTGGCACCCGAGAATTCCA
RPI_22	CAAGCAGAAGACGGCATACGAGATCGTACGGTGACTGGAGTTCCTTGGCACCCGAGAATTCCA
RPI_23	CAAGCAGAAGACGGCATACGAGATCCACTCGTGACTGGAGTTCCTTGGCACCCGAGAATTCCA
RPI_24	CAAGCAGAAGACGGCATACGAGATGCTACCGTGACTGGAGTTCCTTGGCACCCGAGAATTCCA
RPI_25	CAAGCAGAAGACGGCATACGAGATATCAGTGTGACTGGAGTTCCTTGGCACCCGAGAATTCCA
RPI_26	CAAGCAGAAGACGGCATACGAGATGCTCATGTGACTGGAGTTCCTTGGCACCCGAGAATTCCA
RPI_27	CAAGCAGAAGACGGCATACGAGATAGGAATGTGACTGGAGTTCCTTGGCACCCGAGAATTCCA
RPI_28	CAAGCAGAAGACGGCATACGAGATCTTTTGGTGACTGGAGTTCCTTGGCACCCGAGAATTCCA
RPI_29	CAAGCAGAAGACGGCATACGAGATTAGTTGGTGACTGGAGTTCCTTGGCACCCGAGAATTCCA
RPI_30	CAAGCAGAAGACGGCATACGAGATCCGGTGGTGACTGGAGTTCCTTGGCACCCGAGAATTCCA
Primers for PCR#1, complementary to cDNA, and the universal 3′ primer	
3UNP1f2	AGATATACCTAATGCTAGATGAC
GAPDH_3R0	CTGAGCACCAGGTGGTCT
037_3R1	GGTTCTGCTGTGGCCCAGAC
Primers for PCR#2, including 5' part that is essential for sequencing on a Illumina platform and 3' ends complementary to the cDNA-specific sequence	
L1_NGS4	AATGATACGGCGACCACCGAGATCTACACGTTCAGAGTTCTACAGTCCGACG ATCTTTGTGAAATTTGTGATGCTATTG
GAPDH_NGS	AATGATACGGCGACCACCGAGATCTACACGTTCAGAGTTCTACAGTCCGACG ATCCTAGGGAGCCGCACCTTGT
PABPC4_NGS	AATGATACGGCGACCACCGAGATCTACACGTTCAGAGTTCTACAGTCCGACG ATCCCCAGAAATTGGTTTTATTT
L-1rep NGS1	AATGATACGGCGACCACCGAGATCTACACGTTCAGAGTTCTACAGTCCGACGATC GCAATGTGCACATGTACCCTAA

Supplementary Table 2. DNA oligonucleotides used to generate RACE-seq libraries.

Names of the procedures are followed by name of the DNA oligonucleotide and its sequence. Note /5rApp/ and /3ddC/ indicate preadenylated 5′ and dideoxyC at the 3′ respectively. N is any nucleotide.

Supplementary References

1. Martin,M. (2011) Cutadapt removes adapter sequences from high-throughput sequencing reads. *EMBnet.journal*, **17**, 10–12.
2. Smeds,L. and Künstner,A. (2011) ConDeTri--a content dependent read trimmer for Illumina data. *PLoS One*, **6**.
3. Bushnell B (2016) BBMap download | SourceForge.net. *BBMap short read aligner*.
4. Smith,T., Heger,A. and Sudbery,I. (2017) UMI-tools: Modeling sequencing errors in Unique Molecular Identifiers to improve quantification accuracy. *Genome Res.*, **27**, 491–499.
5. Dobin,A., Davis,C.A., Schlesinger,F., Drenkow,J., Zaleski,C., Jha,S., Batut,P., Chaisson,M. and Gingeras,T.R. (2013) STAR: ultrafast universal RNA-seq aligner. *Bioinformatics*, **29**, 15–21.
6. Liu,D. (2019) Algorithms for efficiently collapsing reads with Unique Molecular Identifiers. *PeerJ*, **7**, e8275.
7. Warkocki,Z., Krawczyk,P.S., Adamska,D., Bijata,K., Garcia-Perez,J.L. and Dziembowski,A. (2018) Uridylation by TUT4/7 Restricts Retrotransposition of Human LINE-1s. *Cell*, **174**, 1537-1548.e29.
8. Danecek,P., Bonfield,J.K., Liddle,J., Marshall,J., Ohan,V., Pollard,M.O., Whitwham,A., Keane,T., McCarthy,S.A. and Davies,R.M. (2021) Twelve years of SAMtools and BCFtools. *Gigascience*, **10**.
9. Thomson,J.A., Itskovitz-Eldor,J., Shapiro,S.S., Waknitz,M.A., Swiergiel,J.J., Marshall,V.S. and Jones,J.M. (1998) Embryonic stem cell lines derived from human blastocysts. *Science*, **282**, 1145–1147.
10. Garcia-Perez,J.L., Morell,M., Scheys,J.O., Kulpa,D.A., Morell,S., Carter,C.C., Hammer,G.D., Collins,K.L., O'Shea,K.S., Menendez,P., *et al.* (2010) Epigenetic silencing of engineered L1 retrotransposition events in human embryonic carcinoma cells. *Nature*, **466**, 769–73.
11. Chang,H., Lim,J., Ha,M. and Kim,V.N. (2014) TAIL-seq: genome-wide determination of poly(A) tail length and 3′ end modifications. *Mol. Cell*, **53**, 1044–52.
12. Yu,F., Zhang,Y., Cheng,C., Wang,W., Zhou,Z., Rang,W., Yu,H., Wei,Y., Wu,Q. and Zhang,Y. (2020) Poly(A)-seq: A method for direct sequencing and analysis of the transcriptomic poly(A)-tails. *PLoS One*, **15**, e0234696.
13. Brouze,A., Krawczyk,P.S., Dziembowski,A. and Mroczek,S. (2023) Measuring the tail: Methods for poly(A) tail profiling. *Wiley Interdiscip. Rev. RNA*, **14**.
14. Szczesny,R.J., Kowalska,K., Klosowska-Kosicka,K., Chlebowski,A., Owczarek,E.P., Warkocki,Z., Kulinski,T.M., Adamska,D., Affek,K., Jedroszkowiak,A., *et al.* (2018) Versatile approach for functional analysis of human proteins and efficient stable cell line generation using flp-mediated recombination system. *PLoS One*, **13**, e0194887.
15. Koblitz,J., Steenpass,L., Dirks,W.G., Eberth,S., Nagel,S. and Pommerenke,C. (2022) DSMZCellDive: Diving into high-throughput cell line data. *F1000Research*, **11**.

The original uncropped blots

Figure 1B

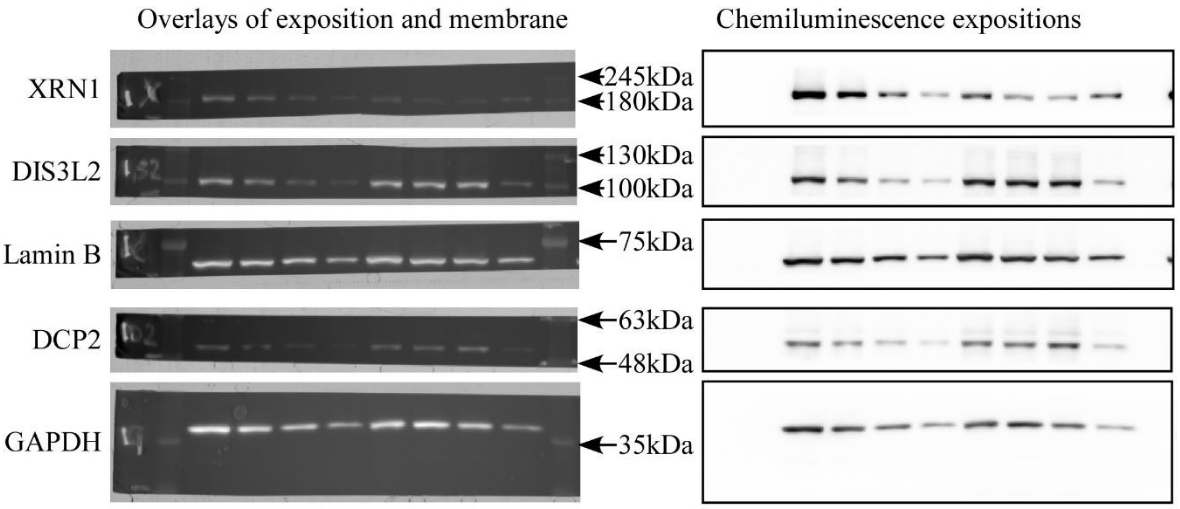

nitrocellulose membrane after transfer and ponceau S staining
before and after marking and cutting into individual pieces

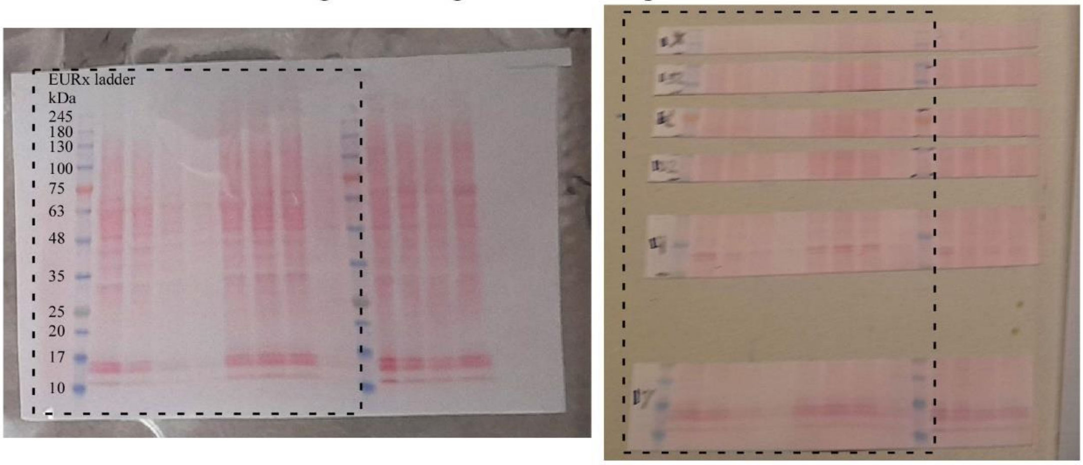

Figure 1D

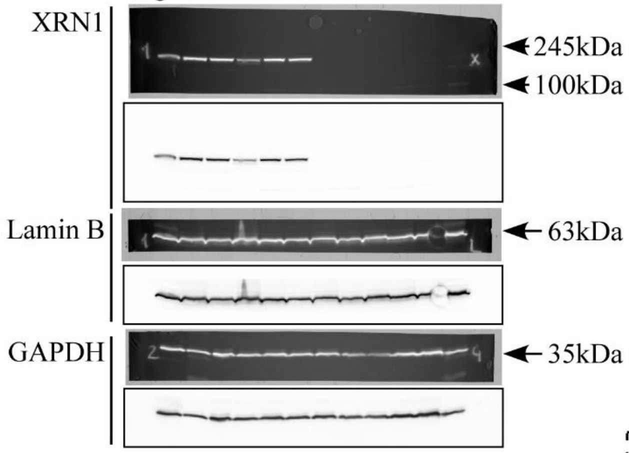

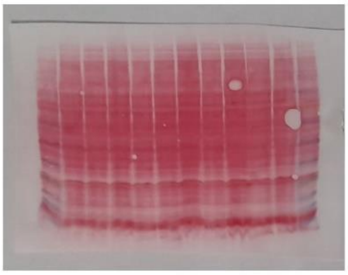

Figure 1H

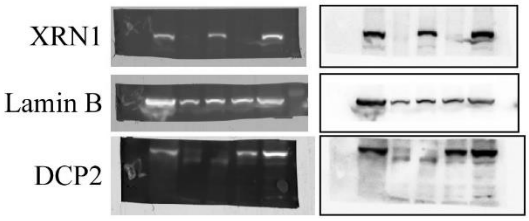

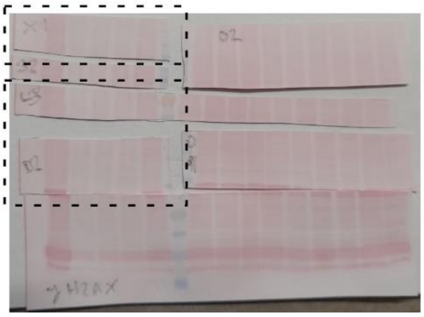

Figure 2A

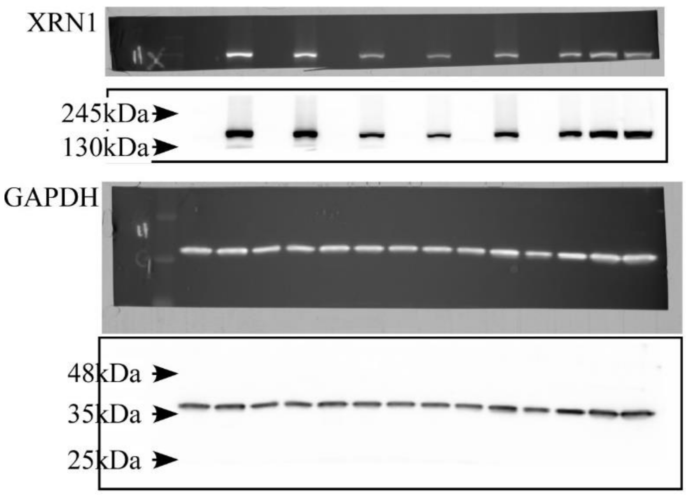

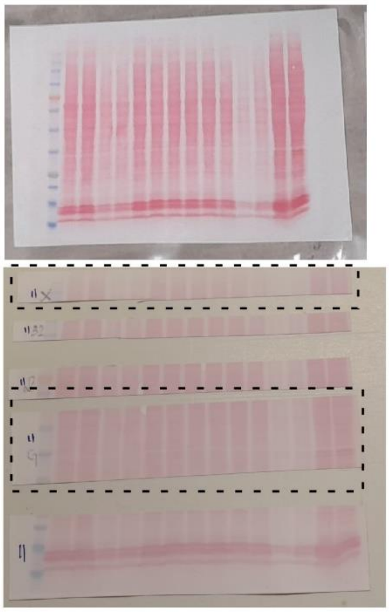

Figures 2E

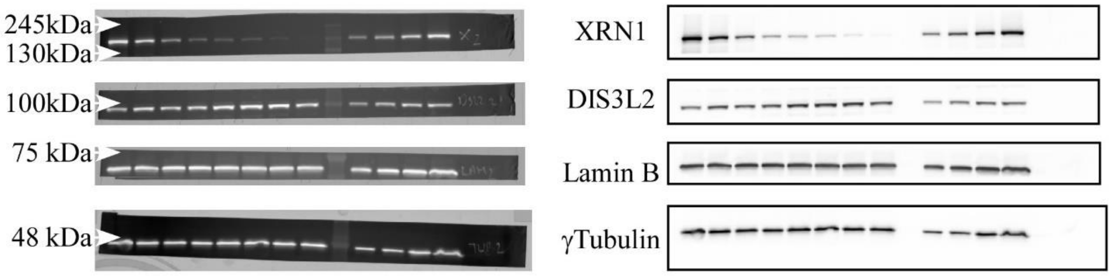

Figure 2F

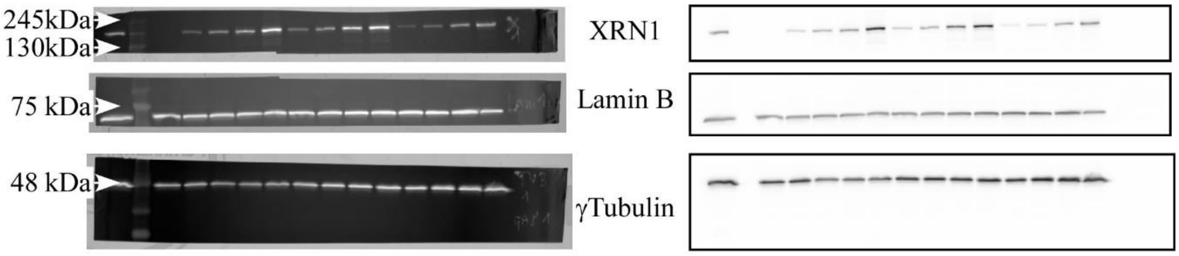

Figures 2E & 2F

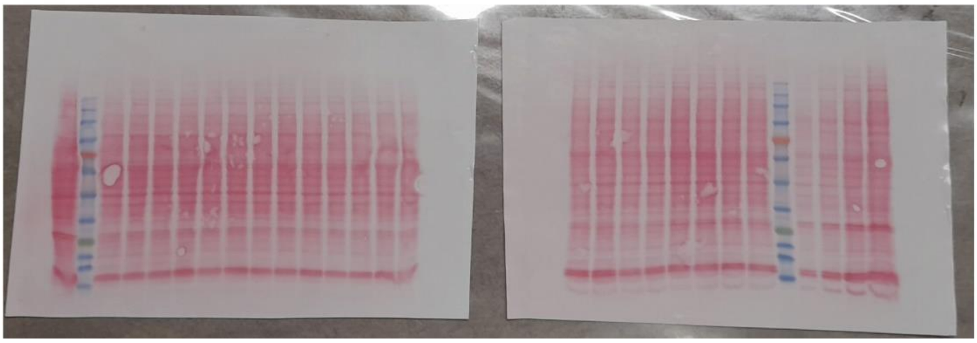

Figure 3B

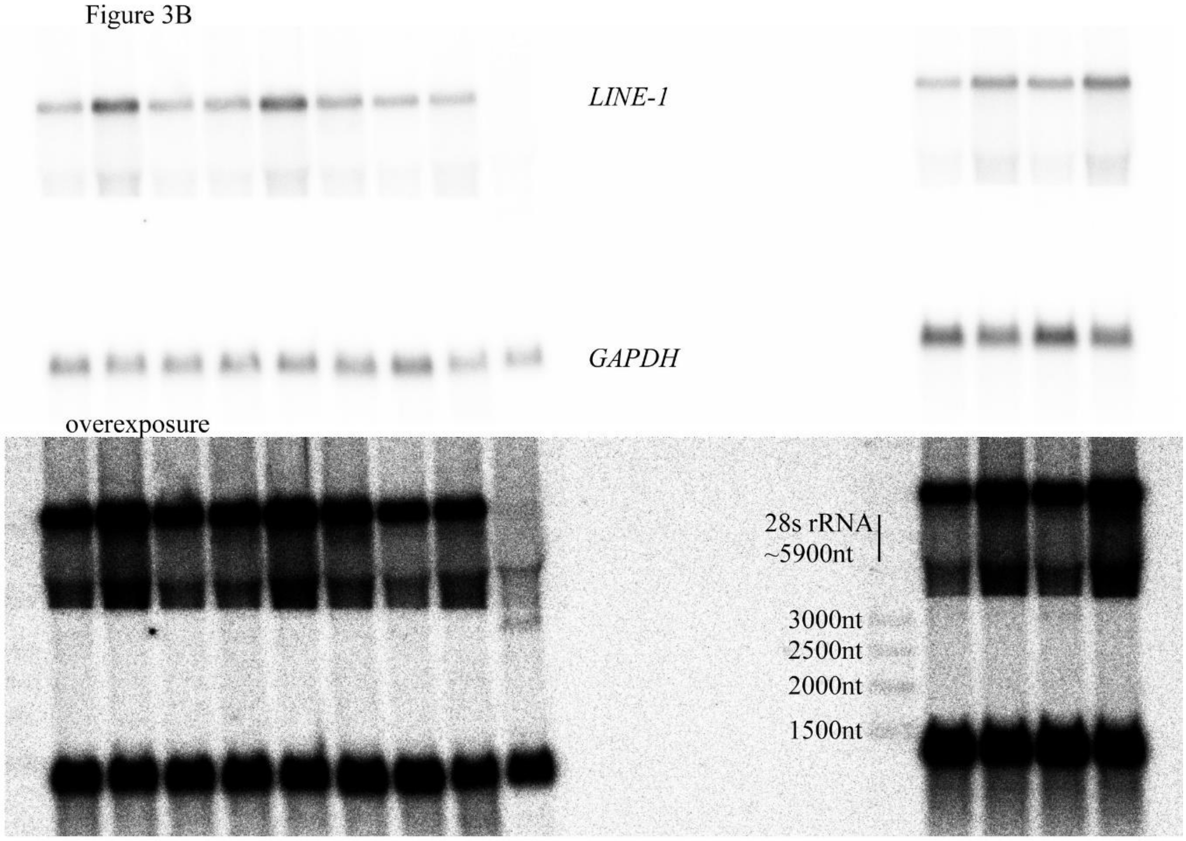

Figure 3H

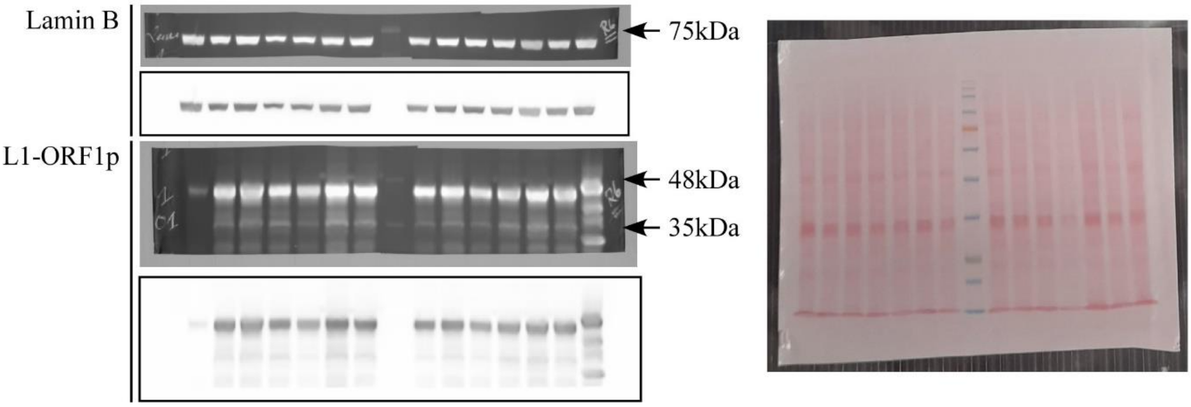

Figure 4F

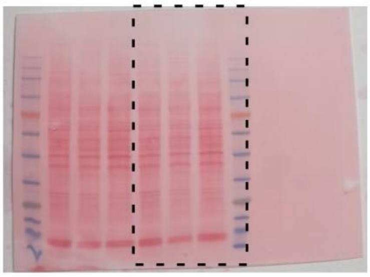

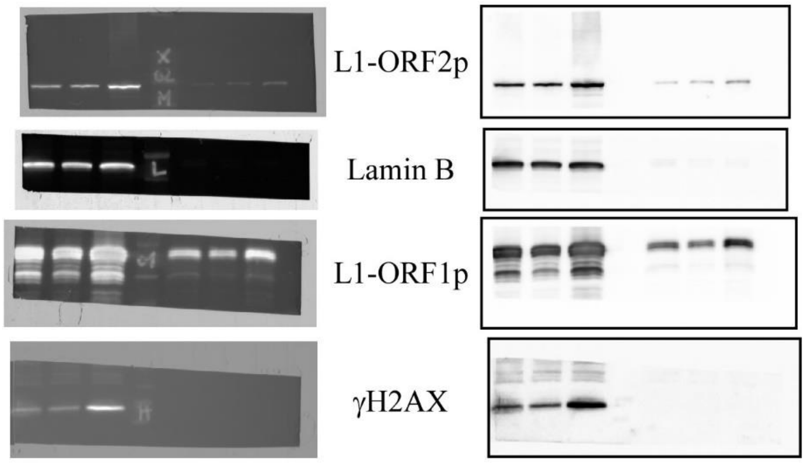

Figure 4G

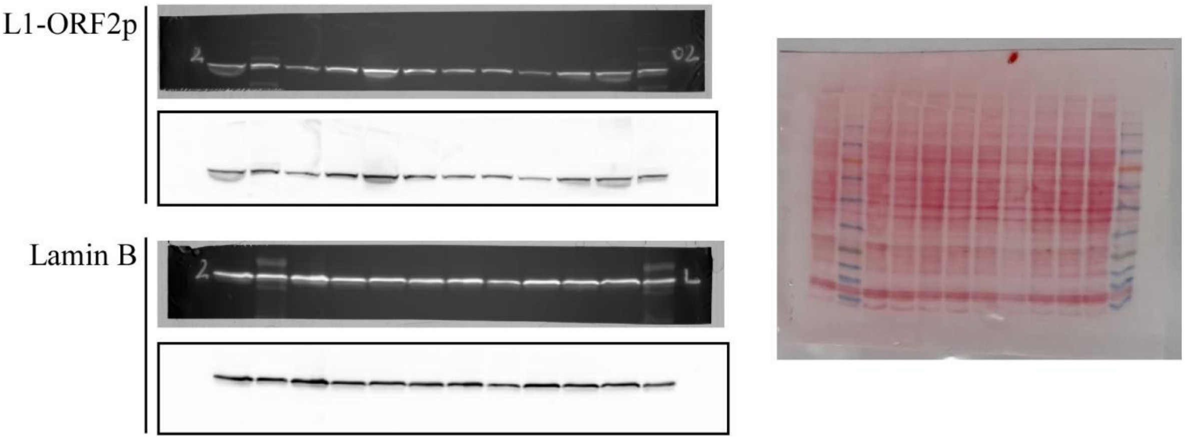

Figure 7C

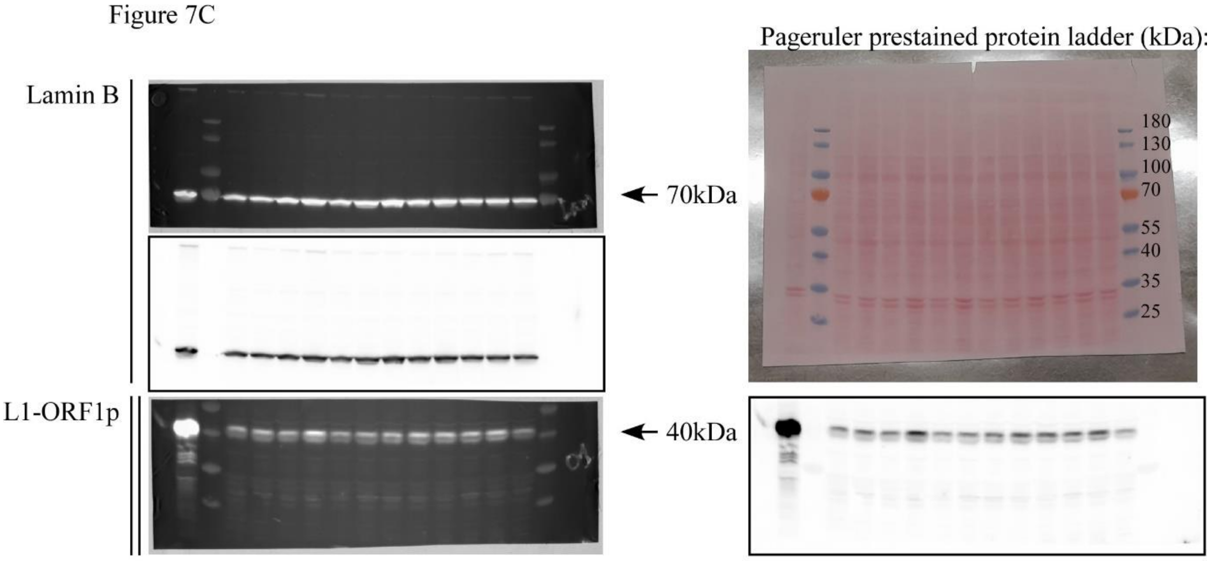

Figure 7

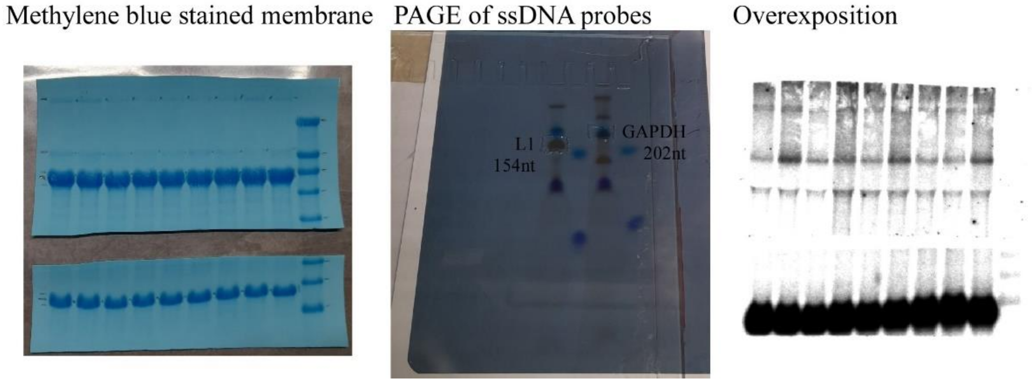

Supplementary Fig 2G

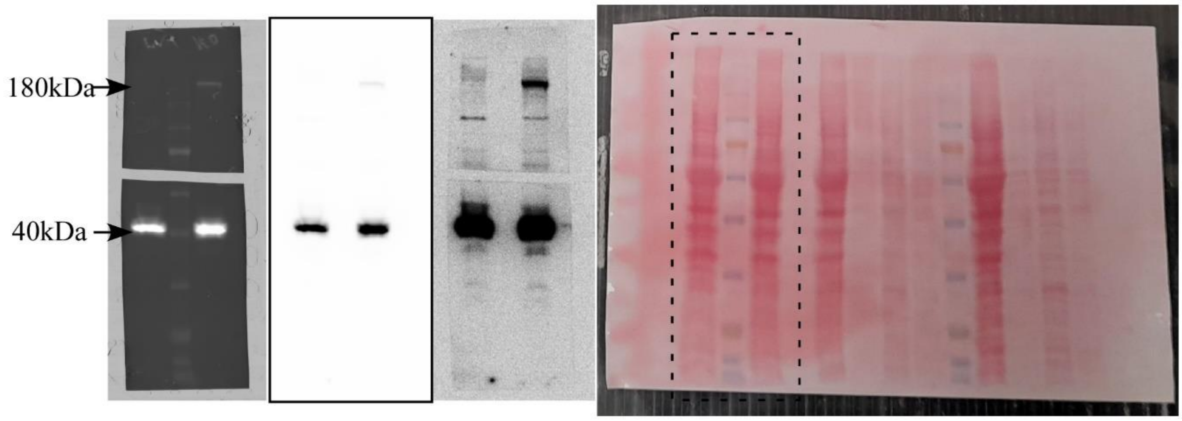

Supplementary Fig 3F

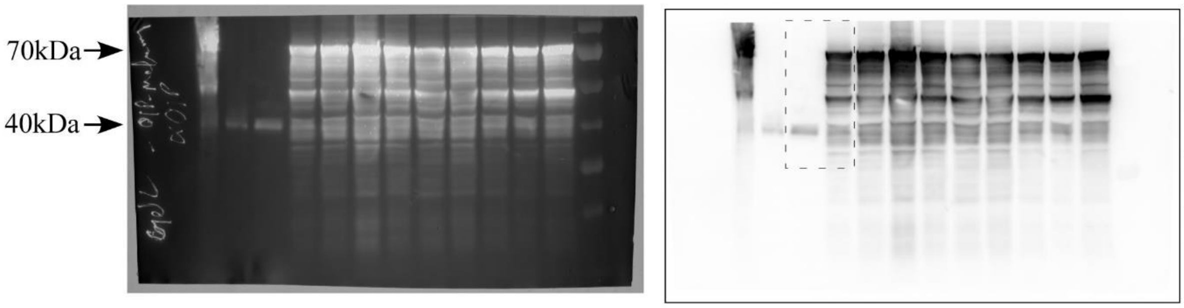

Supplementary Fig 3G

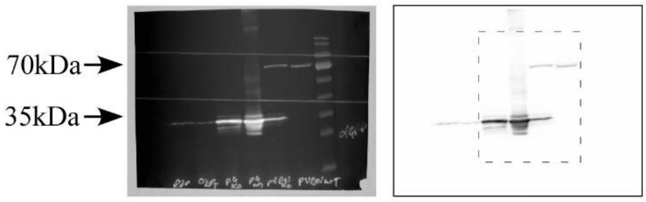

Two protein ladders were used in the study (name and bands in kDa, ~):
PAGE Ruler prestained (ThermoScientific)
180, 130, 100, 70, 55, 40, 35, 25, 15, 10
EurX Perfect Tricolor (EurX)
245, 180, 135, 100, 75, 63, 48, 35, 25, 20, 17, 10

Supplementary Fig 3H

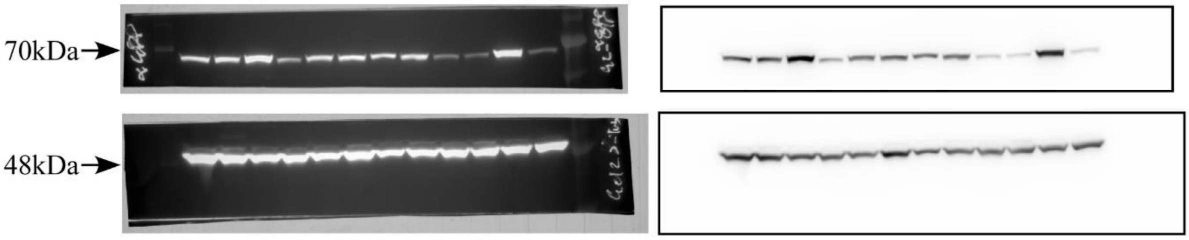

Supplementary Fig 3I

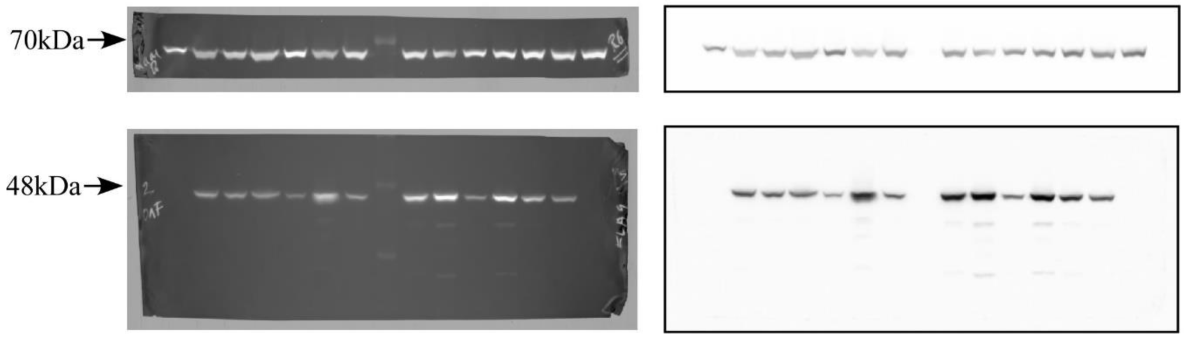

Supplementary Fig 4H

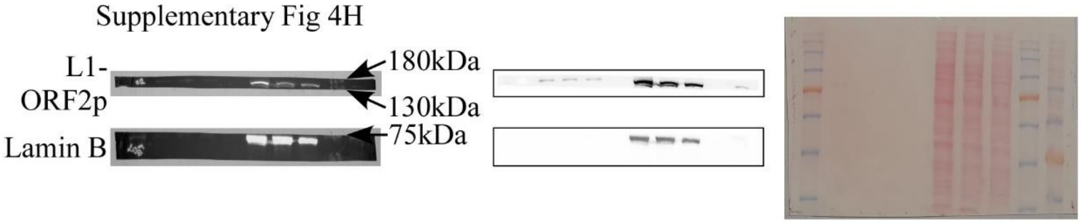

Supplementary Fig 4I

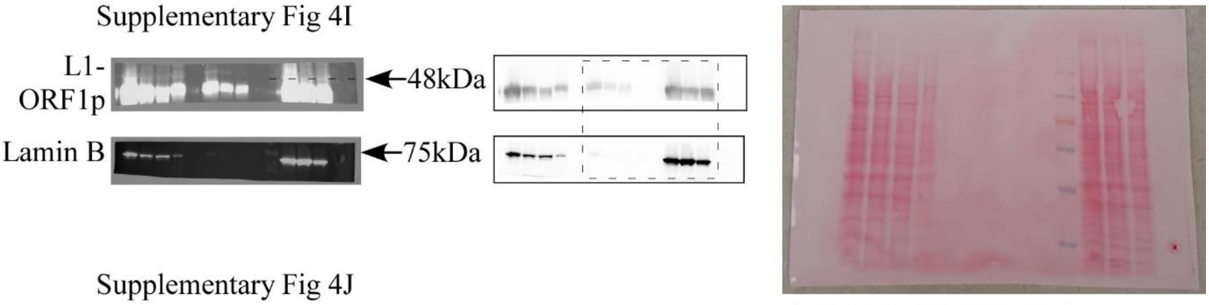

Supplementary Fig 4J

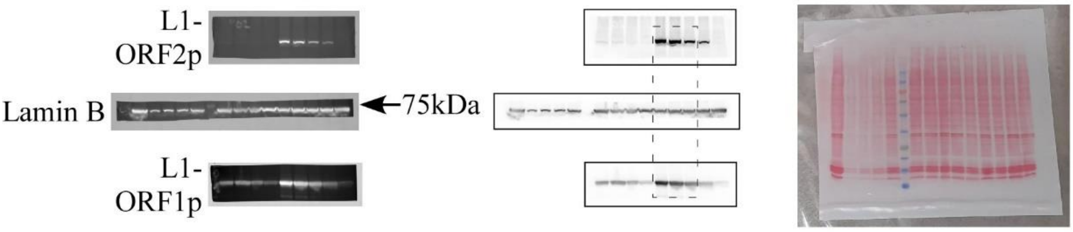

Supplementary Fig 4L

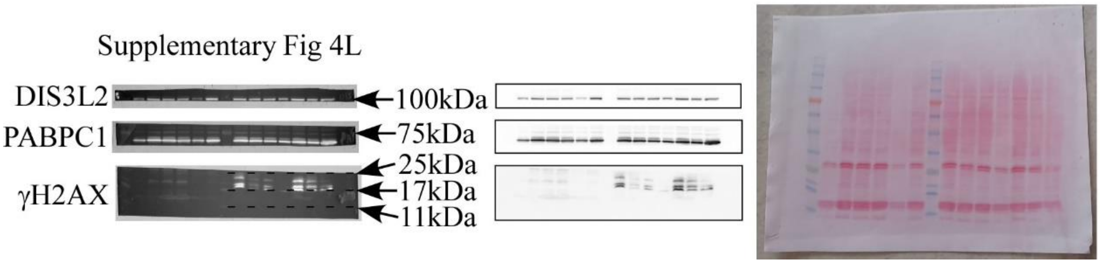

Supplementary Fig 6A

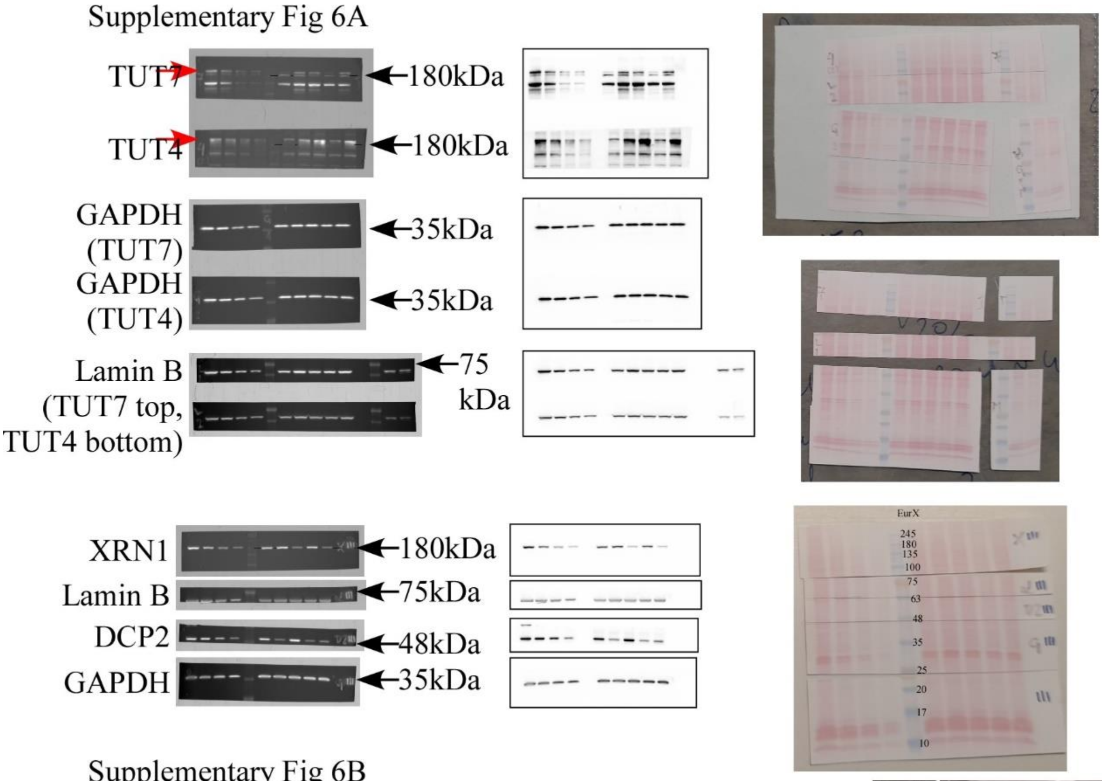

Supplementary Fig 6B

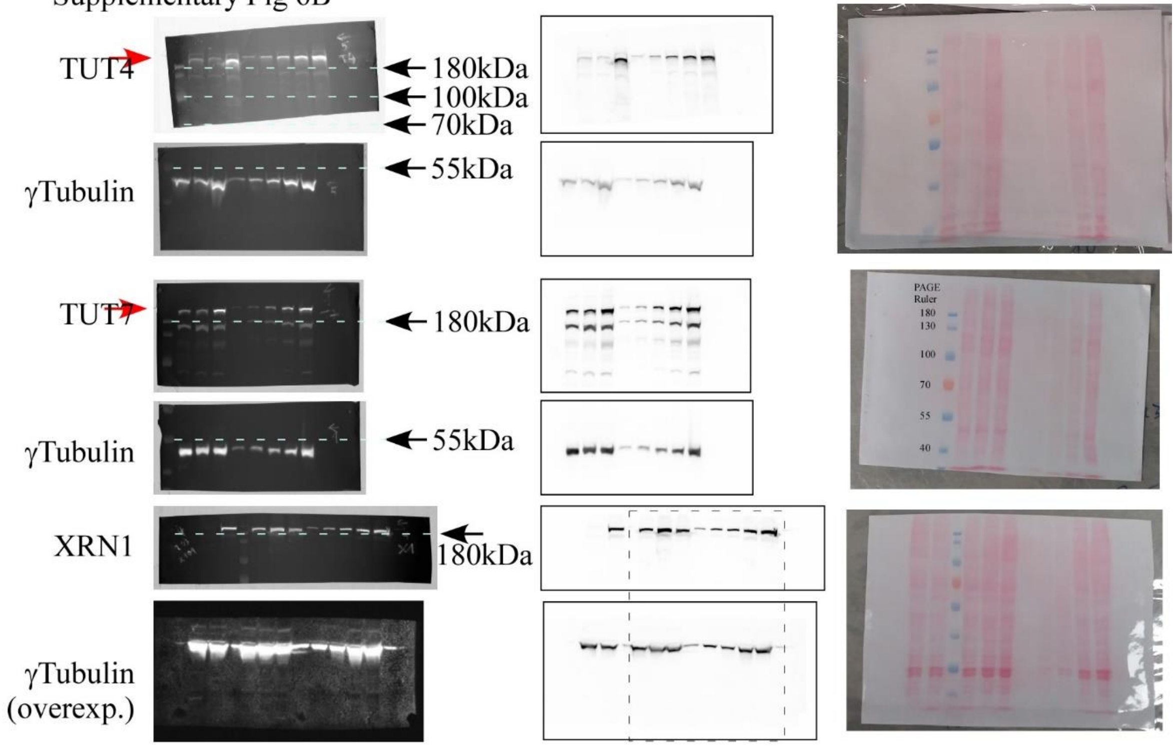

Supplementary Fig 6F

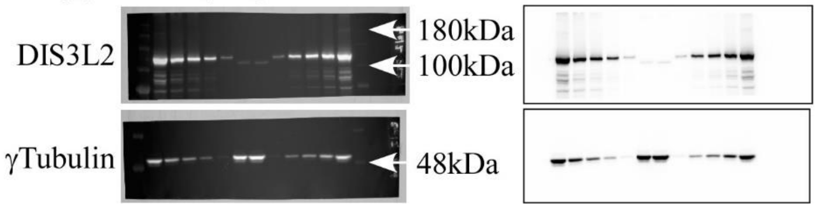

Supplementary Fig 7D

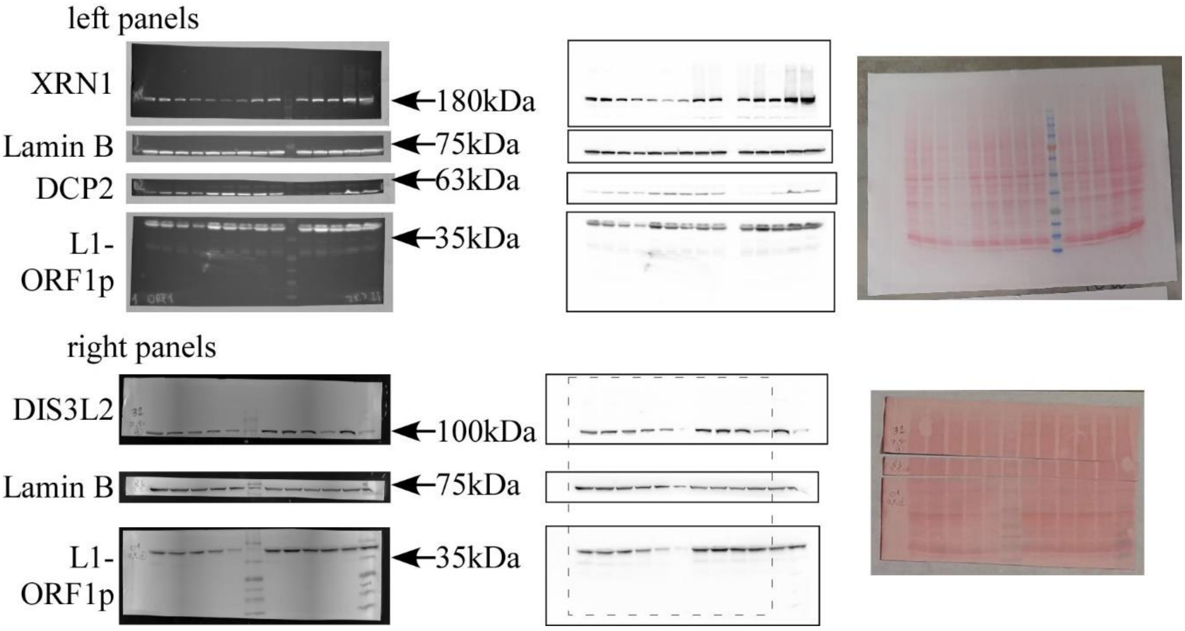

The STR profiles of the cell lines used and created in this study

293T, HeLa ‘Ha’, and PA-1 cell lines were kind gifts of dr Jose L. Garcia-Perez (Edinburgh). The cell lines were authenticated by STR profiling of 16 STR traits and compared to the STR profiles of the reference cell lines using CellDive (15). HeLa and PA-1 100% matched their respective references, and did not have any additional STR lengths in the STR profiles. The 293T cell line used to generate the clonal *XRN1* KO cell lines also 100% matched a 293T reference. However, we observed some additional STRs in this 293T. Thus we generated STR profiles for all the clonal cell lines derived in this study. All of the clonal wild-type and *XRN1* KO 293T cell lines of this study matched the authentication criteria by falling within the window of 100-80% similarity with the reference 293T STR profiles. Thus we conclude that the additional STRs in the parental 293T are not a result of a contamination with a different cell line. Browsing through the different ‘293T’ entries in the DSMZ database also revealed variability in their STR profiles and revealed additional alleles in many of the deposited cell lines. We thus conclude that the STR variability is generally present in the different ‘293T’ cell lines.

The reports comprise original STR chromatograms and screenshots of the CellDive browsing using the STR profiles from the chromatograms. In the parental 293T cell line, a reference 293T STR profile is also shown.

293T

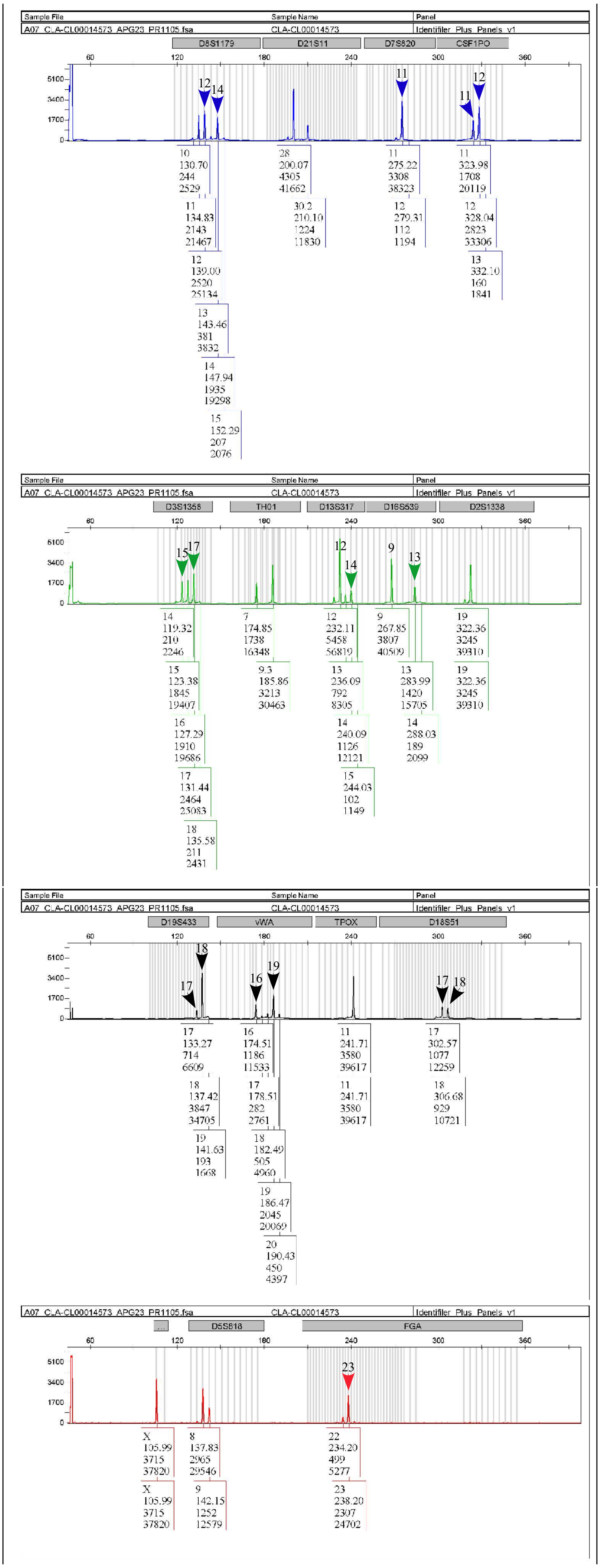

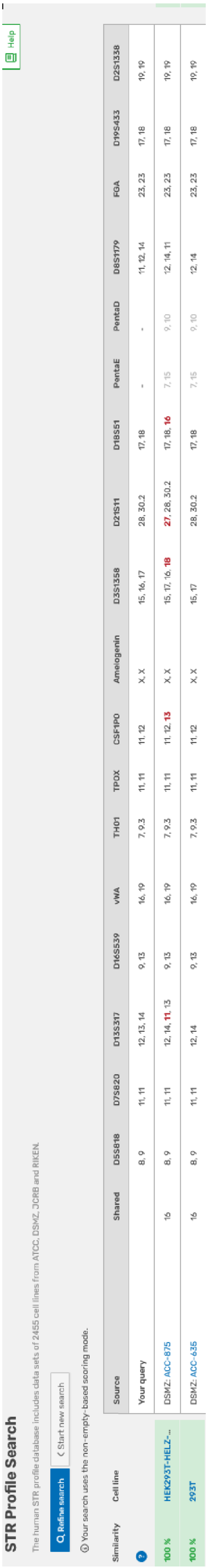

STR Profile Search

The human STR profile database includes data sets of 2455 cell lines from ATCC, DSMZ, JCRB and RIKEN.

Refine search | Start new search

Your search uses the non-empty-based scoring mode.

Similarity	Cell line	Source	Shared	D5S818	D7S820	D13S317	D16S539	vWA	TH01	TPOX	CSF1PO	Amelogenin	D3S1358	D21S11	D18S51	PentaE	PentaD	D8S1179	FGA	D19S433	D2S1338
		Your query		8, 9	11, 11	12, 13, 14	9, 13	16, 19	7, 9.3	11, 11	11, 12	X, X	15, 16, 17	28, 30.2	17, 18	-	-	11, 12, 14	23, 23	17, 18	19, 19
100 %	HEK293T-HELZ-...	DSMZ: ACC-875	16	8, 9	11, 11	12, 14, 11, 13	9, 13	16, 19	7, 9.3	11, 11	11, 12, 13	X, X	15, 17, 16, 18	27, 28, 30.2	17, 18, 16	7, 15	9, 10	12, 14, 11	23, 23	17, 18	19, 19
100 %	293T	DSMZ: ACC-635	16	8, 9	11, 11	12, 14	9, 13	16, 19	7, 9.3	11, 11	11, 12	X, X	15, 17	28, 30.2	17, 18	7, 15	9, 10	12, 14	23, 23	17, 18	19, 19

293T 'B1'

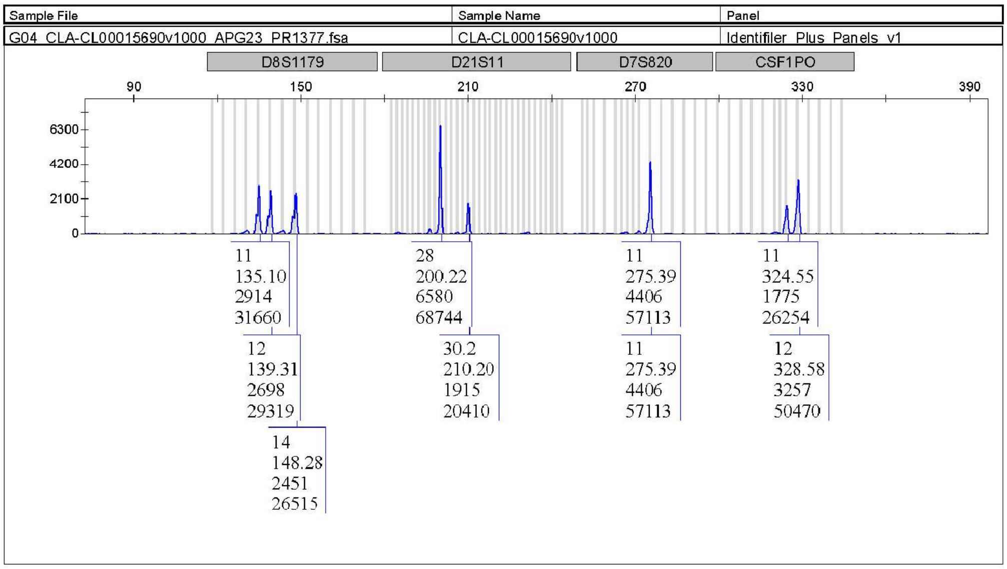

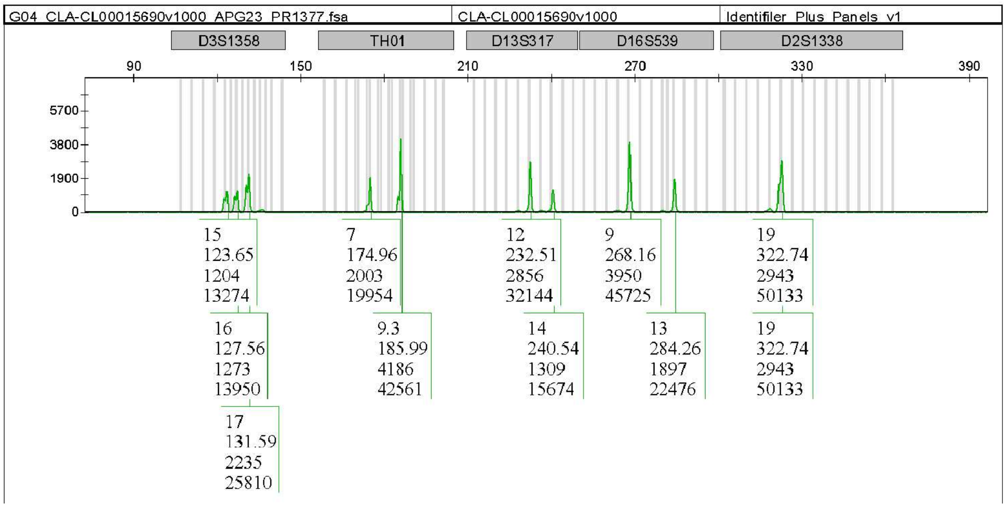

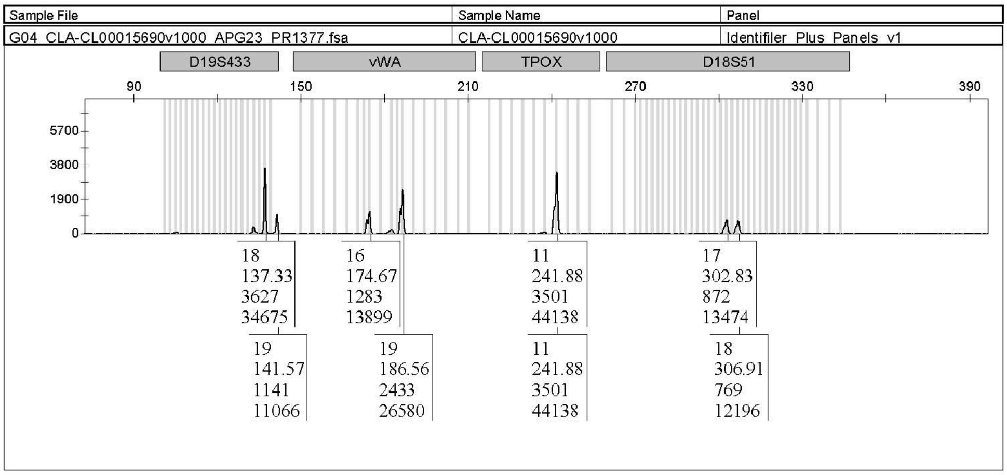

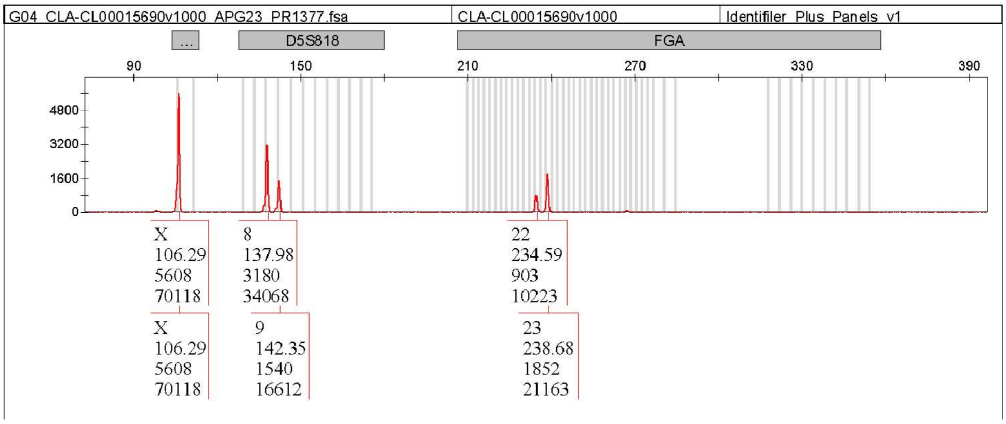

Similarity	Cell line	Source	Shared	D5S818	D7S820	D13S317	D16S539	vWA	TH01	TPOX	CSF1PO	Amelogenin	D3S1358	D21S11	D18S51	PentaE	PentaD	D8S1179	FGA	D19S433	D2S1338
		Your query		8, 9	11, 11	12, 14	9, 13	16, 19	7, 9.3	11, 11	11, 12	X, X	15, 16, 17	28, 30.2	17, 18	-	-	11, 12, 14	22, 23	18, 19	19, 19
94.1 %	HEK293T-HELZ-...	DSMZ: ACC-875	16	8, 9	11, 11	12, 14, 11, 13	9, 13	16, 19	7, 9.3	11, 11	11, 12, 13	X, X	15, 17, 16, 18	27, 28, 30.2	17, 18, 16	7, 15	9, 10	12, 14, 11	23, 23	17, 18	19, 19
93.9 %	ANJOU 65	CRL-11269	16	8, 9	11, 11	11, 12, 14	9, 13	16, 18, 19	7, 9.3	11, 11	11, 12	X, X	15, 17	28, 30.2	17, 18, 19	7, 15, 14	9, 10	12, 14, 11, 15	20, 23	17, 18	19, 19
93.9 %	293T/17 [HEK 29...	CRL-11268	16	8, 9	11, 11	12, 14	9, 13	16, 18, 19	7, 9.3	11, 11	11, 12	X, X	15, 17	28, 30.2	17, 18, 19	7, 15, 14	9, 10	12, 14, 11, 15	20, 23	17, 18	19, 19
93.9 %	HEK 293T/17	ACS-4500	16	8, 9	11, 11	12, 14	9, 13	16, 19	7, 9.3	11, 11	11, 12	X, X	15, 17	28, 30.2	17, 18, 19	7, 15, 14	9, 10	12, 14, 11, 15	20, 23	17, 18	19, 19
93.9 %	HEK293T-CAF4...	DSMZ: ACC-872	16	8, 9	11, 11	12, 14	9, 13	16, 18, 20	7, 9.3	11, 11	11, 12	X, X	15, 17	28, 30.2	17, 18	7, 15	9, 10	11, 12, 14	23, 23	17, 18, 19	19, 19
93.8 %	293T	DSMZ: ACC-635	16	8, 9	11, 11	12, 14	9, 13	16, 19	7, 9.3	11, 11	11, 12	X, X	15, 17	28, 30.2	17, 18	7, 15	9, 10	12, 14	23, 23	17, 18	19, 19

293T 'B5'

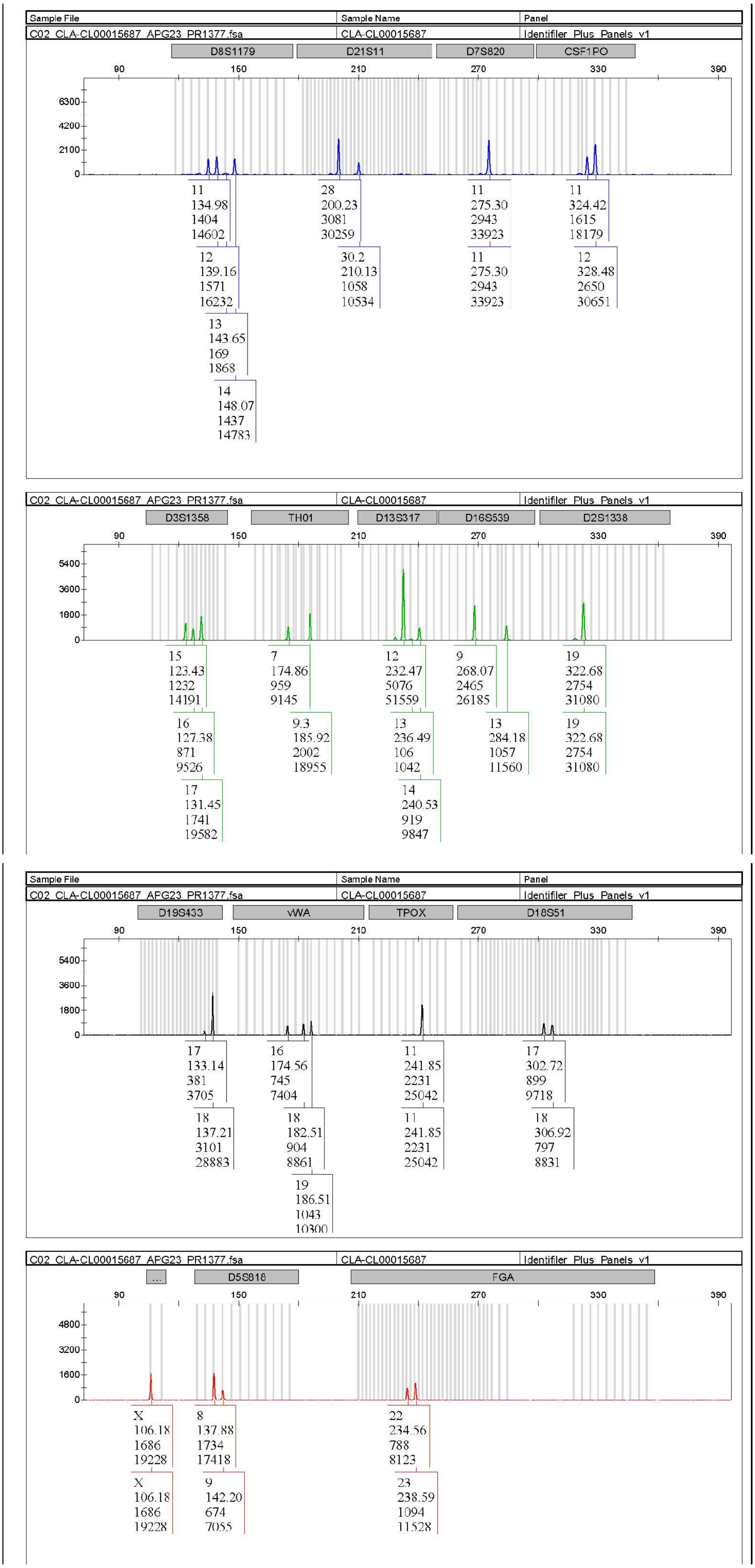

Similarity	Cell line	Source	Shared	D5S818	D7S820	D13S317	D16S539	vWA	TH01	TPOX	CSF1PO	Amelogenin	D3S1358	D21S11	D18S51	PentaE	PentaD	D8S1179	FGA	D19S433	D2S1338
		Your query		8, 9	11, 11	12, 14	9, 13	16, 18, 19	7, 9.3	11, 11	11, 12	X, X	15, 16, 17	28, 30.2	17, 18	-	-	11, 12, 14	22, 23	17, 18	19, 19
97.1 %	ANJOU 65	CRL-11269	16	8, 9	11, 11	11, 12, 14	9, 13	16, 18, 19	7, 9.3	11, 11	11, 12	X, X	15, 17	28, 30.2	17, 18, 19	7, 15, 14	9, 10	12, 14, 11, 15	20, 23	17, 18	19, 19
97.1 %	293T/17 [HEK 29...	CRL-11268	16	8, 9	11, 11	12, 14	9, 13	16, 18, 19	7, 9.3	11, 11	11, 12	X, X	15, 17	28, 30.2	17, 18, 19	7, 15, 14	9, 10	12, 14, 11, 15	20, 23	17, 18	19, 19
97.1 %	HEK293T-HELZ-...	DSMZ: ACC-875	16	8, 9	11, 11	12, 14, 11, 13	9, 13	16, 19	7, 9.3	11, 11	11, 12, 13	X, X	15, 17, 16, 18	27, 28, 30.2	17, 18, 16	7, 15	9, 10	12, 14, 11	23, 23	17, 18	19, 19
97 %	HEK 293T/17	ACS-4500	16	8, 9	11, 11	12, 14	9, 13	16, 19	7, 9.3	11, 11	11, 12	X, X	15, 17	28, 30.2	17, 18, 19	7, 15, 14	9, 10	12, 14, 11, 15	20, 23	17, 18	19, 19
96.9 %	293T	DSMZ: ACC-635	16	8, 9	11, 11	12, 14	9, 13	16, 19	7, 9.3	11, 11	11, 12	X, X	15, 17	28, 30.2	17, 18	7, 15	9, 10	12, 14	23, 23	17, 18	19, 19

293T 'B7'

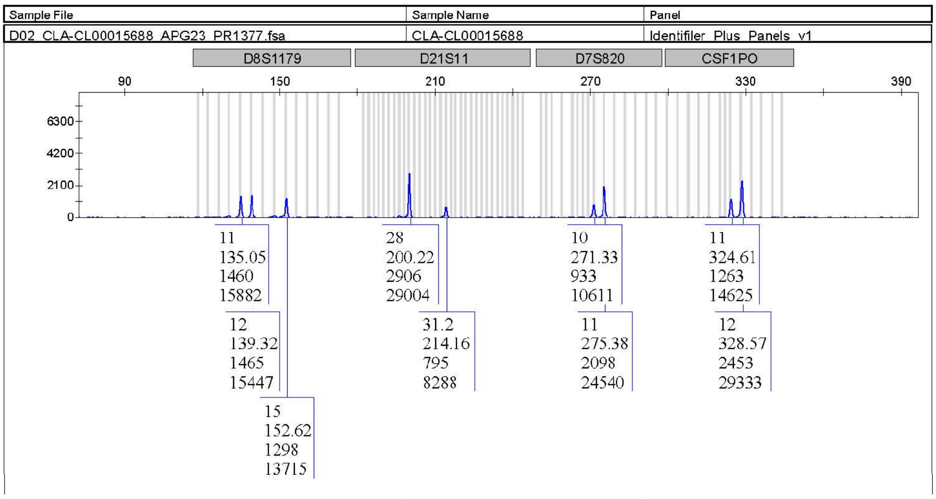

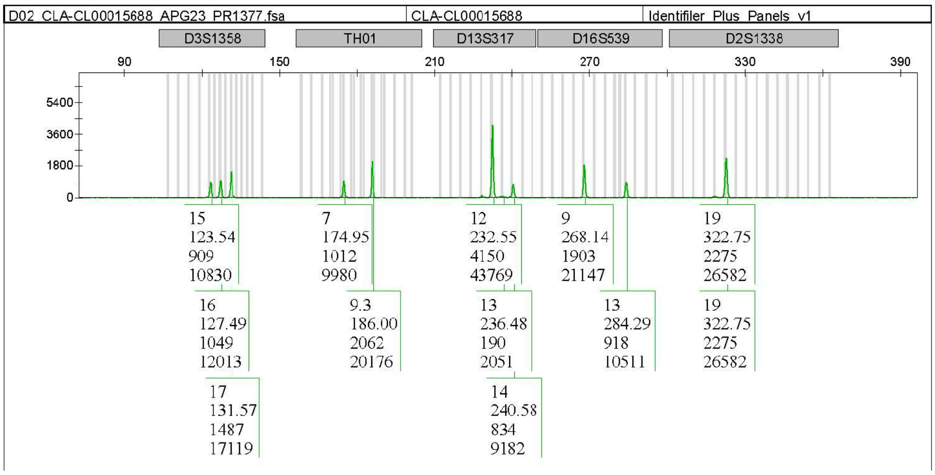

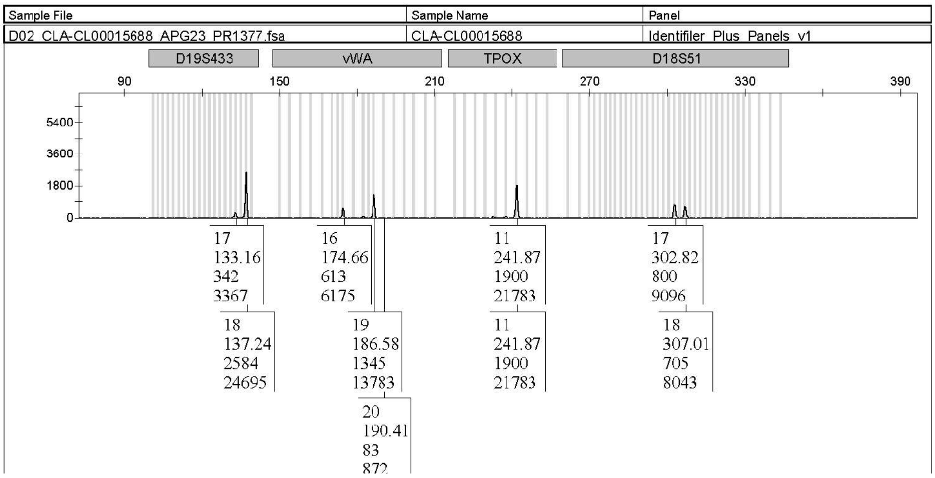

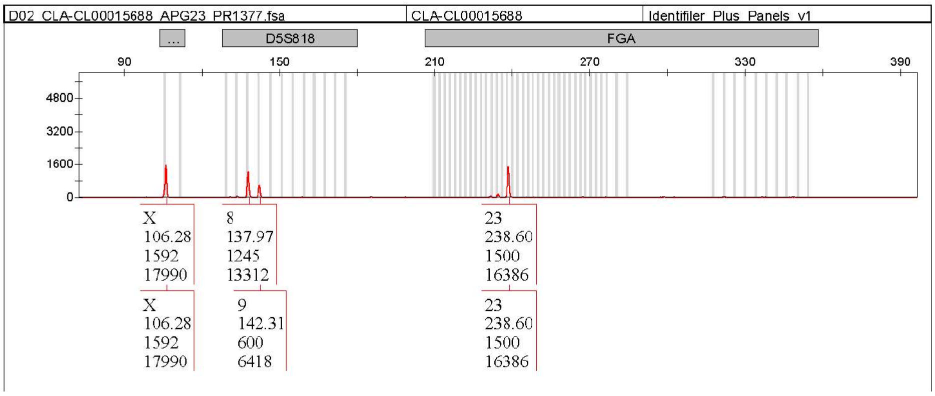

Similarity	Cell line	Source	Shared	D5S818	D7S820	D13S317	D16S539	vWA	TH01	TPOX	CSF1PO	Amelogenin	D3S1358	D21S11	D18S51	PentaE	PentaD	D8S1179	FGA	D19S433	D2S1338
		Your query		8, 9	10, 11	12, 14	9, 13	16, 19	7, 9.3	11, 11	11, 12	X, X	15, 16, 17	28, 31.2	17, 18	-	-	11, 12, 15	23, 23	17, 18	19, 19
91.2 %	HEK293T-HELZ-...	DSMZ: ACC-875	16	8, 9	11, 11	12, 14, 11, 13	9, 13	16, 19	7, 9.3	11, 11	11, 12, 13	X, X	15, 17, 16, 18	27, 28, 30.2	17, 18, 16	7, 15	9, 10	12, 14, 11	23, 23	17, 18	19, 19
90.9 %	ANJOU 65	CRL-11269	16	8, 9	11, 11	11, 12, 14	9, 13	16, 18, 19	7, 9.3	11, 11	11, 12	X, X	15, 17	28, 30.2	17, 18, 19	7, 15, 14	9, 10	12, 14, 11, 15	20, 23	17, 18	19, 19
90.9 %	293T/17 [HEK 29...	CRL-11268	16	8, 9	11, 11	12, 14	9, 13	16, 18, 19	7, 9.3	11, 11	11, 12	X, X	15, 17	28, 30.2	17, 18, 19	7, 15, 14	9, 10	12, 14, 11, 15	20, 23	17, 18	19, 19
90.9 %	HEK 293T/17	ACS-4500	16	8, 9	11, 11	12, 14	9, 13	16, 19	7, 9.3	11, 11	11, 12	X, X	15, 17	28, 30.2	17, 18, 19	7, 15, 14	9, 10	12, 14, 11, 15	20, 23	17, 18	19, 19
90.6 %	293T	DSMZ: ACC-635	16	8, 9	11, 11	12, 14	9, 13	16, 19	7, 9.3	11, 11	11, 12	X, X	15, 17	28, 30.2	17, 18	7, 15	9, 10	12, 14	23, 23	17, 18	19, 19

293T ‘B11'

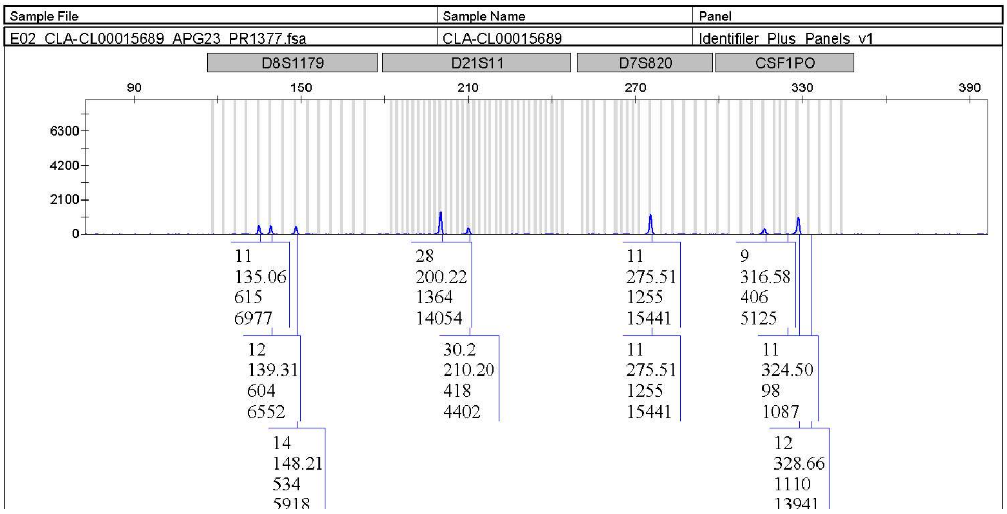

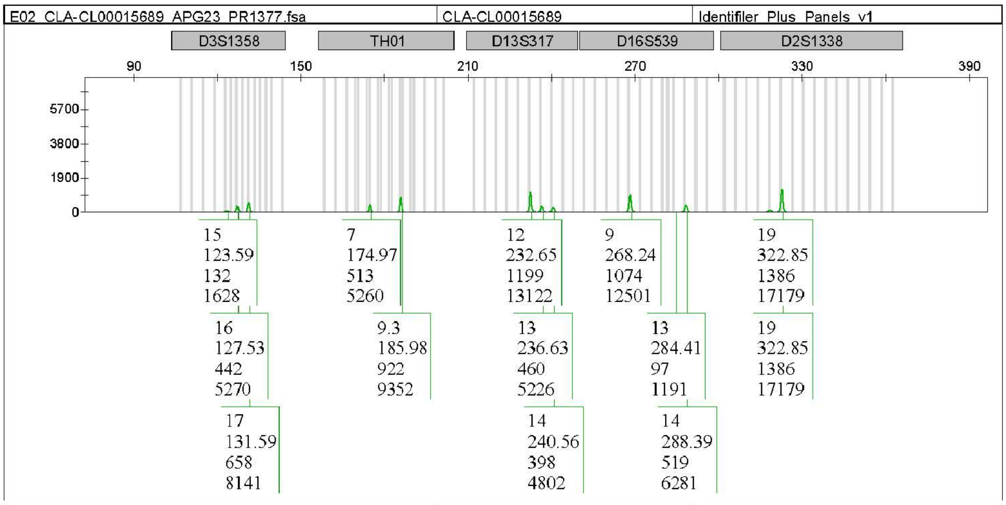

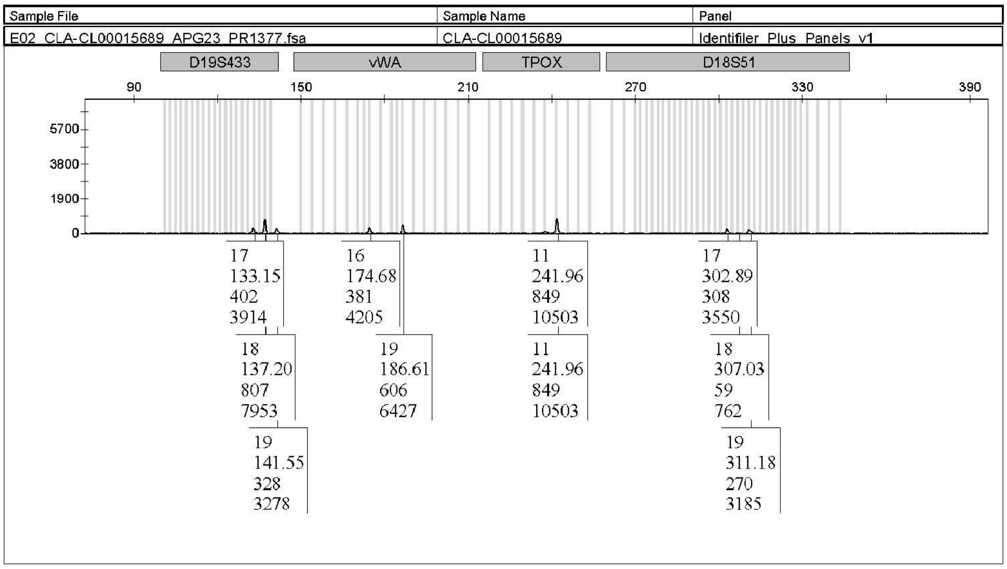

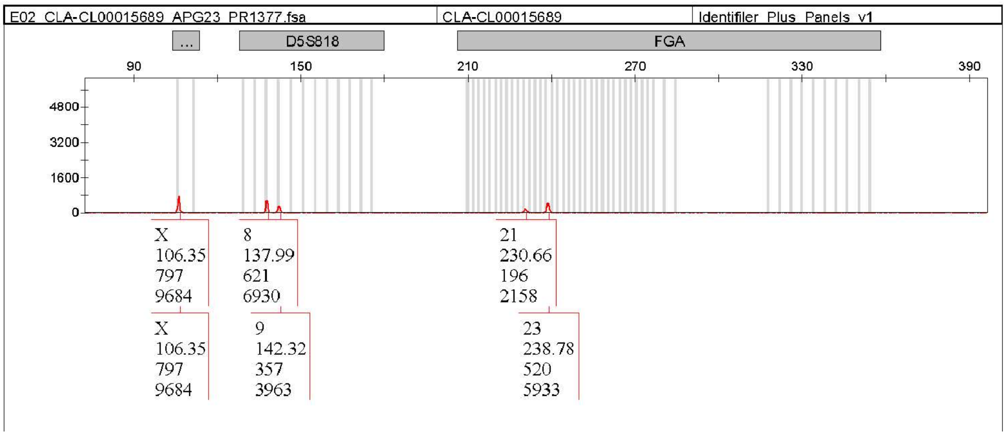

Similarity	Cell line	Source	Shared	D5S818	D7S820	D13S317	D16S539	vWA	TH01	TPOX	CSF1PO	Amelogenin	D3S1358	D21S11	D18S51	PentaE	PentaD	D8S1179	FGA	D19S433	D2S1338
		Your query		8, 9	11, 11	12, 13, 14	9, 13	16, 19	7, 9.3	11, 11	9, 12	X, X	15, 16, 17	28, 30.2	17, 19	-	-	11, 12, 13	21, 23	17, 18, 19	19, 19
90.9 %	293T/17 [HEK 29...	CRL-11268	16	8, 9	11, 11	12, 14	9, 13	16, 18, 19	7, 9.3	11, 11	11, 12	X, X	15, 17	28, 30.2	17, 18, 19	7, 15, 14	9, 10	12, 14, 11, 15	20, 23	17, 18	19, 19
90.9 %	HEK 293T/17	ACS-4500	16	8, 9	11, 11	12, 14	9, 13	16, 19	7, 9.3	11, 11	11, 12	X, X	15, 17	28, 30.2	17, 18, 19	7, 15, 14	9, 10	12, 14, 11, 15	20, 23	17, 18	19, 19
88.6 %	HEK293T-HELZ-...	DSMZ: ACC-875	16	8, 9	11, 11	12, 14, 11, 13	9, 13	16, 19	7, 9.3	11, 11	11, 12, 13	X, X	15, 17, 16, 18	27, 28, 30.2	17, 18, 16	7, 15	9, 10	12, 14, 11	23, 23	17, 18	19, 19
88.2 %	ANJOU 65	CRL-11269	16	8, 9	11, 11	11, 12, 14	9, 13	16, 18, 19	7, 9.3	11, 11	11, 12	X, X	15, 17	28, 30.2	17, 18, 19	7, 15, 14	9, 10	12, 14, 11, 15	20, 23	17, 18	19, 19
87.5 %	293T	DSMZ: ACC-635	16	8, 9	11, 11	12, 14	9, 13	16, 19	7, 9.3	11, 11	11, 12	X, X	15, 17	28, 30.2	17, 18	7, 15	9, 10	12, 14	23, 23	17, 18	19, 19

293T 'B17'

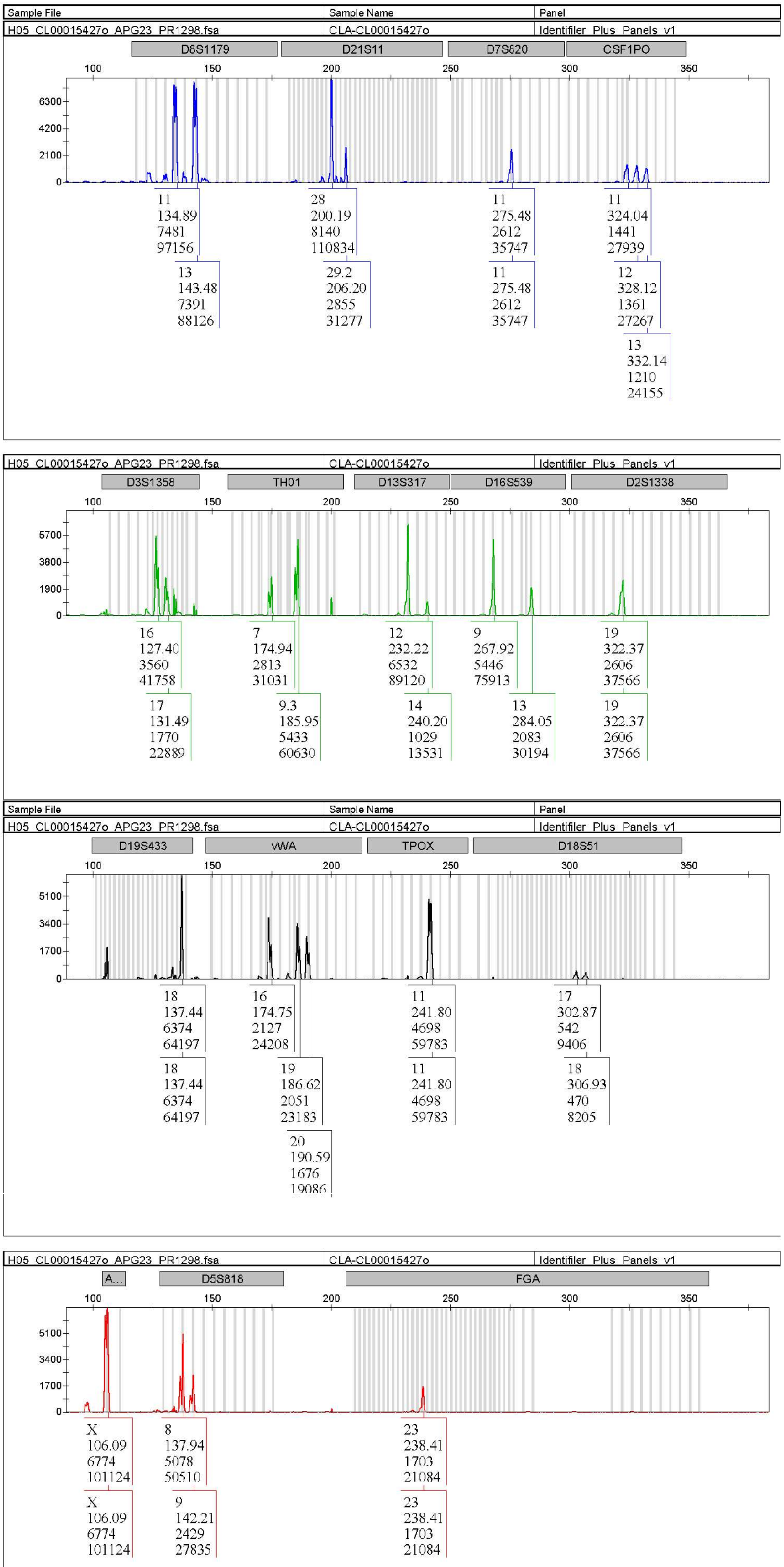

STR Profile Search

The human STR profile database includes data sets of 2455 cell lines from ATCC, DSMZ, JCRB and RIKEN.

Refine search | Start new search | Help

Your search uses the non-empty-based scoring mode.

Similarity	Cell line	Source	Shared	D5S818	D7S820	D13S317	D16S539	vWA	TH01	TPOX	CSF1PO	Amelogenin	D3S1358	D21S11	D18S51	PentaE	PentaD	D8S1179	FGA	D19S433	D2S1338
?		Your query		8, 9	11, 11	12, 14	9, 13	16, 19, 20	7, 9.3	11, 11	11, 12, 13	X, X	16, 17	28, 29.2	17, 18	-	-	11, 13	23, 23	18, 18	19, 19
90.9 %	HEK293T-HELZ-...	DSMZ: ACC-875	16	8, 9	11, 11	12, 14, **11**, **13**	9, 13	16, 19	7, 9.3	11, 11	11, 12, 13	X, X	**15**, 17, 16, **18**	**27**, 28, **30.2**	17, 18, **16**	7, 15	9, 10	**12**, **14**, 11	23, 23	**17**, 18	19, 19
84.8 %	HEK293T-CAF4...	DSMZ: ACC-872	16	8, 9	11, 11	12, 14	9, 13	16, **18**, 20	7, 9.3	11, 11	11, 12	X, X	**15**, 17	28, **30.2**	17, 18	7, 15	9, 10	11, **12**, **14**	23, 23	**17**, 18, **19**	19, 19
84.4 %	293T	RCB2202	16	8, 9	11, 11	12, 14	9, 13	16, 19	7, 9.3	11, 11	11, 12	X, X	**15**, 17	28, **30.2**	18, **18**	7, 15	9, 10	**12**, **14**	23, 23	18, 18	19, 19
84.4 %	HEK 293T/17	ACS-4500	16	8, 9	11, 11	12, 14	9, 13	16, 19	7, 9.3	11, 11	11, 12	X, X	**15**, 17	28, **30.2**	17, 18, **19**	7, 15, 14	9, 10	**12**, **14**, 11, **15**	**20**, 23	**17**, 18	19, 19
84.4 %	293T	DSMZ: ACC-635	16	8, 9	11, 11	12, 14	9, 13	16, 19	7, 9.3	11, 11	11, 12	X, X	**15**, 17	28, **30.2**	17, 18	7, 15	9, 10	**12**, **14**	23, 23	**17**, 18	19, 19

293T 'B3'

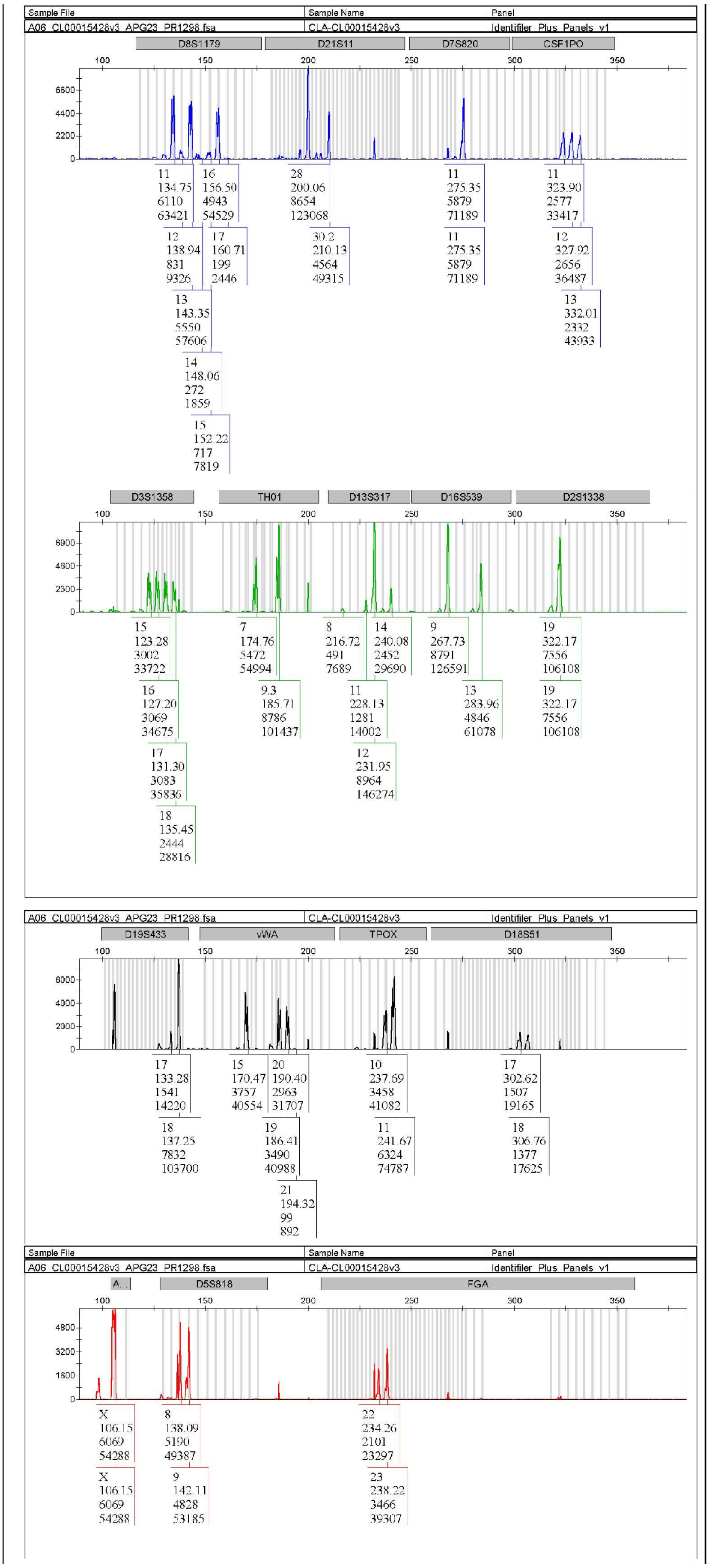

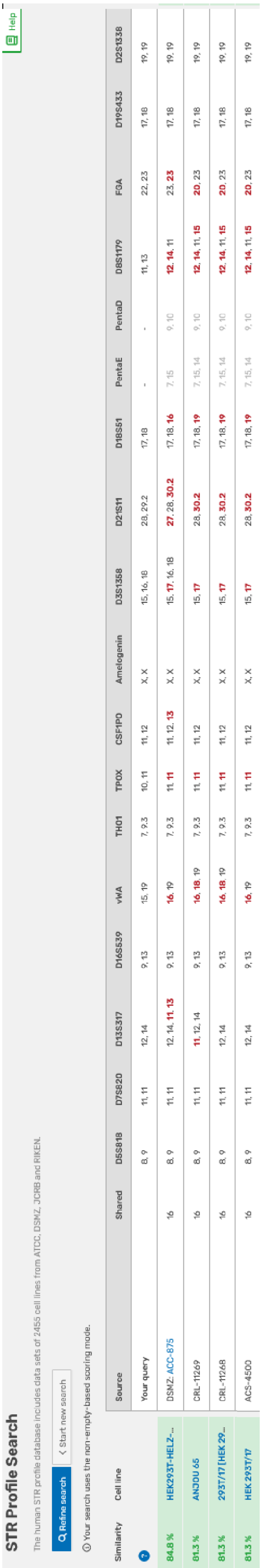

Similarity	Cell line	Source	Shared	D5S818	D7S820	D13S317	D16S539	vWA	TH01	TPOX	CSF1PO	Amelogenin	D3S1358	D21S11	D18S51	PentaE	PentaD	D8S1179	FGA	D19S433	D2S1338
?		Your query		8, 9	11, 11	12, 14	9, 13	15, 19	7, 9.3	10, 11	11, 12	X, X	15, 16, 18	28, 29.2	17, 18	-	-	11, 13	22, 23	17, 18	19, 19
84.8 %	HEK293T-HEL2-...	DSMZ: ACC-875	16	8, 9	11, 11	12, 14, 11, 13	9, 13	16, 19	7, 9.3	11, 11	11, 12, 13	X, X	15, 17, 16, 18	27, 28, 30.2	17, 18, 16	7, 15	9, 10	12, 14, 11	23, 23	17, 18	19, 19
81.3 %	ANJOU 65	CRL-11269	16	8, 9	11, 11	11, 12, 14	9, 13	16, 18, 19	7, 9.3	11, 11	11, 12	X, X	15, 17	28, 30.2	17, 18, 19	7, 15, 14	9, 10	12, 14, 11, 15	20, 23	17, 18	19, 19
81.3 %	293T/17 [HEK 29...	CRL-11268	16	8, 9	11, 11	12, 14	9, 13	16, 18, 19	7, 9.3	11, 11	11, 12	X, X	15, 17	28, 30.2	17, 18, 19	7, 15, 14	9, 10	12, 14, 11, 15	20, 23	17, 18	19, 19
81.3 %	HEK 293T/17	ACS-4500	16	8, 9	11, 11	12, 14	9, 13	16, 19	7, 9.3	11, 11	11, 12	X, X	15, 17	28, 30.2	17, 18, 19	7, 15, 14	9, 10	12, 14, 11, 15	20, 23	17, 18	19, 19

293T 'B6'

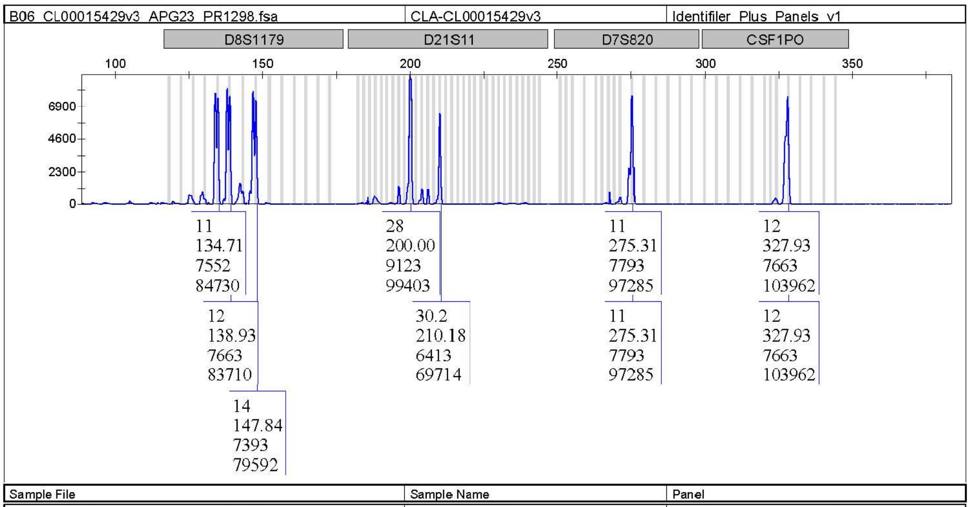

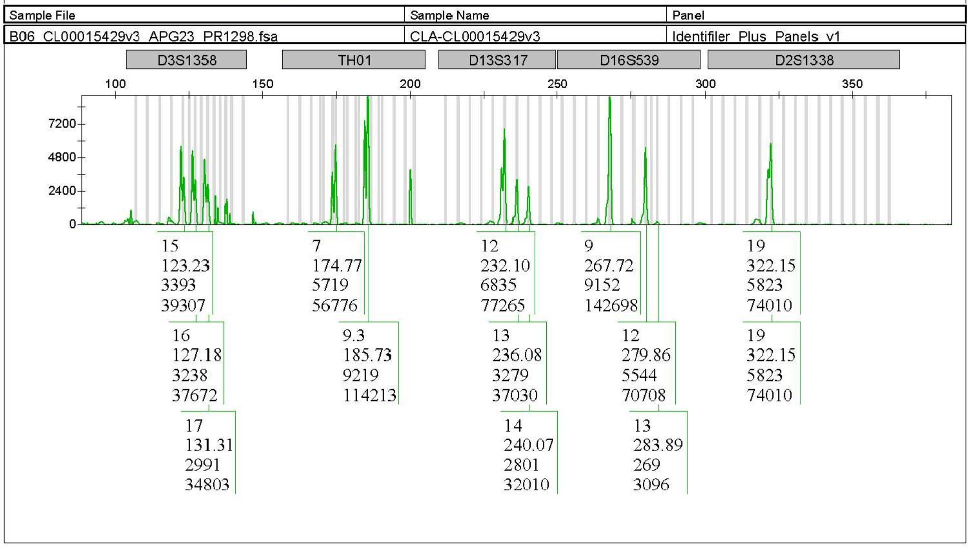

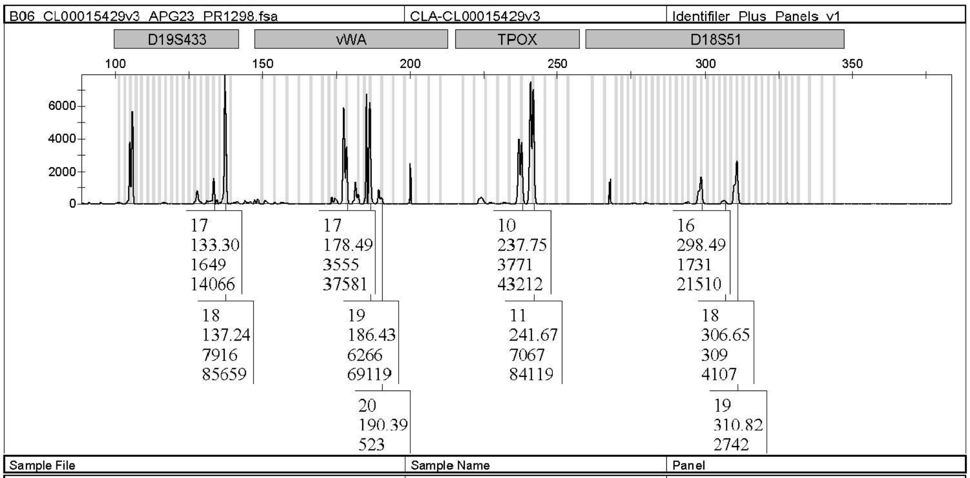

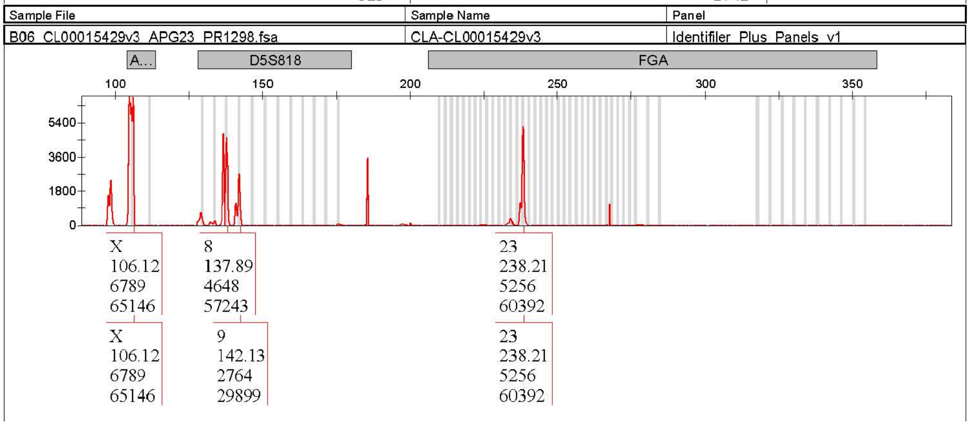

STR Profile Search

Help

The human STR profile database includes data sets of 2455 cell lines from ATCC, DSMZ, JCRB and RIKEN.

Refine search | Start new search

Your search uses the non-empty-based scoring mode.

Similarity	Cell line	Source	Shared	D5S818	D7S820	D13S317	D16S539	vWA	TH01	TPOX	CSF1PO	Amelogenin	D3S1358	D21S11	D18S51	PentaE	PentaD	D8S1179	FGA	D19S433	D2S1338
?		Your query		8, 9	11, 11	12, 13, 14	9, 12	17, 19	7, 9.3	10, 11	12, 12	X, X	15, 16, 17	28, 30.2	16, 19	-	-	11, 12, 14	23, 23	17, 18	19, 19
85.7 %	HEK293T-HELZ-...	DSMZ: ACC-875	16	8, 9	11, 11	12, 14, **11**, 13	9, **13**	**16**, 19	7, 9.3	11, **11**	**11**, 12, **13**	X, X	15, 17, 16, **18**	**27**, 28, 30.2	**17**, **18**, 16	7, 15	9, 10	12, 14, 11	23, 23	17, 18	19, 19
81.8 %	293T/17 [HEK 29...	CRL-11268	16	8, 9	11, 11	12, 14	9, **13**	**16**, **18**, 19	7, 9.3	11, **11**	**11**, 12	X, X	15, 17	28, 30.2	**17**, **18**, 19	7, 15, 14	9, 10	12, 14, 11, **15**	**20**, 23	17, 18	19, 19
81.8 %	HEK 293T/17	ACS-4500	16	8, 9	11, 11	12, 14	9, **13**	**16**, 19	7, 9.3	11, **11**	**11**, 12	X, X	15, 17	28, 30.2	**17**, **18**, 19	7, 15, 14	9, 10	12, 14, 11, **15**	**20**, 23	17, 18	19, 19
81.3 %	293T	DSMZ: ACC-635	16	8, 9	11, 11	12, 14	9, **13**	**16**, 19	7, 9.3	11, **11**	**11**, 12	X, X	15, 17	28, 30.2	**17**, **18**	7, 15	9, 10	12, 14	23, 23	17, 18	19, 19

293T 'B8'

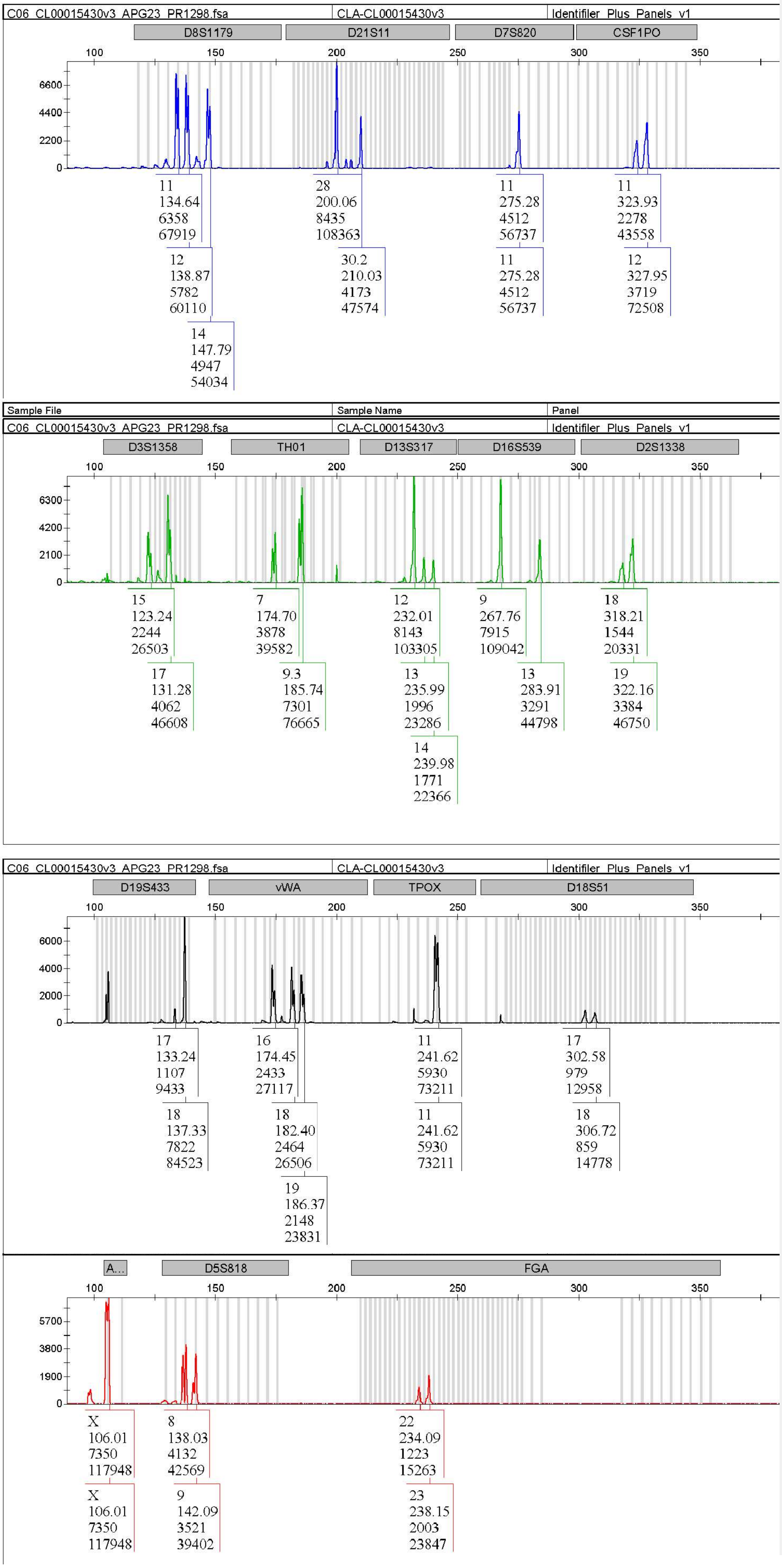

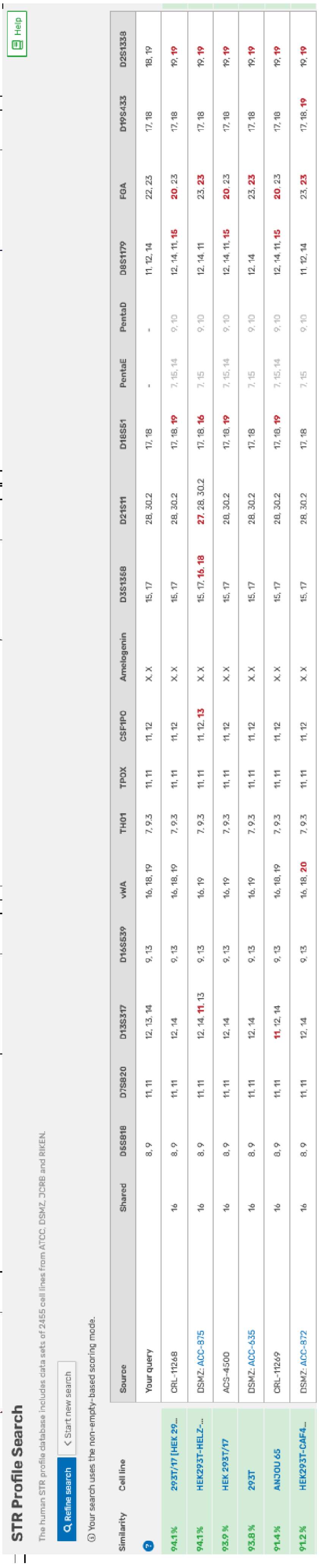

STR Profile Search

The human STR profile database includes data sets of 2455 cell lines from ATCC, DSMZ, JCRB and RIKEN.

Refine search | Start new search

Your search uses the non-empty-based scoring mode.

Similarity	Cell line	Source	Shared	D5S818	D7S820	D13S317	D16S539	vWA	TH01	TPOX	CSF1PO	Amelogenin	D3S1358	D21S11	D18S51	PentaE	PentaD	D8S1179	FGA	D19S433	D2S1338
?		Your query		8, 9	11, 11	12, 13, 14	9, 13	16, 18, 19	7, 9.3	11, 11	11, 12	X, X	15, 17	28, 30.2	17, 18	-	-	11, 12, 14	22, 23	17, 18	18, 19
94.1%	293T/17 [HEK 29...	CRL-11268	16	8, 9	11, 11	12, 14	9, 13	16, 18, 19	7, 9.3	11, 11	11, 12	X, X	15, 17	28, 30.2	17, 18, 19	7, 15, 14	9, 10	12, 14, 11, 15	20, 23	17, 18	19, 19
94.1%	HEK293T-HELZ-...	DSMZ: ACC-875	16	8, 9	11, 11	12, 14, 11, 13	9, 13	16, 19	7, 9.3	11, 11	11, 12, 13	X, X	15, 17, 16, 18	27, 28, 30.2	17, 18, 16	7, 15	9, 10	12, 14, 11	23, 23	17, 18	19, 19
93.9%	HEK 293T/17	ACS-4500	16	8, 9	11, 11	12, 14	9, 13	16, 19	7, 9.3	11, 11	11, 12	X, X	15, 17	28, 30.2	17, 18, 19	7, 15, 14	9, 10	12, 14, 11, 15	20, 23	17, 18	19, 19
93.8%	293T	DSMZ: ACC-635	16	8, 9	11, 11	12, 14	9, 13	16, 19	7, 9.3	11, 11	11, 12	X, X	15, 17	28, 30.2	17, 18	7, 15	9, 10	12, 14	23, 23	17, 18	19, 19
91.4%	ANJOU 65	CRL-11269	16	8, 9	11, 11	11, 12, 14	9, 13	16, 18, 19	7, 9.3	11, 11	11, 12	X, X	15, 17	28, 30.2	17, 18, 19	7, 15, 14	9, 10	12, 14, 11, 15	20, 23	17, 18	19, 19
91.2%	HEK293T-CAF4...	DSMZ: ACC-872	16	8, 9	11, 11	12, 14	9, 13	16, 18, 20	7, 9.3	11, 11	11, 12	X, X	15, 17	28, 30.2	17, 18	7, 15	9, 10	11, 12, 14	23, 23	17, 18, 19	19, 19

293T 'B9'

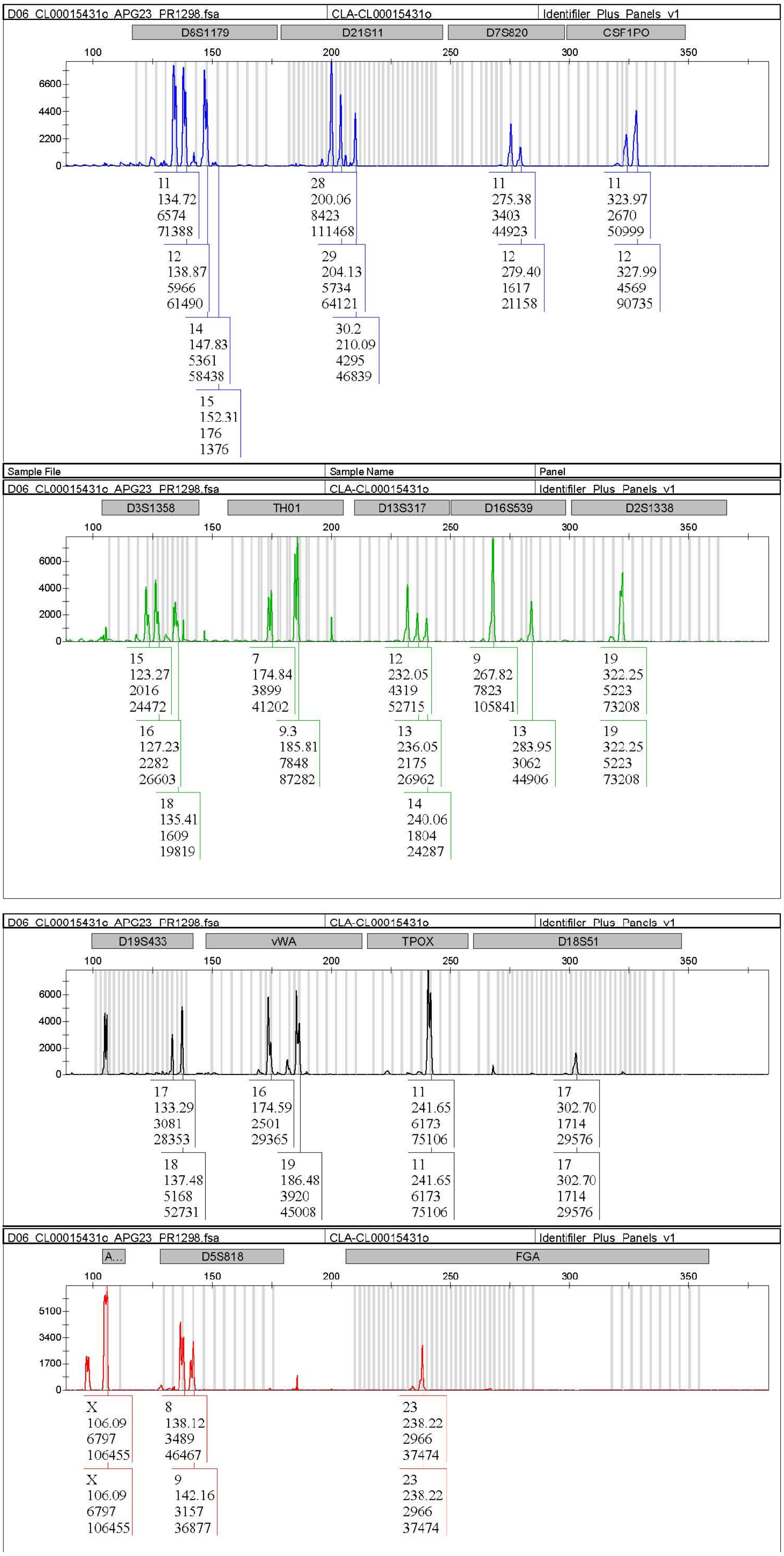

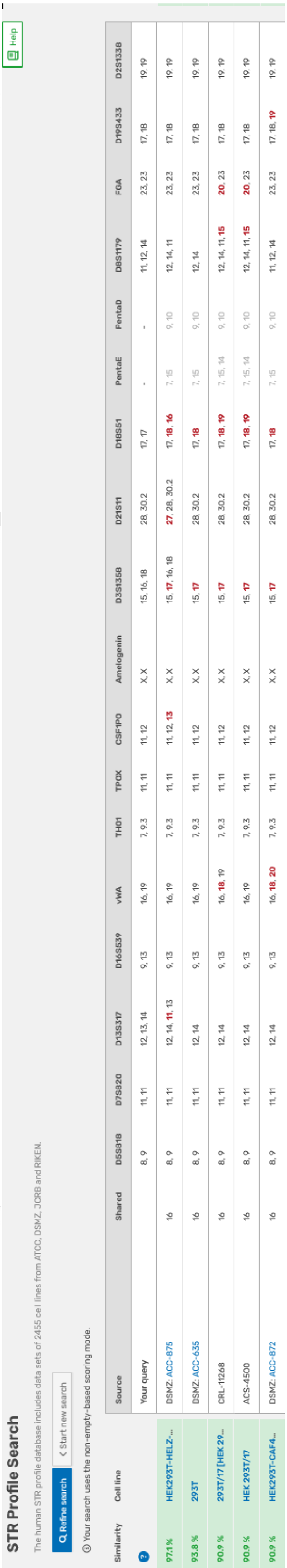

STR Profile Search

The human STR profile database includes data sets of 2455 cell lines from ATCC, DSMZ, JCRB and RIKEN.

Refine search | Start new search | Help

Your search uses the non-empty-based scoring mode.

Similarity	Cell line	Source	Shared	D5S818	D7S820	D13S317	D16S539	vWA	TH01	TPOX	CSF1PO	Amelogenin	D3S1358	D21S11	D18S51	PentaE	PentaD	D8S1179	FGA	D19S433	D2S1338
?		Your query		8, 9	11, 11	12, 13, 14	9, 13	16, 19	7, 9.3	11, 11	11, 12	X, X	15, 16, 18	28, 30.2	17, 17	-	-	11, 12, 14	23, 23	17, 18	19, 19
97.1 %	HEK293T-HELZ...	DSMZ: ACC-875	16	8, 9	11, 11	12, 14, 11, 13	9, 13	16, 19	7, 9.3	11, 11	11, 12, 13	X, X	15, 17, 16, 18	27, 28, 30.2	17, 18, 16	7, 15	9, 10	12, 14, 11	23, 23	17, 18	19, 19
93.8 %	293T	DSMZ: ACC-635	16	8, 9	11, 11	12, 14	9, 13	16, 19	7, 9.3	11, 11	11, 12	X, X	15, 17	28, 30.2	17, 18	7, 15	9, 10	12, 14	23, 23	17, 18	19, 19
90.9 %	293T/17 [HEK 29...	CRL-11268	16	8, 9	11, 11	12, 14	9, 13	16, 18, 19	7, 9.3	11, 11	11, 12	X, X	15, 17	28, 30.2	17, 18, 19	7, 15, 14	9, 10	12, 14, 11, 15	20, 23	17, 18	19, 19
90.9 %	HEK 293T/17	ACS-4500	16	8, 9	11, 11	12, 14	9, 13	16, 19	7, 9.3	11, 11	11, 12	X, X	15, 17	28, 30.2	17, 18, 19	7, 15, 14	9, 10	12, 14, 11, 15	20, 23	17, 18	19, 19
90.9 %	HEK293T-CAF4...	DSMZ: ACC-872	16	8, 9	11, 11	12, 14	9, 13	16, 18, 20	7, 9.3	11, 11	11, 12	X, X	15, 17	28, 30.2	17, 18	7, 15	9, 10	11, 12, 14	23, 23	17, 18, 19	19, 19

293T 'B10'

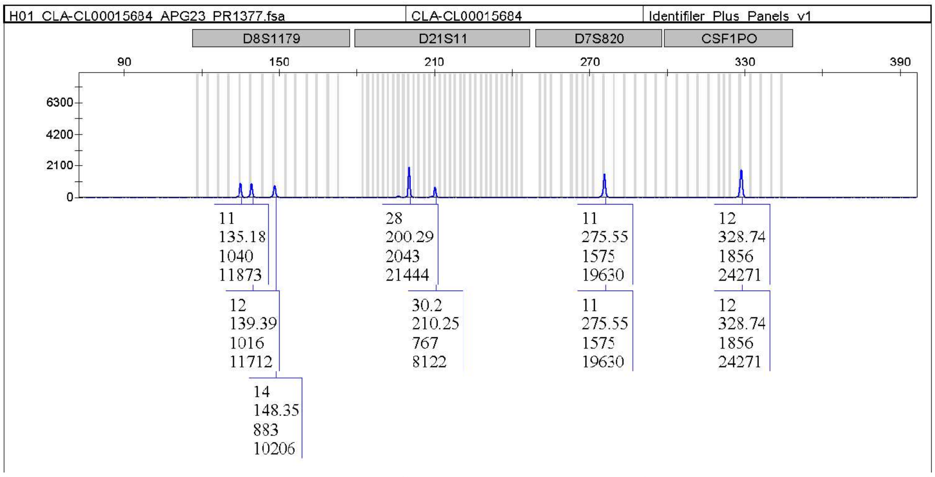

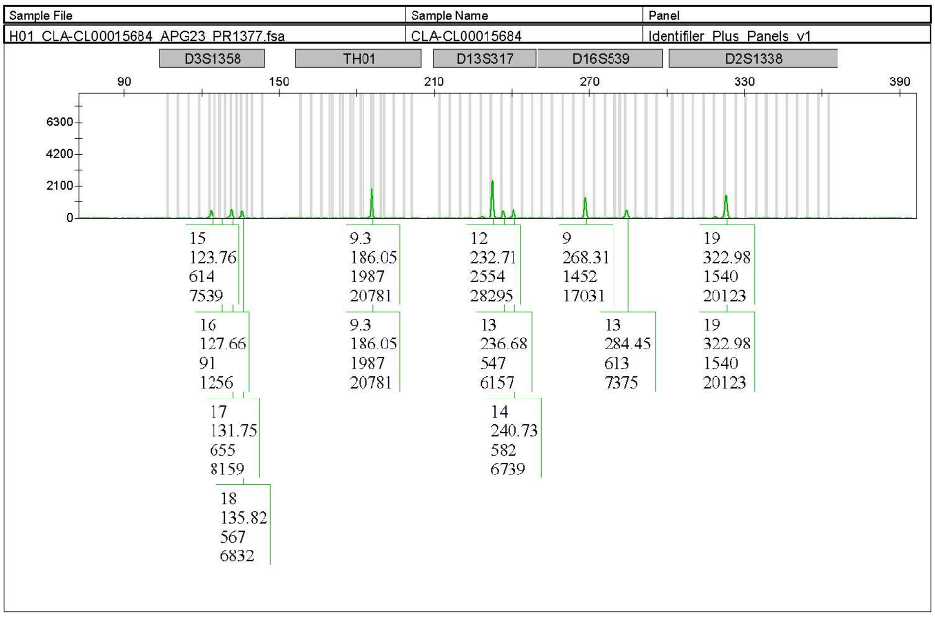

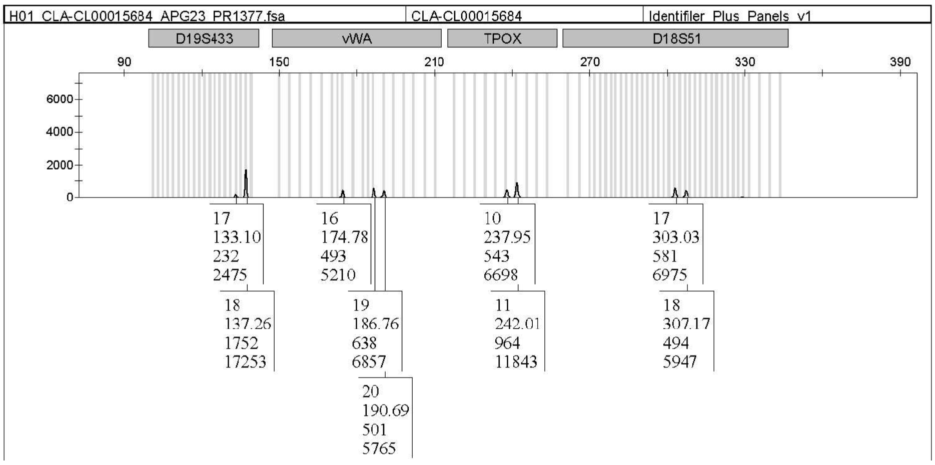

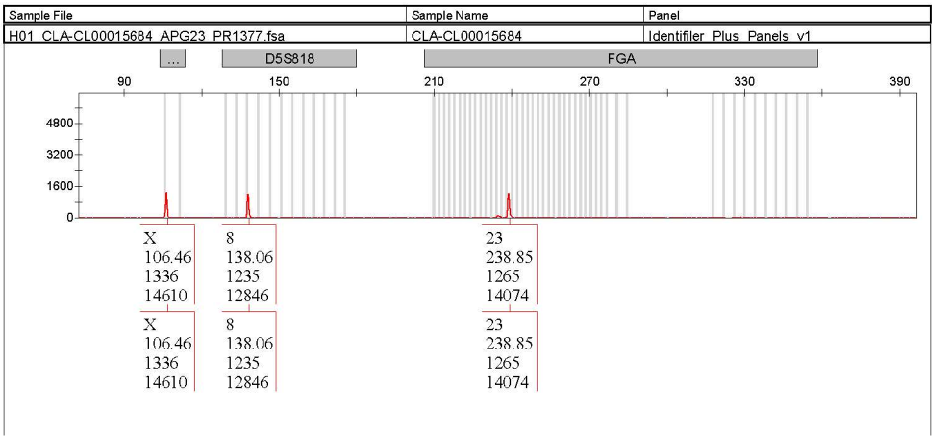

Similarity	Cell line	Source	Shared	D5S818	D7S820	D13S317	D16S539	vWA	TH01	TPOX	CSF1PO	Amelogenin	D3S1358	D21S11	D18S51	PentaE	PentaD	D8S1179	FGA	D19S433	D2S1338
		Your query		8, 8	11, 11	12, 13, 14	9, 13	16, 19, 20	9.3, 9.3	10, 11	12, 12	X, X	15, 17, 18	28, 30.2	17, 18	-	-	11, 12, 14	23, 23	17, 18	19, 19
88.6 %	HEK293T-HELZ-...	DSMZ: ACC-875	16	8, 9	11, 11	12, 14, 11, 13	9, 13	16, 19	7, 9.3	11, 11	11, 12, 13	X, X	15, 17, 16, 18	27, 28, 30.2	17, 18, 16	7, 15	9, 10	12, 14, 11	23, 23	17, 18	19, 19
87.5 %	293T	DSMZ: ACC-635	16	8, 9	11, 11	12, 14	9, 13	16, 19	7, 9.3	11, 11	11, 12	X, X	15, 17	28, 30.2	17, 18	7, 15	9, 10	12, 14	23, 23	17, 18	19, 19
85.3 %	HEK293T-CAF4...	DSMZ: ACC-872	16	8, 9	11, 11	12, 14	9, 13	16, 18, 20	7, 9.3	11, 11	11, 12	X, X	15, 17	28, 30.2	17, 18	7, 15	9, 10	11, 12, 14	23, 23	17, 18, 19	19, 19
84.8 %	HEK 293T/17	ACS-4500	16	8, 9	11, 11	12, 14	9, 13	16, 19	7, 9.3	11, 11	11, 12	X, X	15, 17	28, 30.2	17, 18, 19	7, 15, 14	9, 10	12, 14, 11, 15	20, 23	17, 18	19, 19
84.4 %	HEK293-DR-GF...	DSMZ: ACC-854	16	8, 8	11, 12	12, 14	9, 13	16, 19	7, 9.3	11, 11	12, 12	X, X	15, 17	30.2, 30.2	17, 18	7, 15	9, 9	12, 14	23, 23	18, 18	19, 19
84.4 %	HEK293-DR-GF...	DSMZ: ACC-850	16	8, 8	11, 12	12, 14	9, 13	16, 19	7, 9.3	11, 11	12, 12	X, X	15, 17	28, 30	17, 18	7, 15	9, 10	12, 14	23, 23	18, 18	19, 19

293T 'B15'

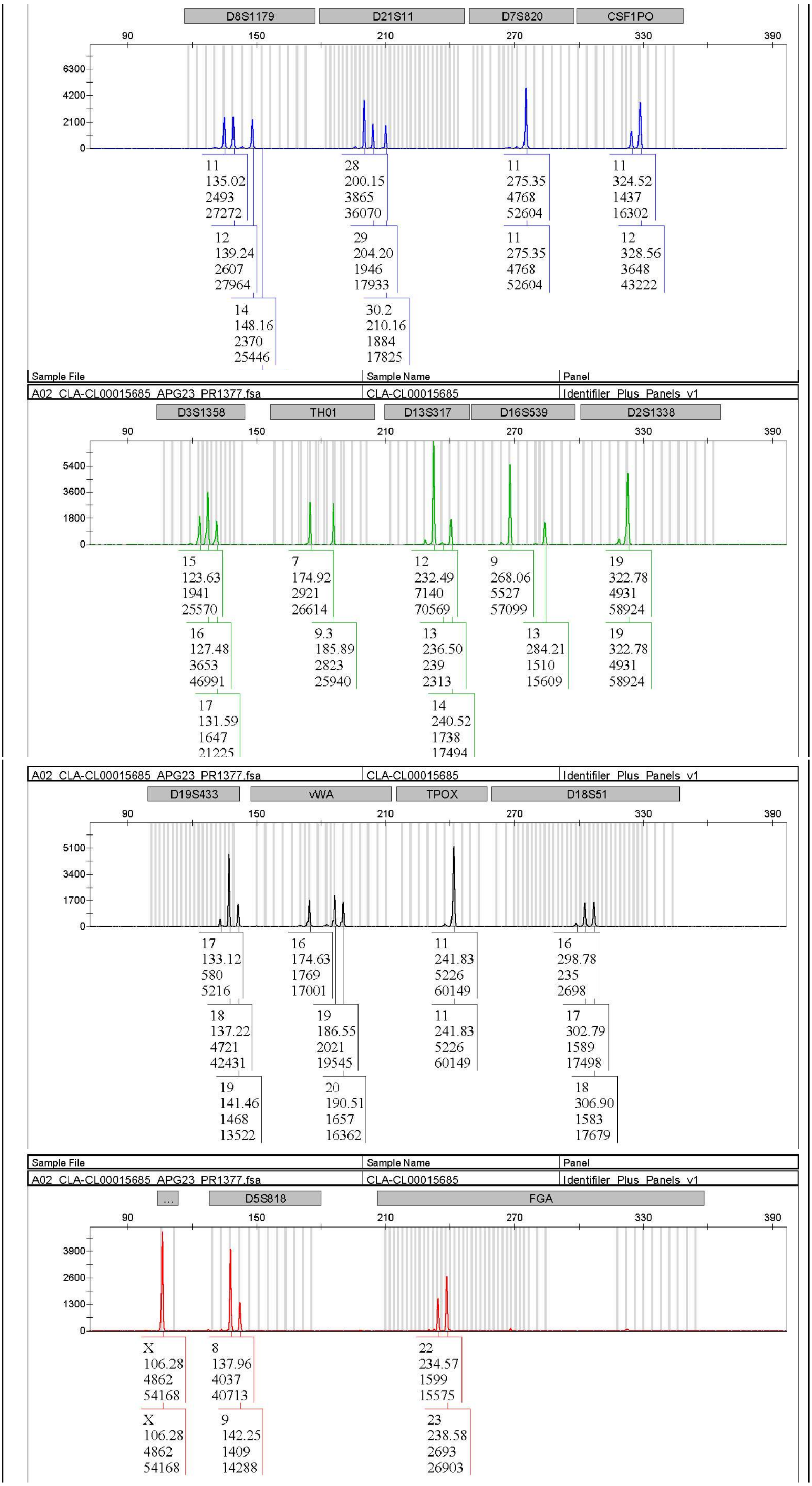

Similarity	Cell line	Source	Shared	D5S818	D7S820	D13S317	D16S539	vWA	TH01	TPOX	CSF1PO	Amelogenin	D3S1358	D21S11	D18S51	PentaE	PentaD	D8S1179	FGA	D19S433	D2S1338
		Your query		8, 9	11, 11	12, 14	9, 13	16, 19, 20	7, 9.3	11, 11	11, 12	X, X	15, 16, 17	28, 29, 30.2	16, 17, 18	-	-	11, 12, 14	22, 23	17, 18, 19	19, 19
96.9 %	293T	DSMZ: ACC-635	16	8, 9	11, 11	12, 14	9, 13	16, 19	7, 9.3	11, 11	11, 12	X, X	15, 17	28, 30.2	17, 18	7, 15	9, 10	12, 14	23, **23**	17, 18	19, 19
94.4 %	HEK293T-HELZ-...	DSMZ: ACC-875	16	8, 9	11, 11	12, 14, **11**, **13**	9, 13	16, 19	7, 9.3	11, 11	11, 12, **13**	X, X	15, 17, 16, **18**	**27**, 28, 30.2	17, 18, 16	7, 15	9, 10	12, 14, 11	23, **23**	17, 18	19, 19
94.3 %	HEK293T-CAF4...	DSMZ: ACC-872	16	8, 9	11, 11	12, 14	9, 13	16, **18**, 20	7, 9.3	11, 11	11, 12	X, X	15, 17	28, 30.2	17, 18	7, 15	9, 10	11, 12, 14	23, **23**	17, 18, 19	19, 19
94.1 %	HEK 293T/17	ACS-4500	16	8, 9	11, 11	12, 14	9, 13	16, 19	7, 9.3	11, 11	11, 12	X, X	15, 17	28, 30.2	17, 18, **19**	7, 15, 14	9, 10	12, 14, 11, **15**	**20**, 23	17, 18	19, 19

HeLa 'HA'

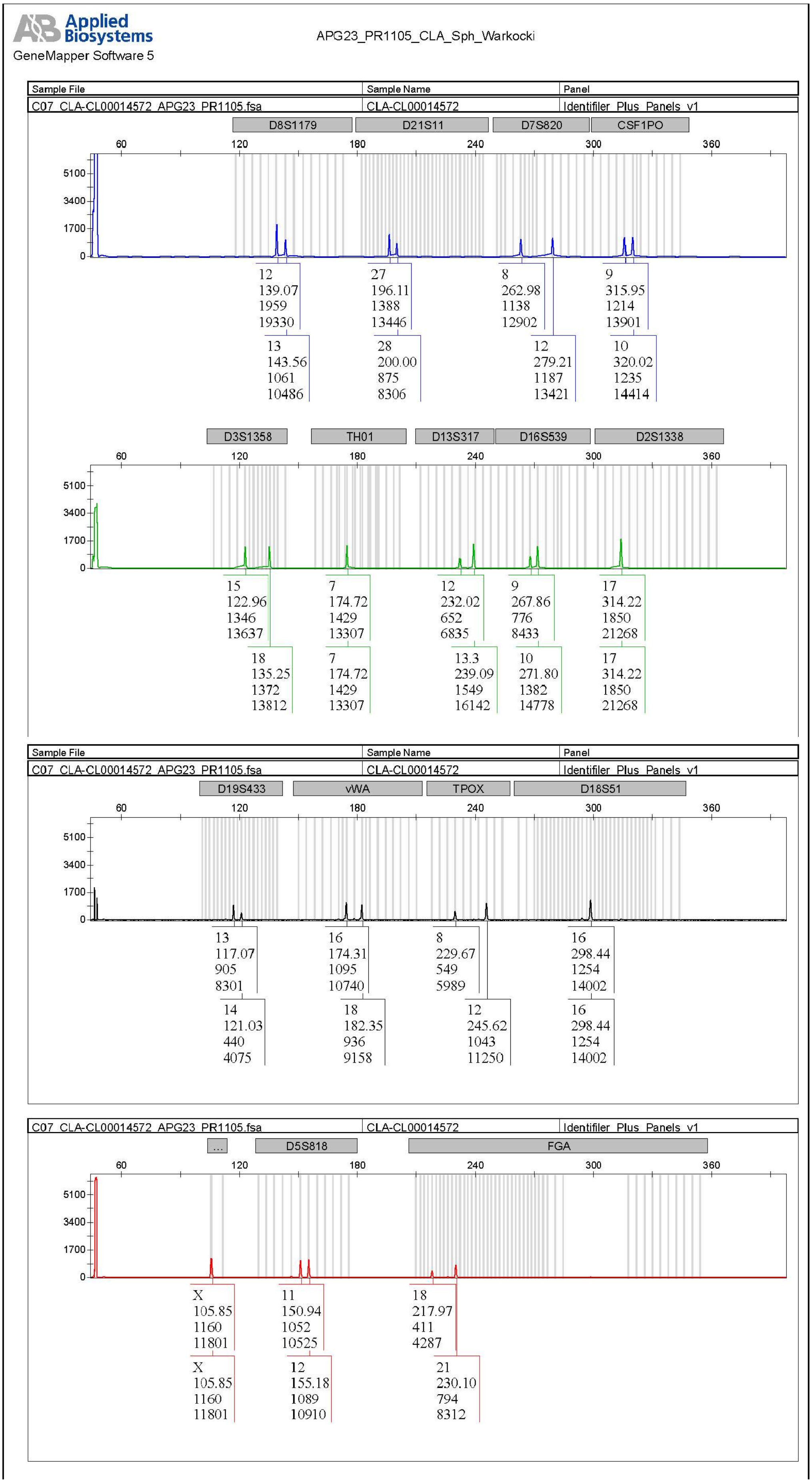

STR Profile Search

The human STR profile database includes data sets of 2455 cell lines from ATCC, DSMZ, JCRB and RIKEN.

Refine search | Start new search

Your search uses the non-empty-based scoring mode.

Similarity	Cell line	Source	Shared	D5S818	D7S820	D13S317	D16S539	vWA	TH01	TPOX	CSF1PO	Amelogenin	D3S1358	D21S11	D18S51	PentaE	PentaD	D8S1179	FGA	D19S433	D2S1338
?		Your query		11, 12	8, 12	12, 13.3	9, 10	16, 18	7, 7	8, 12	9, 10	X, X	15, 18	27, 28	16, 16	-	-	12, 13	18, 21	13, 14	17, 17
100 %	HeLa.S3	RCB1525	16	11, 12	8, 12	12, 13.3	9, 10	16, 18	7, 7	8, 12	9, 10	X, X	15, 18	27, 28	16, 16	7, 17	8, 14	12, 13	18, 21	13, 14	17, 17
100 %	HeLa.S3	RCB0191	16	11, 12	8, 12	12, 13.3	9, 10	16, 18	7, 7	8, 12	9, 10	X, X	15, 18	27, 28	16, 16	7, 17	8, 14	12, 13	18, 21	13, 14	17, 17

HeLa 'HA'
A17 XRN1 KO

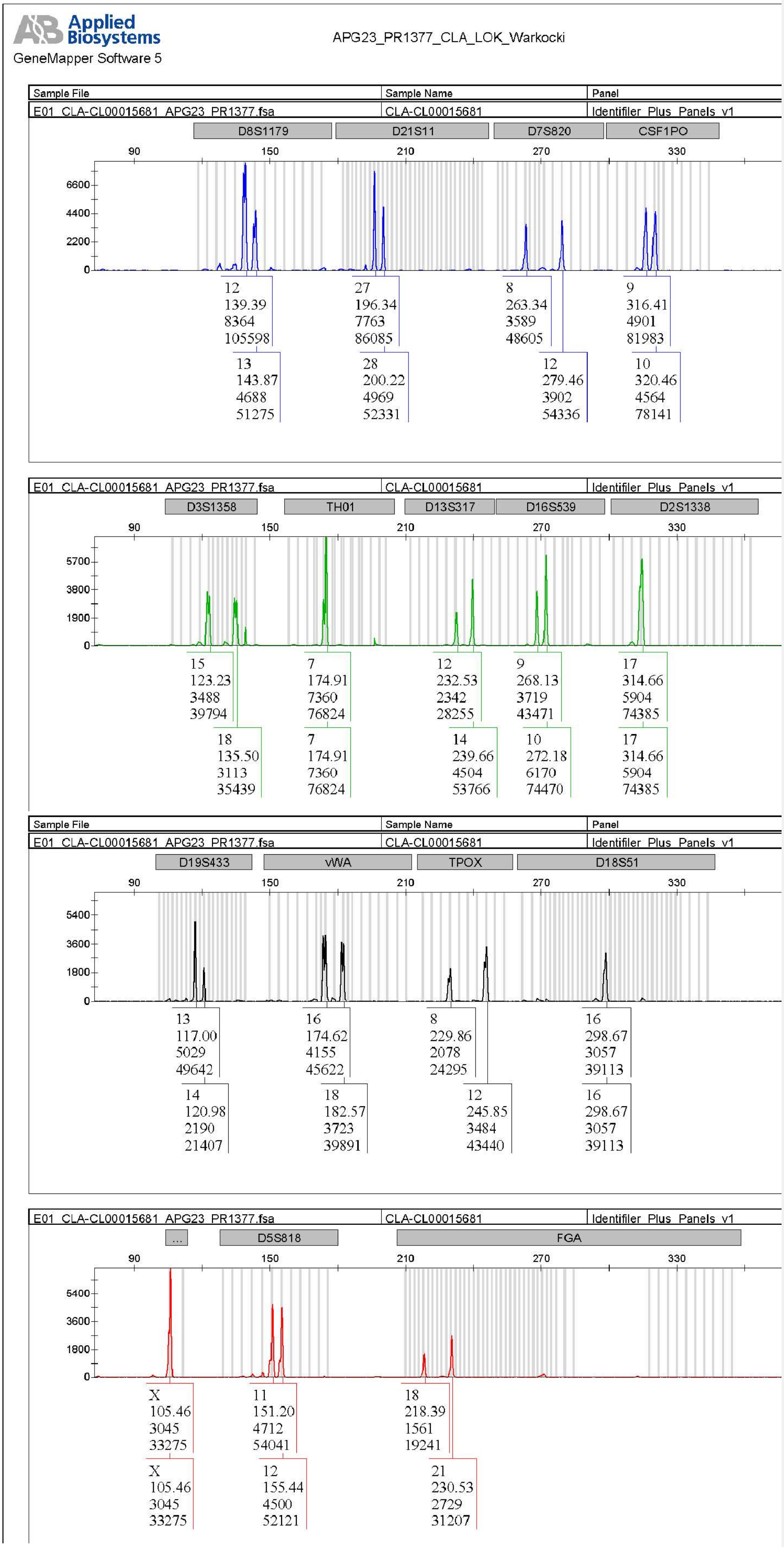

Similarity	Cell line	Source	Shared	D5S818	D7S820	D13S317	D16S539	vWA	TH01	TPOX	CSF1PO	Amelogenin	D3S1358	D21S11	D18S51	PentaE	PentaD	D8S1179	FGA	D19S433	D2S1338
		Your query		11, 12	8, 12	12, 14	9, 10	16, 18	7, 7	8, 12	9, 10	X, X	15, 18	27, 28	16, 16	-	-	12, 13	18, 21	13, 14	17, 17
100 %	WRL 68 (HeLa d...	HPACC	16	11, 12	8, 12	12, 14	9, 10	16, 18	7, 7	8, 12	9, 10	X, X	15, 18	27, 28	16, 16	7, 17	8, 15	12, 13	18, 21	13, 14	17, 17
100 %	L132 (HeLa deriv...	HPACC	16	11, 12	8, 12	12, 14	9, 10	16, 18	7, 7	8, 12	9, 10	X, X	15, 18	27, 28	16, 16	7, 17	8, 15	12, 13	18, 21	13, 14	17, 17
100 %	HeLa/MMTV-luc	HPACC	16	11, 12	8, 12	12, 14	9, 10	16, 18	7, 7	8, 12	9, 10	X, X	15, 18	27, 28	16, 16	7, 17	8, 15	12, 13	18, 21	13, 14	17, 17
100 %	HeLa	HPACC	16	11, 12	8, 12	12, 14	9, 10	16, 18	7, 7	8, 12	9, 10	X, X	15, 18	27, 28	16, 16	7, 17	8, 15	12, 13	18, 21	13, 14	17, 17
100 %	Chang Liver (He...	HPACC	16	11, 12	8, 12	12, 14	9, 10	16, 18	7, 7	8, 12	9, 10	X, X	15, 18	27, 28	16, 16	7, 17	8, 15	12, 13	18, 21	13, 14	17, 17
100 %	AV3 (HeLa deriv...	HPACC	16	11, 12	8, 12	12, 14	9, 10	16, 18	7, 7	8, 12	9, 10	X, X	15, 18	27, 28	16, 16	7, 17	8, 15	12, 13	18, 21	13, 14	17, 17
96.9 %	HeLa-CD4-LTR-...	RCB2356	16	11, 12	8, 12	12, **12**	9, 10	16, 18	7, 7	8, 12	9, 10	X, X	15, 18	27, 28	16, 16	7, 17	8, 14	12, 13	18, 21	13, 14	17, 17
96.9 %	HeLa.S3	RCB1525	16	11, 12	8, 12	12, **13.3**	9, 10	16, 18	7, 7	8, 12	9, 10	X, X	15, 18	27, 28	16, 16	7, 17	8, 14	12, 13	18, 21	13, 14	17, 17
96.9 %	HeLa-P3	RCB0402	16	11, 12	8, 12	12, **12**	9, 10	16, 18	7, 7	8, 12	9, 10	X, X	15, 18	27, 28	16, 16	7, 17	8, 14	12, 13	18, 21	13, 14	17, 17
96.9 %	HeLa.S3	RCB0191	16	11, 12	8, 12	12, **13.3**	9, 10	16, 18	7, 7	8, 12	9, 10	X, X	15, 18	27, 28	16, 16	7, 17	8, 14	12, 13	18, 21	13, 14	17, 17

PA-1

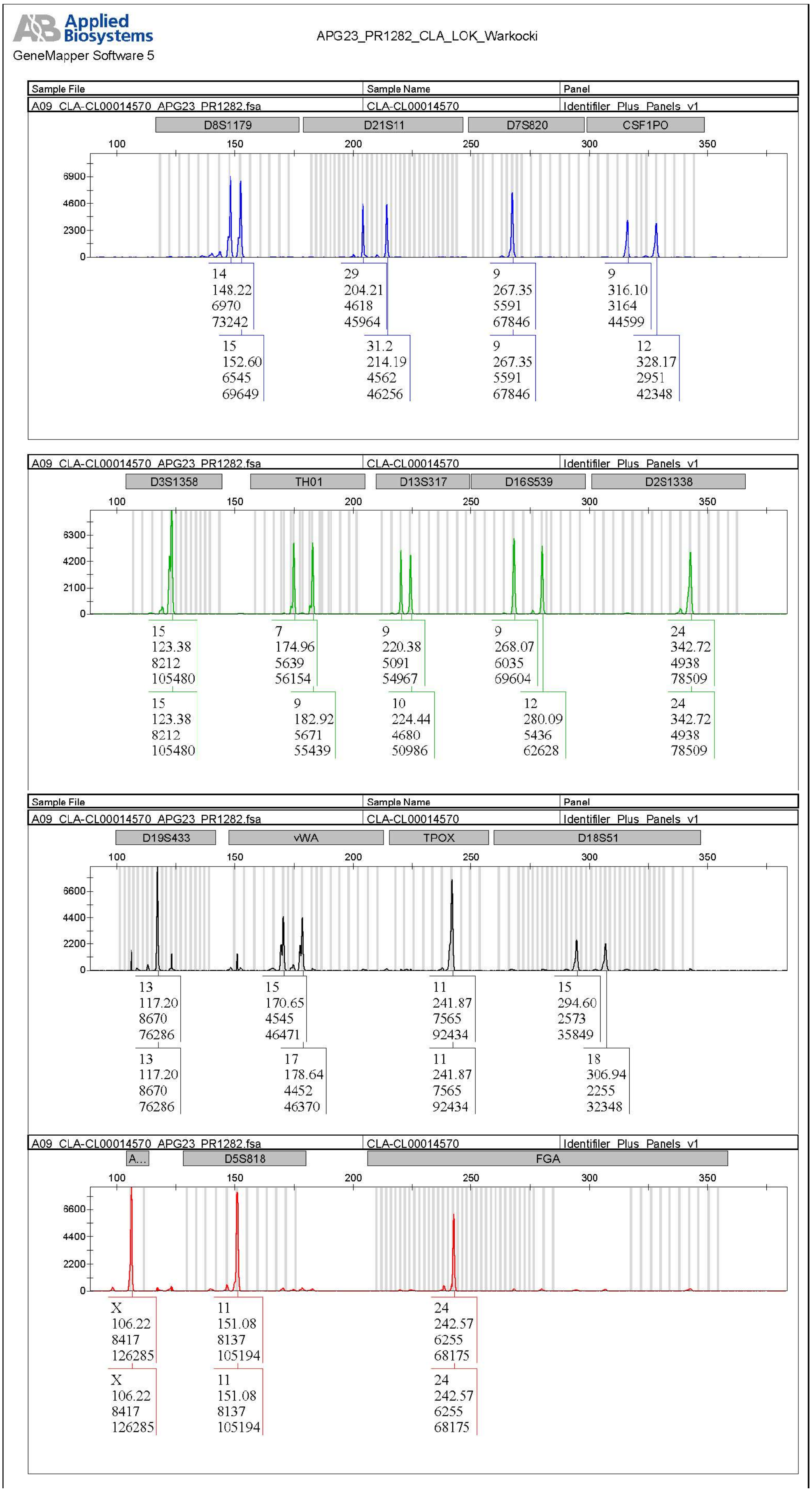

STR Profile Search

The human STR profile database includes data sets of 2455 cell lines from ATCC, DSMZ, JCRB and RIKEN.

Refine search | Start new search

Your search uses the non-empty-based scoring mode.

Similarity	Cell line	Source	Shared	D5S818	D7S820	D13S317	D16S539	vWA	TH01	TPOX	CSF1PO	Amelogenin	D3S1358	D21S11	D18S51	PentaE	PentaD	D8S1179	FGA	D19S433	D2S1338
?		Your query		11, 11	9, 9	9, 10	9, 12	15, 17	7, 9	11, 11	9, 12	X, X	15, 15	29, 31.2	15, 18	-	-	14, 15	24, 24	13, 13	24, 24
100 %	PA-1	RCB1946	16	11, 11	9, 9	9, 10	9, 12	15, 17	7, 9	11, 11	9, 12	X, X	15, 15	29, 31.2	15, 18	14, 20	9, 12	14, 15	24, 24	13, 13	24, 24
100 %	PA-1	JCRB9061	16	11, 11	9, 9	9, 10, 13	9, 12	15, 17	7, 9	11, 11	9, 12	X, X	15, 15	29, 31.2	15, 18	14, 20	9, 12	14, 15	24, 24	13, 13	24, 24
100 %	PA-1	HPACC	16	11, 11	9, 9	9, 10	9, 12	15, 17	7, 9	11, 11	9, 12	X, X	15, 15	29, 31.2	15, 18	14, 20	9, 12	14, 15	24, 24	13, 13	24, 24
100 %	PA-1	CRL-1572	16	11, 11	9, 9	9, 10	9, 12	15, 17	7, 9	11, 11	9, 12	X, X	15, 15	29, 31.2	15, 18	14, 20	9, 12	14, 15	24, 24	13, 13	24, 24
